# Change in fish functional diversity and assembly rules in the course of tidal marsh restoration

**DOI:** 10.1371/journal.pone.0209025

**Published:** 2018-12-19

**Authors:** Alain Lechêne, Jérémy Lobry, Philippe Boët, Pascal Laffaille

**Affiliations:** 1 Irstea, UR EABX, centre de Bordeaux, 50 avenue de Verdun, F-33612 Cestas cedex, France; 2 EcoLab, Université de Toulouse, INP, UPS, ENSAT, Castanet-Tolosan, France; University of Sydney, AUSTRALIA

## Abstract

Functional trait theory provides a mechanistic framework to understand change in community composition and community assembly through time and space. Despite this, trait-based approaches have seldom been used in ecological restoration. Succession theory predicts that habitat complexity and resource availability will increase with restoration time, leading to increased functional dissimilarity among coexisting species. However, in the case of tidal marsh restoration, it is not clear whether reestablishing the harsh abiotic conditions typical of estuaries will initiate successional trajectories. We investigated monotonic changes in the functional structure of fish communities and shifts in assembly mechanisms, with tidal restoration time. A five-level gradient of ‘intertidal habitat naturalness’ was constructed from a set of artificialized (dyked), restored (with different ages) and natural intertidal sites, and used as a surrogate for restoration progress. The fish ecophases were described using ten functional traits related to food acquisition and swimming ability. The trends in six functional dimensions (identity, richness, evenness, dispersion, originality and specialization) were investigated along the naturalness gradient. Consistenly with succession theory, functional specialization, dispersion and, less markedly, richness increased with intertidal naturalness meaning that restored and natural intertidal habitats supplied fish with specific foraging and dwelling conditions absent from dyked marshes. Community assembly patterns varied with respect to traits and differed at both ends of the naturalness gradient. Dyked marshes were more affected by trait convergence possibly due to limiting resources. Environmental filtering was detected all along the naturalness gradient although the traits affected varied depending on the naturalness level of habitats. Environmental filtering tended to decrease in restored and natural intertidal habitats. Increased naturalness restored the attractivity of benthic habitats as feeding or settling grounds, promoted shelter-seeking *vs*. free-swimming strategists and favoured ecophases with carnivorous diets, feeding on microinvertebrates and benthic low-mobility macroinvertebrates. Approaches based on functional trait diversity have the potential to question and refine the theoretical frame of ecological restoration and to assist managers in their efforts to restore tidal wetlands.

## Introduction

Functional traits determine the biological and ecological performance of an organism, i.e. its ability to grow, survive and reproduce in a given set of biotic and abiotic conditions [1, 2]. The distribution of functional traits’ values among individuals defines the functional structure of a community [3, 4]. Because functional traits mediate the interaction between an organism and its abiotic and biotic environment, their analysis provides a mechanistic framework from which the ecological (i.e. niche-based) processes driving community composition and community assembly can be inferred. It is a great advantage over taxonomic approaches, which do not readily reveal the processes underlying community changes through time and space (e.g., [5–8]). In recent years, trait-based approaches have been used to investigate changes in both the functional structure of communities and patterns of community assembly along temporal (e.g., [9, 10]) and environmental gradients (e.g., [11–13]).

**Fig 1:**
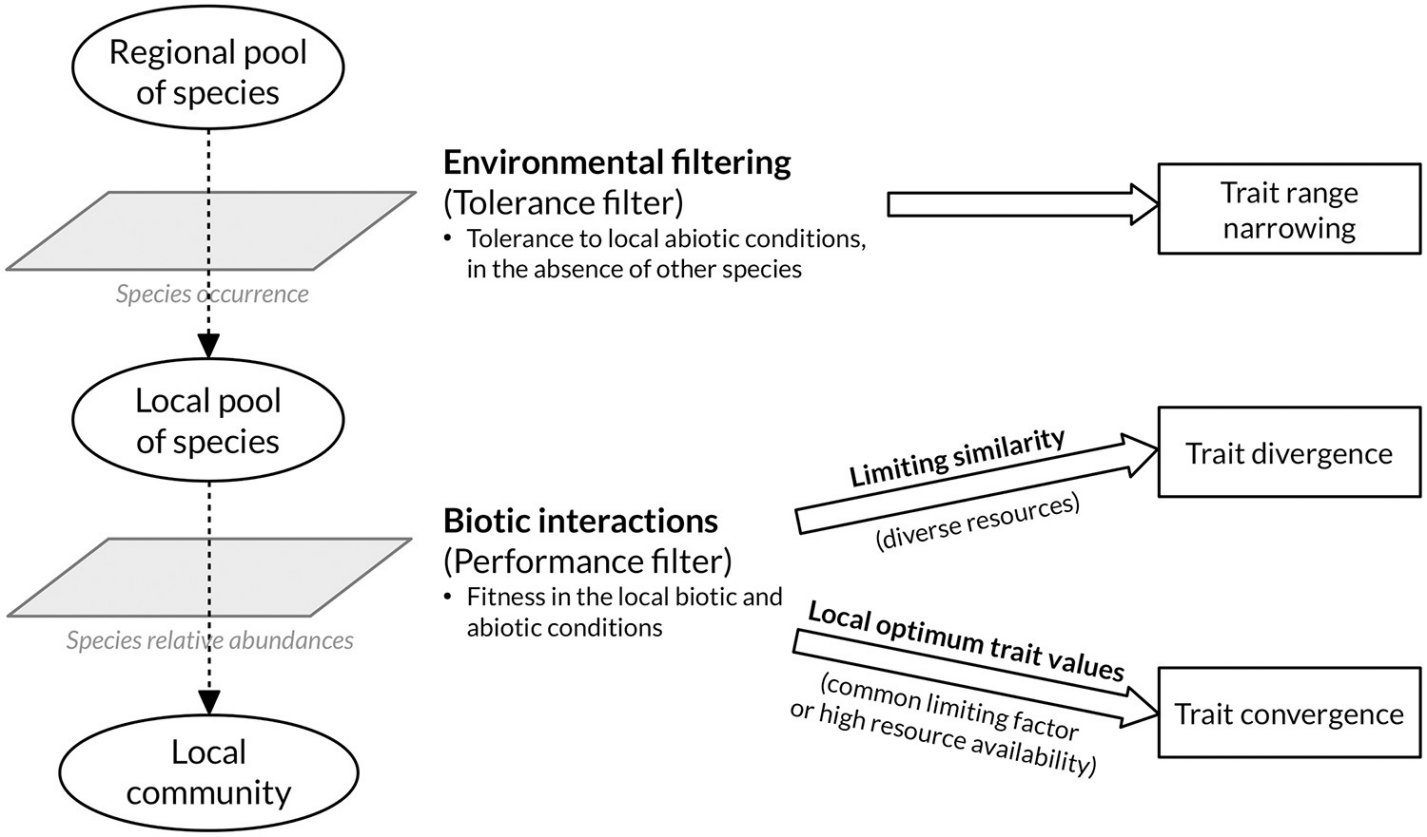
The two-step hierarchical model of community assembly and the effects of the ecological filters on functional trait distribution [14, 15].

Community assembly theory states that local communities assemble non randomly from a regional pool of species as a result of environmental filtering and biotic interactions. Species assemble following a two-step hierarchical model ([14, 15]; Fig 1). First, a tolerance filter (’environmental filtering’ *sensu stricto*; [16]) selects species according to their ability to establish and persist in the local abiotic conditions, in the absence of biotic interactions. Environmental filtering primarily affects the occurrence (i.e. presence or absence) of the species on the local scale [16]. Retained species tend to share similar values for traits associated with abiotic tolerance, resulting in trait range narrowing. In a second step, species interact with each other in the local environment. Trait-based fitness differences determine the outcome of those interactions, i.e. the relative abundance of species in the community. Biotic interactions, especially competition, can result in two constrasted patterns of functional traits’ distribution, i.e. divergence or convergence [15, 17]. For a given range of functional trait values, divergence is defined by higher dispersion of trait values compared to random expectation. Reciprocally, convergence occurs where traits are less dispersed (i.e., more clustered) than expected at random. Competitive interactions are stronger between species with high niche overlap, i.e. sharing similar functional traits’ values. When resources and foraging opportunities are diverse, competition excludes species with high niche overlap resulting in limiting similarity (i.e., trait divergence) among coexisting species. Conversely, trait convergence arises when overall fitness is determined by a common limiting factor, e.g. a resource or a shared predator. In such cases, high divergence from the local optimum traits values leads to weaker competitor exclusion based on relative fitness differences [14–16, 18]. However, in contrast with this explanation of trait convergence, the coexistence of functionally similar species was recently hypothesized to be promoted by elevated resource opportunies (i.e. high resource diversity and/or availability; [13]). Therefore, trait convergence must be interpreted with caution. In addition, the setting of the regional and local scales is crucial when investigating for environmental filtering and rules of species coexistence [19, 20]. Indeed, the probability to detect environmental filtering increases with the extent of the geographic area from which the regional pool of species is defined due to wider abiotic gradients and increased dispersal limitations [16, 19]. Conversely, the effects of biotic interactions prevail at finer spatial scales [21].

In recent years, trait-based approaches have increasingly been used to document the ecological shifts in the course of succession [9, 10, 22] or in response to disturbances such as habitat degradation [5, 23], climate change [24] or invasion by exotic species [25, 26]. The literature dedicated to the effects of anthropogenic disturbances on functional diversity has reached two main conclusions. First, disturbance leads to biotic homogenization due to the higher vulnerability of specialized species (i.e. with specialized traits) and the stronger resistance of generalist (i.e. with common traits) species [5, 24, 26]. Second, disturbance decreases functional redundancy, i.e. primarily causes the extinction of functionally similar species. Low redundancy causes the loss of individuals or species to greatly affect the other dimensions (e.g., richness, evenness and divergence) of the functional structure and alters the stability of the community [4, 27]. In comparison, the functional response of communities to restoration has remained largely unexplored [7, 28]. Increasing functional diversity is not an automatic goal for ecological restoration [7].

Fish are highly mobile organisms granting them the ability to evade adverse abiotic conditions and biotic interactions when more suitable alternative habitats are available. As a result, the relative importance of the processes governing fish assembly may vary over short time scales [21]. Approaches based on the functional trait diversity of fish assemblages have been carried out in varied types of aquatic ecosystems including streams [20, 21, 24], lakes, reservoirs, ponds [13, 26, 29], coastal lagoons [5, 30, 31], coral reefs [27], semi-enclosed sea [32] and estuaries [33, 34]. Limiting similarity strongly structures fish assemblages in lakes and reservoirs [26, 29]. In contrast, fish assemblages of streams are mainly assembled through environmental filtering [20] although biotic interactions may occasionally offset the effects of environmental filtering [21]. Likewise, fish community assembly is essentially an environmentally-driven process in coastal lagoons [30, 31, 35] and marine ecosystems affected by strong salinity gradients [32]. However, biotic interactions may be equally or more important than environmental filtering in community assembly in marine ecosystems where environmental conditions do not vary steeply over short spatial scales [32].

Estuaries are highly productive environments providing considerable amounts of food to a wide range of consumers [36]. At the same time, estuaries are strongly shaped by several abiotic factors that fluctuate widely over short space and time scales, especially salinity [37]. This explains why natural estuarine aquatic communities are simultaneously species-poor and abundance-rich [38]. As a result of those concomitant attributes, it is often assumed that estuarine communities are weakly shaped, if at all, by interspecific competition, but potentially affected by intraspecific competition [39]. Although trait-environment relationships have been compared among fish communities within estuaries [33], assembly patterns (i.e. environmental filtering and biotic interactions) have not yet been evaluated on that scale, to our knowledge. In addition, intra-estuarine studies on fish functional diversity have mainly focused on the longitudinal gradient (i.e. salinity gradient) without considering the mosaic of intertidal and marshland habitats [33].

In temperate estuaries, mudflats and marshes make up the most of intertidal areas and serve as important refuge and feeding grounds for transient juvenile and resident adult fish [40–42]. However, because of land claim, intertidal wetlands have greatly declined worldwide since the 18th century in favour of impounded marshlands. Compared to intertidal marshes and mudflats, the man-made creek networks of dyked marshes offer a lower surface of more perennial (i.e. not completely drained when the tide recedes) aquatic habitats with reduced estuarine connectivity [43]. Tidal restriction markedly alters the taxonomic composition of fish communities and promotes freshwater and non-native species [44–47]. To counteract the loss of intertidal wetlands, a growing number of restoration projects have been implemented during the past decades, first on the Atlantic and Pacific Coasts of the US and later in Western Europe [48, 49]. Fish communities of tidally restored marshes rapidly depart from dyked marshes (strong and rapid shift in taxonomic composition; [50, 51]). However, little is known concerning the functional structure of fish assemblages in dyked and tidally restored marshes. This knowledge would help identify the main differences in ecological functioning among artificialized, restored and natural intertidal habitats.

Unassisted restoration is expected to initiate an ecological trajectory similar to that of succession and opposite to that of degradation. According to one line of reasoning, the functional diversity of communities increases with restoration time, as a result of increased resource availability, higher habitat complexity and higher proportion of specialist species, i.e. with stress-tolerant and competitive strategies [10, 22]. Furthermore, the dominating processes of community assembly are expected to shift from environmental filtering to biotic interactions [52, 53]. However, in the case of tidal marsh restoration, opposing forces associated with the peculiarities of estuarine environments may counteract the general expectations or lead to contrasted patterns with respect to traits. Firstly, as the endpoint of tidal restoration is an estuarine intertidal habitat (i.e. with harsh abiotic conditions), an overlaying signal of environmental filtering is still expected to shape fish assemblages in restored and natural intertidal habitats; however, the most influential abiotic factors controlling communities may differ between dyked marshes and natural intertidal habitats. Secondly, due to the harsh abiotic conditions and the changing food availability and foraging conditions in estuaries, the estuarine biota is naturally comprised of generalist species in terms of physiological tolerance and feeding strategies [39, 40]. Therefore, it is not clear whether tidal marsh restoration will increase functional specialization within fish assemblages.

We studied the tidal restoration of formerly dyked marshes in a European macrotidal estuary based on the functional structure of fish assemblages. The directional changes in the functional structure and the assembly patterns of fish communities during tidal marsh restoration were investigated using functional diversity indices and null models of community assembly. We asked two main questions: 1) Does the functional structure of fish assemblages change monotonically in the course of tidal marsh restoration? We anticipated an increase in functional richness and specialization with increasing restoration time, reflecting higher niche availability (food, habitat) and more specialized niches. 2) Which processes govern community assembly in dyked, restored and intertidal habitats? Do assembly patterns change in the course of tidal marsh restoration? We did not clearly anticipate the outcome of the antagonist processes associated with ecosystem development (i.e. lower environmental filtering and stronger biotic interactions) and return to a highly fluctuating abiotic environment (i.e. higher environmental filtering and weaker biotic interactions). However, trait convergence associated with limiting food resources was expected to decrease with increasing restoration time. In our study, restoration time was approximated based on the naturalness status of a range of artificialized, restored and natural intertidal sites. Fish species were subdivided into several ecophases to account for the major ecological shifts that occur during ontogeny [54, 55]. To our knowledge, this is one of the first times that ontogenetic changes are accounted for on a whole-community scale (but see [13, 56]).

## Materials and methods

### Study sites and fish sampling

The Gironde (France) is the largest estuary in Western Europe. The surface area of the subtidal and natural intertidal zones of the estuary amounts to 635 km^2^ (86% subtidal, 14% intertidal). The dyked marshlands on the lateral zones and the islands occupy an additional area of about 400 km^2^. The share of mudflats and marshes in the natural intertidal zone amounts to 79% and 21%, respectively [57]. The Gironde estuary is characterized by semi-diurnal macrotides. Tidal range near the mouth varies from 2.5 m during neap tides to more than 5.5 m during spring tides [58].

**Fig 2:**
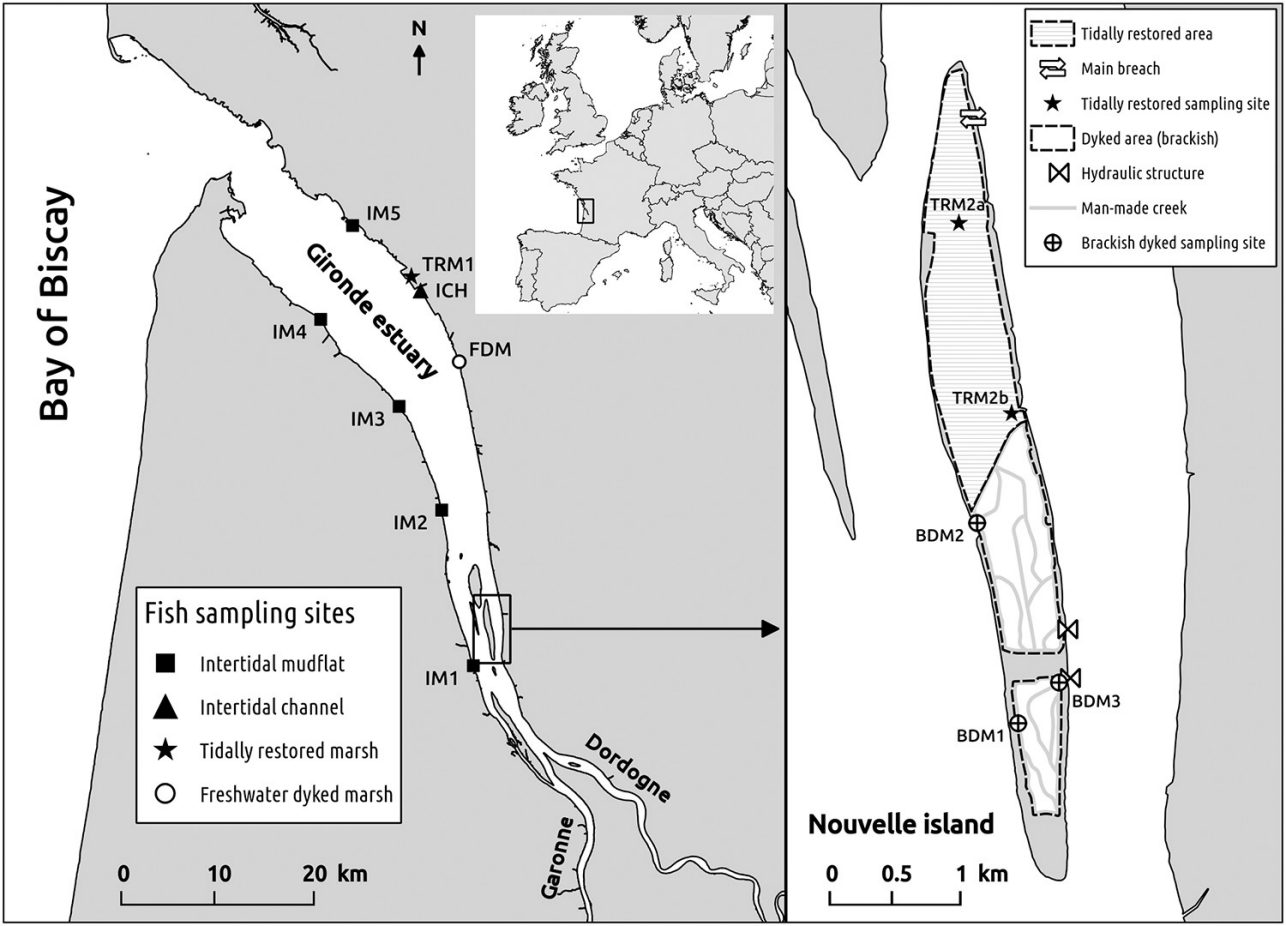
Location of the 13 fish sampling sites in the Gironde estuary. FDM, BDM1, BDM2 and BDM3 are dyked sampling sites. IM1, IM2, IM3, IM4, IM5 and ICH are natural sampling sites. TRM1 is the sampling site of the 12-year-old tidally restored Mortagne marsh. TRM2a and TRM2b are located in the 1-year-old restored part of the Nouvelle island.

Teleost fish were sampled on a set of 13 fish sampling sites in the Gironde estuary between 2011 and 2012 (Fig 2 and S1 Table). The sampling sites included three sites (TRM1, TRM2a, TRM2b) distributed between two storm-breached marshes, tidally restored one (TRM2) and 12 years ago (TRM1). The remainder of the sites consisted of six natural intertidal sites (five tidal flats, IM1 to IM5, and one intertidal channel, ICH), three brackish dyked marshes (BDM1 to BDM3) and one freshwater dyked marsh (FDM). The intertidal sites (natural and restored) were spread along the longitudinal gradient (i.e. salinity gradient) of the Gironde estuary. The dyked marshes consisted of man-made creeks, the water level of which was managed through one or several hydraulic structures. The brackish dyked marshes were managed with regular exchanges with the adjoining estuary whereas the freshwater dyked marsh was exclusively supplied with freshwater inputs from terrestrial streams.

Fish samplings were repeated five times (May 2011, Sep.-Nov. 2011, Feb.-Mar. 2012, May-June 2012 and July 2012) using the same fishing protocol. Two types of double fyke nets were used to collect fish, named hereafter ‘standard-fyke nets’ and ‘4 mm-fyke nets’. The two types of fyke nets had the same dimensions but differed in mesh size. The standard-fyke nets had a heterogeneous mesh size ranging from 17 mm at the entrance of the traps to 8 mm at the cod end and were used to sample mainly medium- and larger-sized fish. The 4 mm-fyke nets had a homogeneous mesh size of 4 mm and were designed to capture mainly smaller- and medium-sized fish.

Two 4 mm- and one standard-fyke nets were deployed simultaneously on each site and for each sampling time. Due to space limitation, only one 4 mm-fyke net was set for four of the five stations on the Nouvelle island (BDM1, BDM2, BDM3 and TRM2b). The fyke nets were set for 6 hours (from mid flow to mid ebb-tide) during daytime, on the mainland sites. Due to access constraints, the five stations on the Nouvelle island were sampled for an approximate duration of 24h.

For a given site and sampling time, the samples collected by each trap of a type of double fyke net were considered as replicates (*n* = 2 for the standard-fyke nets and *n* = 2 − 4 for the 4 mm-fyke nets). Fish were identified, counted and weighed at the species level. Length was measured on a subset of 50 individuals per species and sample. Smaller-sized fish (< 60 mm) were subsampled and collected for laboratory taxonomic identification. The other individuals were measured in the field and put back alive into the water. Fish species were subdivided *a posteriori* into several ecophases based on individual length to account for ontogenic changes in the functional niche of species. As several functional traits considered in this study were directly derived from fish diet, the splitting of species into ecophases was based on significant changes in diet with fish size (or age) reported in published studies (Section 1 in S1 Appendix). Ecophase numbers were estimated using the length-frequency distributions (LFDs) of the corresponding species.

Biomass per ecophase was estimated from the LFDs using specific species’ length-weight relationships. Catches were expressed as mean number of individuals per hour per double fyke net (CPUE, catches per unit effort) and mean wet weight per hour per double fyke net (BPUE, biomass per unit effort). For each type of double fyke nets, CPUE and BPUE were averaged across replicate samples at each *site* × *sampling time*. The cases where a type of double fyke nets outperformed the other were counted for every ecophase based on the average CPUE values at each *site* × *sampling time*. For each ecophase, only the abundance (CPUE and BPUE) of the overall best performing type of fyke net was retained to produce a unified fish ecophase assemblage at each *site* × *sampling time*. Consequently, in total, 64 fish communities were then described.

### Functional structure of fish communities

A single-trait approach to functional structure was retained because opposing patterns in single-trait distributions cannot be disentangled with multiple-trait indices [11, 14]. The functional structure of communities is commonly described through up to eight dimensions [4]: identity, richness, evenness, divergence, entropy, dispersion, specialization and originality. Functional identity refers to the traits’ mean values among individuals within a community [3]. Functional richness measures the range of trait values within a community and indicates the extent of resources used [59]. Unlike the other components of functional structure, functional richness is inherently independent of the abundance distribution among individuals or species. Functional evenness measures the propensity of dominant species to be functionally similar [5]. More specifically, it measures the regularity of spacing between individuals in the functional space occupied by a community. Functional divergence measures the tendency of individuals to aggregate on the outer margin of the functional space occupied by a community, regardless of its volume [59, 60]. Functional entropy [61, 62] and dispersion [63] are neighbour concepts that relate to the average functional distance of individuals from the other members of the community. As entropy and dispersion indices are tightly bound by a squaring factor [64], the two dimensions are highly correlated and only functional dispersion was used in this study. The functional specialization of a community measures the propensity of individuals in the community to have extreme trait values when compared to a pool of species larger than the community. Functional originality quantifies the average isolation level (in the functional space) of indivuals from their nearest neighbours in the community. Functional originality is the opposite concept of functional redundancy [27].

Functional identity was described by the community-weighted mean (*CWM*) of each functional trait [3]. Trait range (*Tr*; [59]) and functional regularity index (*FRO*; [65]) were chosen as indices of functional richness and evenness, respectively. The index of functional divergence (*FDvar*; [66]) was previously shown to be correlated with *Tr* [67] and suffers from several other limitations [67, 68]. Consequently, functional divergence was not used in this study. The index quantifying functional dispersion (*FDis*) encompasses the richness and divergence components of functional structure. The index of functional specialization, *wFSpe*, measured the average abundance-weighted distance of the species (or ecophases) of a community from the non-abundance-weighted barycenter of the species pool [5, 69]. In this study, the pool was defined as the whole set of fish ecophases collected across sites and sampling times. Functional originality was measured using the abundance-weighted mean nearest-neighbour distance (*wMNND*; [69, 70]. The formulas of the functional structure indices are extensively supplied in Table 1.

**Table 1:** The single-trait indices of functional structure.

Dimension	Index	Formula	References
Functional identity	Community-weighted mean (*CWM*)	$CWM=\sum_{i=1}^{S}p_{i}\times t_{i}$	[3]
Functional richness	Trait range (*Tr*)	*Tr* = *max*(*t*_*i*_) − *min*(*t*_*i*_)	[59]
Functional evenness	Functional regularity index (*FRO*)	$FRO=\sum_{i^{\prime }=1}^{S-1}min(PEW_{i^{\prime },i^{\prime }+1},\frac{1}{S-1})$ with: $PEW_{i^{\prime },i^{\prime }+1}=\frac{EW_{i^{\prime },i^{\prime }+1}}{\sum_{i^{\prime }=1}^{S-1}EW_{i^{\prime },i^{\prime }+1}}$ and: $EW_{i^{\prime },i^{\prime }+1}=\frac{\left|t_{i^{\prime }+1}-t_{i^{\prime }}\right|}{p_{i^{\prime }+1}+p_{i^{\prime }}}$	[65]
Functional dispersion	Functional dispersion (*FDis*)	$FDis=\sum_{i=1}^{S}p_{i}\times \left|t_{i}-CWM\right|$	[63]
Functional originality	Abundance-weighted mean nearest-neighbour distance (*wMNND*; *S* ≥ 3)	$\begin{array}{l} wMNND=p_{i^{\prime }=1}\times \left(t_{i^{\prime }=2}-t_{i^{\prime }=1}\right)\\ +\sum_{i^{\prime }=2}^{S-1}p_{i^{\prime }}\times min\left(t_{i^{\prime }}-t_{i^{\prime }-1},t_{i^{\prime }+1}-t_{i^{\prime }}\right)\\ +p_{i^{\prime }=S}\times \left(t_{i^{\prime }=S}-t_{i^{\prime }=S-1}\right) \end{array}$	[69, 70]
Functional specialization	Abundance-weighted distance to the baryleft of the species pool (*wFSpe*)	$wFSpe=\sum_{i=1}^{S}p_{i}\times \left|t_{i}-t_{pool}\right|$	[5, 69]

*S*: total number of ecophases (or species) in the community. *t*_*i*_: value of functional trait *t* for ecophase *i*. *p*_*i*_: relative abundance of ecophase *i* in the community ($\sum_{i=1}^{S}p_{i}=1$). *i*′: index of ecophases ranked by increasing trait values. *t*_*pool*_: non-abundance-weighted mean trait value of a pool of ecophases larger than the community.

Biomass data (BPUE) rather than counts (CPUE) were used for the computation of all abundance-based functional structure indices. Biomass values were fourth-root transformed to make sure that a small number of large fish did not have undue influence on the analyses [71]. The strength and the significance of the correlation between the functional structure indices and the naturalness of intertidal habitats were assessed using Spearman correlation coefficient (*ρ*).

### The gradient of intertidal habitat naturalness

The taxonomic response of nekton to salt marsh restoration was previously investigated using gradients of tidal restriction [72]. Similarly, the 13 sampling sites of our study were grouped *a priori* according to a gradient reflecting increasing intertidal habitat naturalness. Five ordered classes were considered: (1), freshwater dyked marshes (FDYK; one site); (2), brackish dyked marshes (BDYK; three sites); (3), low-aged tidally restored marshes (TRYO; two sites); (4), middle age tidally restored marshes (TRMA; one site); (5), natural intertidal habitats (NINT; six sites). This naturalness gradient relies on four hypotheses regarding the functional structure (and assembly patterns) of fish communities: (i) dyked marshes are more distant from an entirely natural status than the current intertidal habitats of the Gironde estuary; (ii) freshwater dyked marshes (with no estuarine inputs) are more distant than the brackish dyked marshes (with regular estuarine inputs) from the functional structure of intertidal habitats; (iii) the functional structure is more similar between restored and natural intertidal habitats than between dyked marshes and natural intertidal habitats; (iv) the naturalness of tidally restored marshes increases with time.

The functional identity (*CWMs*) of the fish assemblages was ordinated in a reduced space using non-metric multidimensional scaling (NMDS) for a post-hoc comparison with the naturalness gradient of intertidal habitats. The grouping of assemblages with respect to their position along the naturalness gradient was displayed using convex envelopes. The NMDS was carried out from the dissimilarity matrix (1 − *S*_*Gower*_) where *S*_*Gower*_ is the Gower similarity matrix [73] computed from the *CWMs* for each *site* × *sampling session*. Unlike other similarity coefficients, Gower similarity can be used to measure the similarity between communities based on heterogeneous variables (here, functional traits with different distributional properties) without transforming the variables (e.g., equalizing variance across variables). The NMDS axes were rotated *a posteriori* so that the first axis best discriminated habitat type (i.e., position along the naturalness gradient). The NMDS analysis was carried out using R software [74] and the *metaMDS* function of the ‘vegan’ package.

### Functional characterization of the fish ecophases

The food spectrum of the fish ecophases was characterized from a literature review encompassing an extensive range of ecosystems and habitat types where the species occur [25]. The food spectrum was described through the relative importance of eight food categories. Each food category was treated as a three-level ordinal variable: *P*1, primary importance; *P*2, secondary importance; *Ab*, incidental importance or absence from the diet. A principal coordinates analysis (PCoA) was carried out from the ecophases’ food spectrum matrix to reduce the dimensionality of the data set and to transform the ordinal variables into approximately continuous variables. The first three components of the PCoA (*DietAxis*1, *DietAxis*2, *DietAxis*3) were retained and considered as functional traits (Section 2 in S1 Appendix). The PCoA was carried out on R software [74] using the function *pcoa* of the ‘ape’ package.

**Fig 3:**
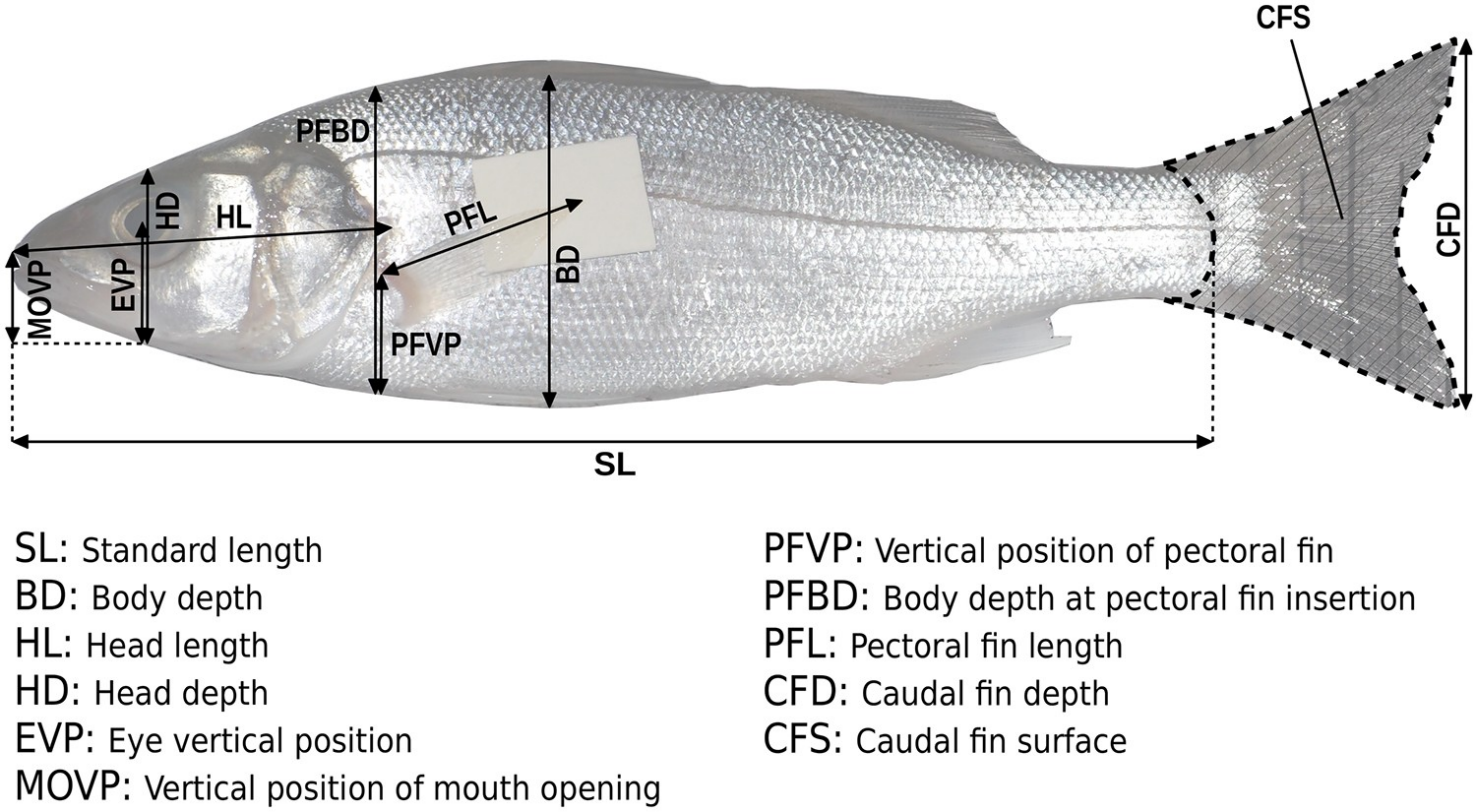
The morphological measurements on the fish ecophases.

The three diet-related functional traits were supplemented with seven ecomorphological traits related to food acquisition, swimming capacity and behaviour. The ecomorphological traits were measured on a set of 283 individuals belonging to the 62 sampled ecophases. Median number of measured individuals per ecophase was 4 (*min* = 1; *max* = 17). Most of the fish were collected *a posteriori* from the Gironde estuary or from nearby marine or continental hydrosystems. Morphological characteristics (Fig 3) were measured in the laboratory on fresh individuals.

Two additional ecomorphological traits were initially measured but removed so that Spearman correlation coefficient (*ρ*) between any pair of functional traits did not exceed 0.60 (Sections 3 and 4 in S1 Appendix). The 10 retained functional traits covered two axes of the fish functional niche, i.e. food acquisition (including diet) and swimming behaviour (Table 2). The whole set of functional traits were dimensionless and treated as continuous variables. Trait values were neither centered nor scaled prior to the computation of the functional structure indices.

**Table 2:** The 10 selected functional traits and their ecological interpretation.

Niche axis	Functional trait	Abbreviation	Computation	Ecological performance	References
Diet and food acquisition	Relative head length	*HLpSL*	$\frac{HL}{SL}$	Succion capacity (+) Filtration capacity (+) Maximum size of ingested animal prey (+) Share of plants and detritus in the diet (−)	[75–77]
	Vertical position of mouth opening	*MOVPpHD*	$\frac{MOVP}{HD}$	Vertical position of food in the water column (+)	[78, 79]
	Axis 1 of food spectrum	*DietAxis1*	*PCoA, axis 1*	Micro-sized animal prey (microinvertebrates) (+) Medium- and larger-sized animal prey (−)	This study (Section 2 in S1 Appendix)
	Axis 2 of food spectrum	*DietAxis2*	*PCoA, axis 2*	Vegetal and detrital food (−) Soft-bodied animal prey (+)	This study (Section 2 in S1 Appendix)
	Axis 3 of food spectrum	*DietAxis3*	*PCoA, axis 3*	Larger-sized mobile prey (+) Low-mobility, medium-sized benthic prey (−)	This study (Section 2 in S1 Appendix)
Swimming capacity and passive behaviour	Vertical position of the eye	*EVPpHD*	$\frac{EVP}{HD}$	Vertical position of the fish in the water column (−) Amount of swimming (mobile *vs*. sedentary behaviour) (−)	[75]
	Relative length of the pectoral fin	*PFLpSL*	$\frac{PFL}{SL}$	Current velocity in the habitat (−) Maneuvering capacity (+)	[75]
	Vertical insertion of the pectoral fin	*PFVPpPFBD*	$\frac{PFVP}{PFBD}$	Turning capacity (+)	[80] *in* [5]; [79]
	Relative depth of the caudal fin	*CFDpBD*	$\frac{CFD}{BD}$	Energetic cost of sustained swimming (−)	[75]
	Aspect ratio of the caudal fin	*sqCFDpCFS*	$\frac{CFD^{2}}{CFS}$	Energetic cost of sustained swimming (−) Acceleration capacity (−) Maneuvering capacity (−)	[75, 78, 81, 82]

An increase in the functional trait value affects positively (+) or negatively (−) a type of ecological performance.

### Factoring out covarying abiotic factors

Our main aim was to assess the change in functional structure and patterns of community assembly with increasing naturalness of intertidal habitats. Therefore, the covarying abiotic variables that were not intrinsically linked with a change in naturalness were treated as confusing factors. In our sampling strategy, all sites were evenly sampled at different periods of the year, downplaying the potential bias of season. Water temperature and salinity were measured at the beginning and the end of each sampling time and for each site. Water temperature did not differ significantly between the five types of habitat spread along the naturalness gradient (Kruskal-Wallis one-way analysis of variance; *p* = 0.12).

Contrastingly, significant differences in salinity values were observed among types of habitat (Kruskal-Wallis one-way analysis of variance; *p* < 0.001). In addition, a strong monotonic correlation was found between salinity and the naturalness gradient (*ρ* = 0.63; *p* < 0.001). Collinearity between salinity and intertidal naturalness was considered a bias resulting from our sampling strategy. Consequently, we used semipartial correlation [83]. Computing the semipartial correlation of each functional index with intertidal naturalness given salinity measured a pure effect of increasing naturalness on the functional structure of communities. The ‘ppcor’ package [83] in R software was used to compute Spearman semipartial coefficient (*ρ*_*sp*_) and the corresponding *p*-value. The magnitude of the salinity effect was highlighted by substracting the raw correlation coefficients (i.e. without partialling out salinity) to the semipartial correlation coefficients.

The functional response of fish communities to increasing naturalness was displayed using principal component analysis (PCA) from the semi-partial correlation matrix (*ρ*_*sp*_) of the functional indices other than functional identity (i.e., functional richness, evenness, dispersion, specialization and originality). Unsupervised classification was performed based on the same *ρ*_*sp*_ matrix using Euclidean distance and Ward’s method. Euclidean distance was chosen because the original variables (i.e. the semi-partial correlation coefficients associated with the different types of functional indices) were continuous variables with the same scale of measurement. Data (*ρ*_*sp*_) were neither centered nor scaled prior to the PCA nor the classification analysis.

### Community assembly patterns along the naturalness gradient

Three types of null models were used to identify the trait-based assembly processes operating at different spatial scales. The first null model (NM1) was the trial swap null model [84]. The NM1 randomly reallocated ecophases presence-absence within the community matrix while keeping the marginal sums constant. This was equivalent to randomly draw, for each community, a fixed number of ecophases (equal to the actually observed number) from the total pool sampled across the 13 sites while controlling for the occurrence of the ecophases across communities. A common criticism to the NM1 is that drawing from an ‘extended pool’ generates overly diverse communities comprised of ecophases that are unlikely to co-occur in real communities [19, 85]. Most obviously, pooling ecophases across communities in our data set overrode the physiological and evolutionary boundaries between freshwater and saline ecosystems.

To overcome the shortcomings of the NM1, we developed a second null model (NM2) that allowed communities to be assembled from a ‘reduced pool’ of ecophases accounting for the species’ tolerance to salinity. Fish species tolerance to salinity was described from an external fish database used to assess the ecological status of french estuaries [86, 87]. Species tolerance to salinity was characterized from the species’ frequency of occurrence (FO) among four predefined salinity classes (see S2 Appendix for details). In the NM2, the ecophases of a community were randomly replaced by an equal number of ecophases able to cope with the salinity measured during the sampling event. In order to account for spatial mass effects [88], ecophases occurring at salinities remote from their putative preferenda were randomly replaced by an equal number of ecophases sharing the same pattern of salinity tolerance. Although the number of ecophases was kept constant between observed and simulated communities, the NM2 (unlike NM1) did not control for the number of ecophases’ occurrences across simulated communities. Compared with the NM1, the NM2 was intended to dampen the effects of dispersal limitations and strong abiotic gradients (salinity) and to focus on smaller-scale environmental filtering [19]. Functional richness (*Tr*) was computed from the communities simulated under the NM1 and the NM2.

The null model 3 (NM3) randomly reallocated relative abundances among co-occurring ecophases within observed communities [12, 29]. Functional dispersion (*FDis*) was computed for each community simulated under the NM3.

For each null model, 9 999 communities were simulated. We computed the effect size (ES) of an index as the proportion of the simulated values that were lower than the actual value ([14]; see S3 Appendix for exact calculation). ES values were scaled to vary between -1 and 1. Values close to 0 matched the null expectation. The retained ES was preferred to the standardized effect size (SES; [89]) because the latter is measured in units of standard deviation from the mean and relies on a normal distribution of simulated values after centering and scaling, an assumption that is not always met (e.g., [14]).

The effect size of *Tr* (*TrES*) under the NM1 and the NM2 transformed *Tr* into a pure measure of functional richness [90], controlling for the effect of ecophases’ richness [9]. Likewise, the effect size of *FDis* (*FDisES*) under the NM3 turned *FDis* into a pure measure of functional divergence [90]. Changes in patterns of community assembly along the naturalness gradient were investigated based on the correlation strength and significance between naturalness levels and the values of pure functional richness (*TrES*) and divergence (*FDisES*). In addition, assembly patterns were tested at three positions along the naturalness gradient, i.e. for dyked marshes, tidally restored marshes and natural intertidal habitats. The five initial groups of sites were reduced to three in order to limit site effects. At each position, two-tailed Wilcoxon signed-rank tests were performed to detect a significant deviation of *TrES* and *FDisES* from null expectations. Environmental filtering was evidenced where *TrES* was lower (i.e., trait range was narrower) than expected under the NM1 or the NM2. Higher-than-expected *FDisEs* (i.e., trait divergence) revealed limiting similarity processes whereas lower-than-expected *FDisES* (i.e., trait convergence) was indicative of local optimum trait values.

In this study, biomasses (BPUE) rather than numbers (CPUE) were used for the weighting of all abundance-based functional structure indices although [91] found that multiple-trait indices performed equally good irrespective of the type of abundance (biomasses or numbers) used for the weighting of the traits’ values.

## Results

In total, 200918 individuals were caught across all sites with the two types of double fyke nets, corresponding to 62 ecophases and 34 fish species (Section 1 in S1 Appendix). In the dyked marshes, the species *Pseudorasbora parva* and *Gasterosteus aculeatus* made up 51.1% and 41.8% of the total fish numbers, respectively. The biomass was dominated by *Anguilla anguilla*, *Gasterosteus aculeatus* and *Cyprinus carpio* (36.3%, 17.6% and 17.6%, respectively). In the tidally restored marshes, *Pomatoschistus microps* (72.9%), *Liza ramada* (12.6%) and *Pomatoschistus minutus* (8.3%) were the most numerous species. *L. ramada* dominated biomass (76.9%), followed by *A. Anguilla* (11.4%). In the natural intertidal habitats, *P. microps* was by the far the most important contributor in terms of numbers (96.0%), whereas biomasses were more evenly spread among seven species: *P. microps* (28.3%), *A. anguilla* (15.9%), *L. ramada* (13.2%), *Solea solea* (12.2%), *Platichthys flesus* (11.4%), *Dicentrarchus labrax* (9.2%) and *Argyrosomus regius* (5.5%).

### Functional identity along the gradient of intertidal habitat naturalness

**Fig 4:**
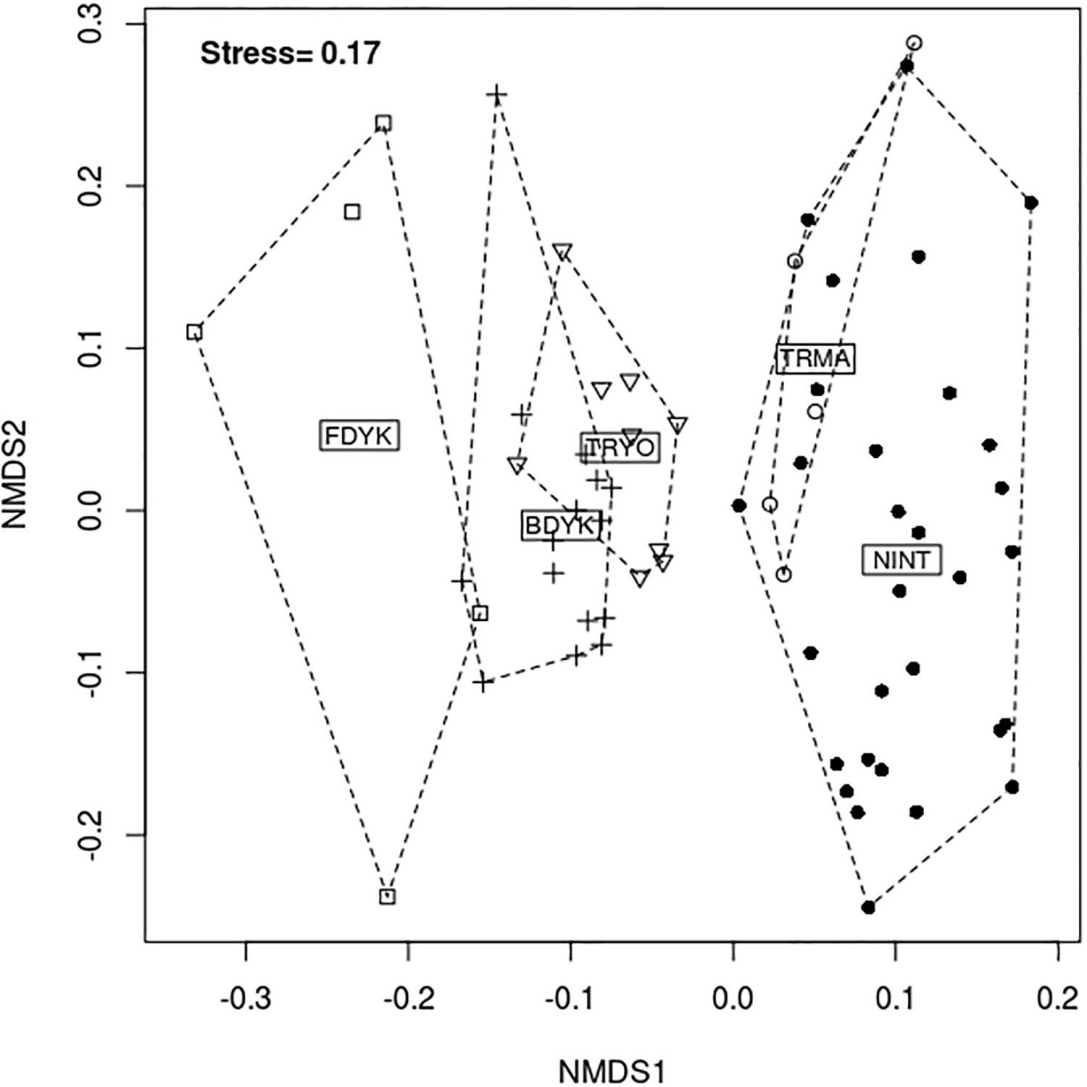
Two-dimensional non-metric multidimensional scaling (2D-NMDS) of the fish community-weighted means (*CWMs*) among five groups of habitats clustered with respect to their naturalness. The points on the plot correspond to the communities at each *site* × *sampling time*. Abbreviations for habitat groups: FDYK, Freshwater dyked marshes (one site); BDYK, Brackish dyked marshes (three sites); TRYO, Young tidally restored marsh (two sites); TRMA, Middle age tidally restored marsh (one site); NINT, Natural intertidal habitats (six sites).

Ordination of the functional identity of fish communities in a 2D-NMDS supported the *a priori* ordering of habitat types along the gradient of intertidal habitat naturalness (Fig 4). Although the stress value was quite high (0.17), the 2D-ordination still provided a usable picture. A clear difference appeared along the first axis of the ordination between young (TRYO) and older (TRMA) restored marshes (i.e., non-overlapping convex envelopes).

### Change in functional structure with increasing naturalness

The functional identity (*CWM*) of six functional traits showed significant correlations with the naturalness gradient (Table 3). Semipartial correlation was strong (|*ρ*_*sp*_|≥ 0.60) for *sqCFDpCFS*, moderate (0.40 ≤ |*ρ*_*sp*_| < 0.60) for *EVPpHD*, *DietAxis*2 and *DietAxis*3 and weak (0.20 ≤ |*ρ*_*sp*_| < 0.40) for *PFVPpPFBD* and *DietAxis*1. The significant correlations in the *CWMs* of *HLpSL* and *MOVPpHD* were no longer detected after salinity was partialled out. Conversely, the significant negative correlation for the *CWM* of *DietAxis*3 only appeared when the effect of salinity was controlled for.

**Table 3:** Monotonic response of the functional structure of fish communities to increasing intertidal naturalness after factoring out salinity.

	Index	*HLpSL*	*MOVPpHD*	*DietAxis1*	*DietAxis2*	*DietAxis3*	*CFDpBD*	*sqCFDpCFS*	*PFLpSL*	*PFVPpPFBD*	*EVPpHD*
Functional identity	*CWM*	+0.09	−0.24	+0.31*	+0.50***	−0.40**	−0.09	−0.60***	+0.03	+0.37**	+0.53***
		(−0.22)	(+0.19)	(+0.04)	(−0.11)	(−0.33)	(−0.08)	(+0.27)	(−0.05)	(−0.03)	(−0.27)
Functional richness	*Tr*	−0.22	+0.28*	+0.26*	−0.18	+0.25	−0.11	+0.04	+0.11	+0.43***	+0.40**
		(−0.03)	(−0.18)	(−0.02)	(−0.011)	(−0.37)	(0.13)	(−0.04)	(−0.02)	(−0.27)	(−0.23)
Functional evenness	*FRO*	−0.23	−0.43***	−0.22	−0.40**	−0.24	+0.15	+0.03	+0.25	−0.08	−0.26*
		(+0.14)	(+0.12)	(+0.29)	(+0.19)	(+0.03)	(+0.06)	(+0.06)	(−0.08)	(+0.10)	(+0.15)
Functional dispersion	*FDis*	−0.17	+0.34**	+0.60***	−0.28*	+0.27*	−0.21	+0.17	−0.03	+0.42***	+0.31*
		(+0.21)	(−0.20)	(−0.10)	(+0.17)	(−0.32)	(+0.23)	(+0.04)	(+0.17)	(−0.23)	(−0.28)
Functional originality	*wMNND*	−0.41**	−0.11	−0.24	−0.26*	−0.02	+0.03	−0.17	+0.22	+0.007	−0.20
		(+0.13)	(−0.008)	(+0.013)	(+0.08)	(−0.08)	(+0.07)	(+0.10)	(−0.03)	(+0.009)	(+0.003)
Functional specialization	*wFSpe*	−0.29*	+0.44***	+0.52***	−0.11	+0.41**	−0.20	+0.46***	+0.004	+0.69***	+0.60***
		(+0.23)	(−0.26)	(−0.30)	(+0.19)	(−0.24)	(+0.22)	(−0.13)	(+0.18)	(−0.18)	(−0.21)

Values correspond to the Spearman semipartial correlation coefficient (*ρ*_*sp*_) computed between a single-trait functional index and the naturalness level of intertidal habitats (pairs of values correspond to the fish communities at each *site*×*sampling time*). The effect of salinity was partialled out. The types of functional indices are displayed in rows and the functional traits are displayed in columns. Positive values indicate an increase with the naturalness gradient and negative values indicate a decrease. Numbers in brackets are the arithmetic differences between Spearman semi-partial (*ρ*_*sp*_) and raw (*ρ*) correlation coefficients. Significance values associated with *ρ*_*sp*_:

*, 0.01 < *p* ≤ 0.05;

**, 0.001 < *p* ≤ 0.01;

***, *p* ≤ 0.001.

**Fig 5:**
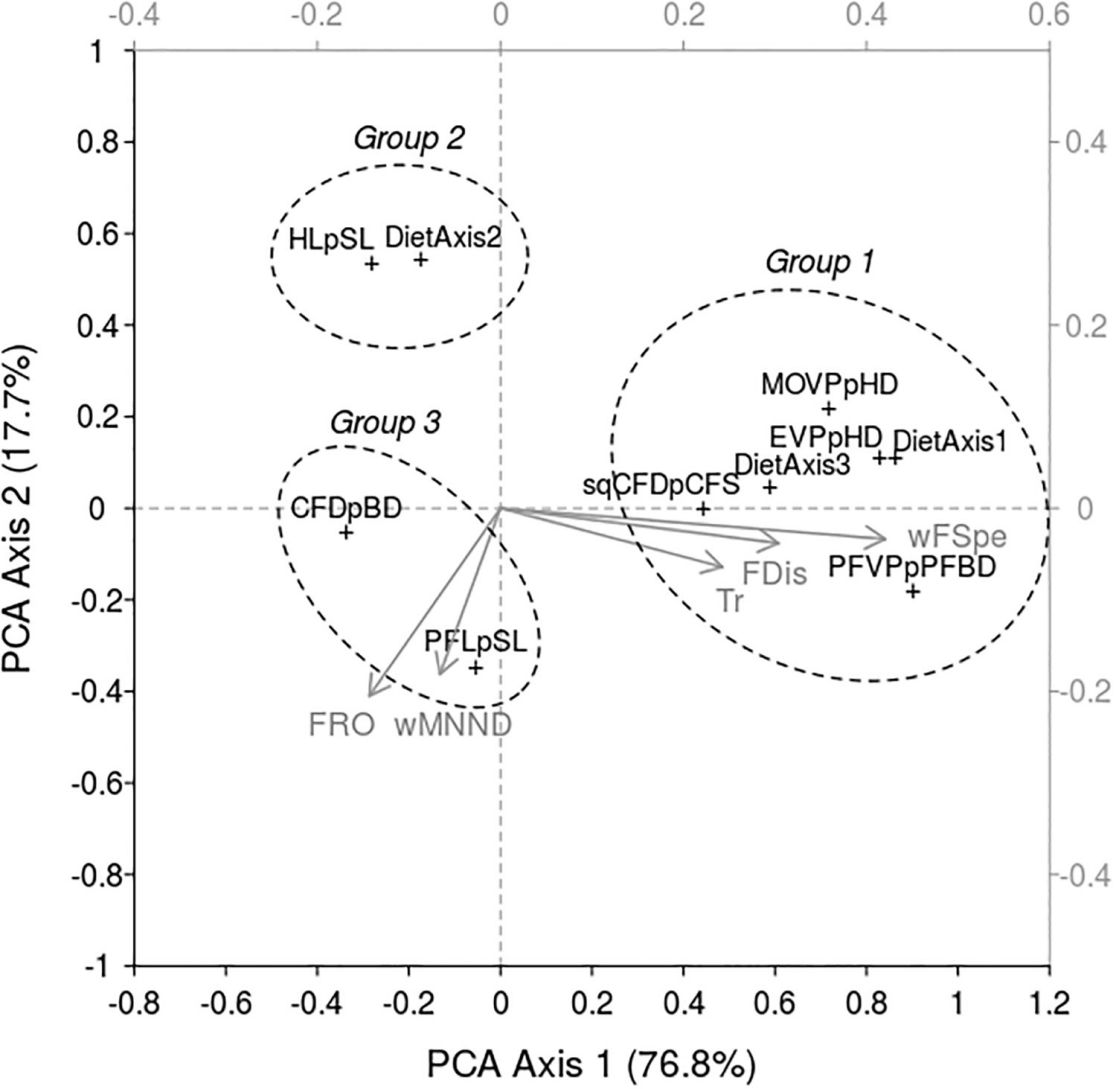
Principal component analysis (PCA) biplot of the response of functional structure indices (other than functional identity) to increasing naturalness of intertidal habitats. The response of functional structure indices was measured using Spearman semipartial correlation (*ρ*_*sp*_). The top and right axes (grey color) show the loadings of the variables. Ellipses correspond to the groups of functional traits identified from the clustering analysis (Fig 6). See Table 2 for the abbreviations of functional traits. Codes of functional indices: *Tr*, functional richness; *FRO*, functional evenness; *FDis*, functional dispersion; *wMNND*, functional originality; *wFSpe*, functional specialization.

Indices related to functional richness (*Tr*), dispersion (*FDis*) and specialization (*wFSpe*) showed a coordinated response to increasing naturalness (Table 3 and Fig 5). Likewise, trends in evenness (*FRO*) and originality indices (*wMNND*) were negatively correlated with naturalness. The first component axis of the PCA captured most of the total variance (76.8%) and was mainly drawn by *wFSpe* and, to a lesser extent, by *FDis* and *Tr*. The second component axis (17.7% of total inertia) discriminated functional traits according to the response of *FRO* and *wMNND* to increasing naturalness. Compared with *wFSpe* and *Tr*, the orthogonal response of *FRO* and *wMNND* indicated that the latter indices captured a complementary dimension of the functional response of communities to increasing naturalness.

**Fig 6:**
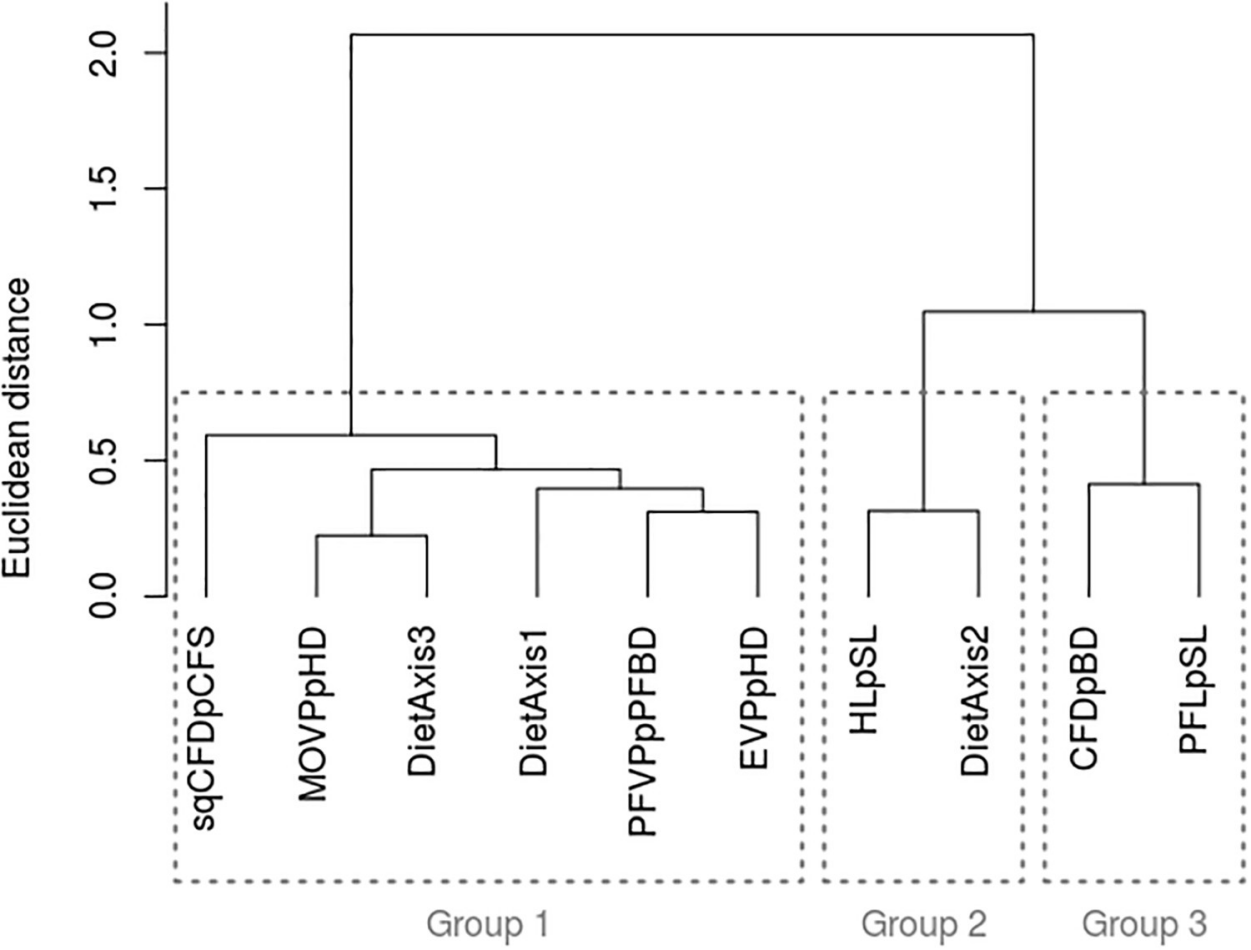
Clustering of the functional traits according to the response (Spearman semipartial correlation, *ρ*_*sp*_) of their functional structure indices (richness, evenness, dispersion, originality and specialization) to increasing naturalness of intertidal habitats. The names of the functional traits were in Table 2.

Three groups of functional traits were identified from the clustering of the traits responses (functional richness, evenness, dispersion, originality and specialization) to increasing naturalness (Fig 6). Group 1 includes traits related to the vertical dimension of the habitat (*EV PpHD* and *MOV PpHD*), the size and mobility of animal prey organisms (*DietAxis*1 and *DietAxis*3), the amount of swimming (*EV PpHD*, *sqCFDpCFS*) and the maneuvering capacity (*PFV PpPFBD*). Traits clustered in group 2 describe the relative importance of vegetal and detrital *vs*. animal components of the diet (*HLpSL* and *DietAxis*2). Group 3 relates to the swimming capacity (sustained swimming; *CFDpBD*) and the current velocity in the habitat (*PFLpSL*).

For traits of group 1, positive correlations with naturalness were substantiated for functional richness, dispersion and specialization. Functional specialization showed a moderate to strong (0.41 ≤ *ρ*_*sp*_ ≤ 0.69) significant positive correlation with naturalness; functional dispersion, a weak to strong positive correlation (0.17 ≤ *ρ*_*sp*_ ≤ 0.60); and functional richness, a weak to moderate positive correlation (0.04 ≤ *ρ*_*sp*_ ≤ 0.43).

In contrast, functional specialization (−0.29 ≤ *ρ*_*sp*_ ≤ 0.004), dispersion (−0.28 ≤ *ρ*_*sp*_ ≤ −0.03) and richness (−0.22 ≤ *ρ*_*sp*_ ≤ 0.11) did not show significant positive correlations with naturalness for traits belonging to groups 2 and 3. For traits of group 2, functional evenness (−0.23 ≤ *ρ*_*sp*_ ≤ −0.40) and originality (−0.26 ≤ *ρ*_*sp*_ ≤ −0.41) showed weak to moderate negative correlations. For traits of group 3, evenness (0.15 ≤ *ρ*_*sp*_ ≤ 0.25) and originality (0.03 ≤ *ρ*_*sp*_ ≤ 0.22) showed weak, non-significant positive correlations.

### Patterns of community assembly along the naturalness gradient

The effect sizes of *Tr* (*TrES*) under each of the NM1 and the NM2 showed significant correlations with naturalness for seven functional traits (Table 4). Under the NM1, four traits exhibited weak (0.20 < *ρ* ≤ 0.40; *MOV PpHD*), moderate (0.40 < *ρ* ≤ 0.60; *EV PpHD*, *DietAxis*3) or strong (*ρ* > 0.60; *PFV PpPFBD*) positive correlations. Three traits (*HLpSL*, *CFDpBD* and *DietAxis*2) showed moderate negative correlations (−0.60 ≤ *ρ* < 0.40). Compared to the NM1, no significant correlation was detected for *DietAxis*2 under the NM2 whereas a significant negative correlation was found for *PFLpSL*. In the subsequent analyses, we only considered the results of the NM2, which accounted for finer processes of environmental filtering.

**Table 4:** Effect sizes of functional richness (*TrES*) and dispersion (*FDisES*) along the gradient of intertidal habitat naturalness.

Index	Null model	*HLpSL*	*MOVPpHD*	*DietAxis1*	*DietAxis2*	*DietAxis3*	*CFDpBD*	*sqCFDpCFS*	*PFLpSL*	*PFVPpPFBD*	*EVPpHD*
*TrES*	NM1	−0.41***	+0.32	+0.18	−0.41***	+0.55***	−0.49***	−0.10	−0.02	+0.66***	+0.44***
*TrES*	NM2	−0.55***	+0.28*	+0.10	−0.20	+0.42***	−0.55***	−0.03	−0.32**	+0.64***	+0.32*
*FDisES*	NM3	−0.17	+0.35**	+0.41***	−0.04	+0.13	−0.25*	+0.30*	+0.17	+0.10	−0.23

Values are Spearman correlation coefficients. Positive values indicate an increase along the naturalness gradient and negative values indicate a decrease. Null model 1 (NM1) was the traditional matrix swap null model. Null model 2 (NM2) reshuffled ecophase presence-absence with respect to the species’ tolerance to salinity. Null model 3 (NM3) randomly reallocated abundances among ecophases within observed communities (see Material and methods for further details). Significance values:

*, 0.01 < *p* ≤ 0.05;

**, 0.001 < *p* ≤ 0.01;

***, *p* ≤ 0.001.

Significant correlations between *FDisES* and naturalness were detected for four functional traits: three traits (*DietAxis*1, *MOV PpHD* and *sqCFDpCFS*) showed positive correlations, reflecting increased divergence, or decreased convergence, with naturalness; and one trait (*CFDpBD*) showed a negative correlation. Correlation strength was weak (*MOV PpHD*, *sqCFDpCFS*, *CFDpBD*) to moderate (*DietAxis*1). Regarding *DietAxis*2, no significant correlation with naturalness was detected for *TrES* nor for *FDisES*. Significant correlations for both *TrES* and *FDisES* were found for *MOV PpHD* and *CFDpBD*.

**Fig 7:**
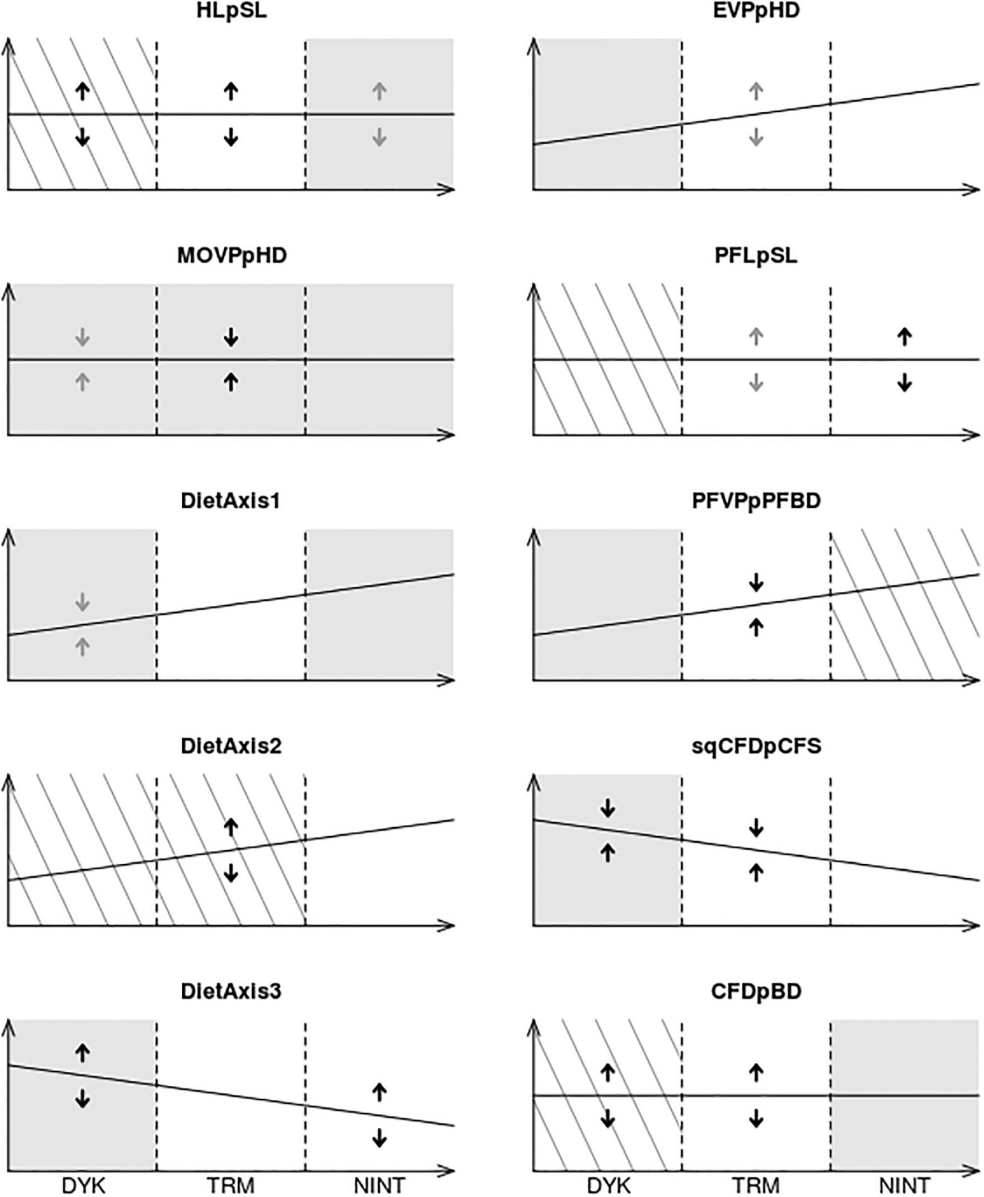
Patterns of fish community assembly and monotonic trends in community-weighted means (*CWMs*) along the naturalness gradient of intertidal habitats. The X-axis represents the gradient of intertidal habitat naturalness (DYK, dyked marshes; TRM, tidally restored marshes; NINT, natural intertidal habitats) and the Y-axis represents the *CWM*. The names of the functional traits were in Table 2. Trends related to the *CWMs* were corrected for salinity using Spearman semipartial correlation. Ascending and descending straight lines represent, respectively, significant positive and negative correlation between the *CWMs* and intertidal naturalness. Horizontal straight lines indicate lack of significant correlation. Grey areas correspond to parts of the gradient where environmental filtering (i.e., lower-than-expected trait range) was detected under the null model 2. Striped areas indicate higher-than-expected trait range. Double arrows indicate either trait convergence (convergent arrows) or divergence (divergent arrows). Grey arrows indicate both lower significavity (0.01 < *p* ≤ 0.05) and lower effect sizes (−0.5 < *medianES* < 0.5). Adapted from [14].

Patterns of community assembly (environmental filtering, trait convergence, trait divergence) at three positions along the naturalness gradient are summarized in Fig 7 (see also S1 Fig for the results with the NM1). Under the NM2, the number of traits for which environmental filtering (i.e. lower-than-expected *TrES*) was detected decreased from six in dyked marshes to four in natural intertidal habitats, with the minimum number (one trait) recorded in tidally restored marshes. Environmental filtering was detected for *MOV PpHD* throughout the naturalness gradient. With increasing naturalness, environmental filtering appeared for *HLpSL* and *CFDpBD* and disappeared for *EV PpHD*, *DietAxis*3 and *PFV PpPFBD*. Under the NM1, environmental filtering was detected for a higher number of trait (three) in the tidally restored marshes. Contrary to the NM2, environmental filtering appeared for *DietAxis*2 with increasing naturalness and disappeared for *MOV PpHD* and *DietAxis*1.

Trait convergence (i.e. lower-than-expected *FDisES*) disappeared with increasing naturalness. In fact, the number of underdispersed traits dropped from three in dyked marshes (*MOV PpHD*, *DietAxis*1, *sqCFDpCFS*) and in restored marshes (*MOV PpHD*, *sqCFDpCFS*, *PFV PpPFBD*) to none in natural intertidal habitats.

Trait divergence (i.e. higher-than-expected *FDisES*) was evidenced all along the naturalness gradient, with an equal number of three overdispersed traits in dyked and natural habitats and a maximum number of five overdispersed traits in the tidally restored marshes. Trait divergence appeared for *PFLpSL*, disappeared for *CFDpBD* and persisted throughout the naturalness gradient for *HLpSL*.

## Discussion

In our study, we used a naturalness gradient encompassing artificialized, restored and natural sites as a surrogate for restoration time to identify patterns in community change during restoration. Significant changes in the functional structure and the patterns of fish community assembly were identified with increasing naturalness of intertidal habitats.

### Changes in functional structure

Significant correlations between functional indices and naturalness gradient highlighted that changes in functional structure occurred along the naturalness gradient. Those changes were mainly trait-dependent accrediting the single-trait approach recommended elsewhere [11].

Functional identity, specialization and dispersion were the dimensions of the functional structure that most responded to increasing naturalness. The functional identity of communities was previously shown to change along environmental gradients [11, 14] and ecological trajectories such as habitat degradation [71] or habitat restoration [7]. In our study, the significant trends in the community-weighted means (*CWMs*) of most traits along the naturalness gradient were associated with increasing functional specialization and, to a lesser extent, with increasing functional dispersion. The changes in *CWMs* resulted more from the increasing abundances of specialized ecophases within communities than from changes in trait range (i.e., functional richness). Indeed, although functional specialization, dispersion and richness showed a coordinated response to increasing naturalness, the responses of functional specialization and dispersion were of higher amplitude.

Six of the seven significant monotonic changes in functional specialization were increasing trends, reflecting an overall increase in specialization along the naturalness gradient. For most traits, the increase in functional specialization was driven by ecophases of gobies (*Pomatoschistus microps*, *P. minutus*) and soles (*Solea solea*, *S. senegalensis*). The older ecophases of *Dicentrarchus labrax* and *Argyrosomus regius* were responsible for the increased specialization of the trait related to prey mobility and vertical position. The three ecophases of *Platichthys flesus* contributed to the increased specialization of traits related to the vertical use of the habitat.

Specialization increased for traits associated with various dimensions of the ecophases functional niche (e.g., the vertical use of the habitat or the size and mobility of animal prey) meaning that restored and natural intertidal habitats supplied fish with several specific foraging and dwelling conditions absent from the dyked marshes. Consistently, tidal restriction was shown to result in the biotic homogenization of marshlands as fish assemblages of dyked marshes were mostly comprised of generalist species (i.e., with common trait values; [43]). On a larger scale, the functional specialization in locomotion and food acquisition decreased in fish communities following habitat degradation in a tropical estuary [5]. Conversely, we showed that the share of fish with specialized food and habitat requirements raised with increasing intertidal habitat naturalness. This finding is in contrast with the common statement that estuarine communities are mostly comprised of generalist species [39]. This apparent contradiction possibly results from inconsistent references for the comparison. In fact, whereas estuarine fish species may be described as generalists when compared to marine (non-estuarine) species, intertidal species may still be more specialized than the ones dwelling in dyked marshes.

In our study, the four significant changes in functional richness along the naturalness gradient were increasing trends further substantiating the broader availability of ecological resources. Collectively, those results were consistent with our first hypothesis, i.e., that functional richness and specialization increase in the course of tidal marsh restoration, although the significant trends concerned a limited subset of the functional traits.

The few significant trends detected in functional evenness (three traits) and originality (two traits) were decreasing trends, meaning that the dominating ecophases tended to be more similar with increasing naturalness. Consistently, woody plant species of a subtropical forest became more similar to each other with successional time [9]. High functional redundancy (i.e. low functional originality) is considered a desirable property of ecosystems, granting them higher stability or resiliency [25, 27, 33].

### Patterns of community assembly

Comparing actual fish communities with communities assembled under null models revealed ecological determinism in community assembly along the naturalness gradient of intertidal habitats. In our study, the effect sizes of two functional diversity indices (i.e., *Tr* for functional richness and *FDis* for functional dispersion) were used to detect assembly patterns at three positions along the naturalness gradient: (i) trait range narrowing (i.e. environmental filtering) was evidenced by lower-than-expected *Tr* (i.e. significantly negative *TrES*); (ii) trait divergence (i.e. limiting similarity), by higer-than-expected functional dispersion (i.e. significantly positive *FDisES*); and (iii) trait convergence, by lower-than-expected functional dispersion (i.e. significantly negative *FDisES*). In addition, significant correlations between *TrES* or *FDisES* and naturalness revealed monotonic changes in the strength of environmental filtering and in the functional dispersion effect of biotic interactions, respectively.

Environmental filtering, trait divergence and trait convergence all shaped the functional structure of fish communities at least at some points of the naturalness gradient. The rules governing functional structure were largely trait-dependent corroborating the assumption that contrasted assembly processes may operate simultaneously along different niche axes. Cases where trait divergence and trait convergence occurred simultaneously were found, as previously observed in terrestrial invertebrate communities [12].

Overall, environmental filtering had a moderately weaker effect on community assembly with increasing naturalness. More importantly, we evidenced a strong shift in the traits affected by environmental filtering between the two ends of the naturalness gradient. This implies that the abiotic drivers of fish ecophases’ exclusion were not the same between dyked and natural intertidal habitats. In dyked marshes, environmental filtering affected traits related to food acquisition and swimming ability whereas in natural and restored intertidal habitats, most of the traits affected were related to food acquisition.

Trait convergence (i.e. local optimum trait values) disappeared with increasing naturalness. The decreasing importance of trait convergence with increasing naturalness most likely resulted from an increased provision of suitable habitat and food resources (i.e., thoses resources were no longer limiting). The absence of trait convergence was interpreted as a relaxation of both biotic and abiotic constraints allowing the coexistence of a wider range of functional strategies [14].

Trait divergence (i.e. limiting similarity) was evidenced all along the naturalness gradient, with the maximum number of overdispersed traits in the tidally restored marshes. Consequently, the assumption that trait divergence increases with naturalness (as in ecological succession) was not met. In tidally restored marshes, the high number of overdispersed traits was possibly due to processes disrupting the trait-environment relationships such as habitat micro-heterogeneity [92] and historical contingency [90, 93, 94]. Indeed, the intertwining between the previous topographical and hydrographical features of the dyked marshlands and the dynamic hydromorphological processes caused by the return of the tide (i.e. sediment deposition, incision of a tidal channel network and reestablishment of halophytic vegetation) increases habitat heterogeneity in tidally restored sites [95]. In addition, incomplete drainage in the early stages of tidal restoration favours certainly the temporary persistence of pre-restoration fish species in the ponding areas, leading to historical contingency effects. Both historical contingency effects and habitat micro-heterogeneity may have promoted the (at least temporary) coexistence of functionally dissimilar ecophases in tidally restored marshes.

### Change in ecological functioning

#### Vertical use of the habitat

Environmental filtering disappeared and functional identity (*CWM*) significantly changed for the traits related to the vertical position of the fish in the water column and to the amount of low-mobility benthic prey in the diet, with increasing naturalness. In addition, the decrease in the *CWM* of the trait associated with the vertical position of food was only marginally non significant. Those combined results indicated that (1) dyked marshes were depleted in fish ecophases dwelling and feeding on or close to the bottom, and (2) the use of the water column was progressively extended towards the bottom with increasing naturalness. Several hypotheses potentially account for the avoidance of the bottom in dyked marshes including unsuitable sediment texture, harsh abiotic conditions (e.g., due to oxygen depletion) and low abundance of benthic prey [72]. In contrast, natural intertidal habitats (marshes, mudflats) provide fish with high amounts of benthic prey [96–98].

#### Types of food resources used

The increase in the *CWM* of the main trait related to the vegetal and detrital *vs*. animal components of the diet revealed a decreasing reliance on microphytes and living or dead plant materials with increasing naturalness of intertidal habitats. Two functional traits associated with animal prey size and mobility showed increased functional specialization with increasing naturalness, indicating higher specialization in animal food acquisition strategies. The increase in the *CWM* of a trait related to animal prey size reflected a growing intake of microinvertebrates. In addition, trait convergence was evidenced only in dyked marshes for this trait, meaning that microinvertebrates were potentially a limiting food resource in those habitats. The *CWM* of another trait reflecting animal prey size decreased with increasing naturalness indicating that the dominant ecophases fed more extensively on benthic non-mobile prey such as annelids and shelled molluscs, and neglected non-benthic mobile organisms (e.g., fish). This finding is in agreement with previous studies on the macrozoobenthic communities of created marshes, substantiating that large-bodied molluscs were more common within older systems and that the importance of polychaetes increased after 15 years [99]. In addition, lower incidence of piscivorous predators with increasing naturalness is consistent with the widely assumed nursery value of natural intertidal habitats [96, 100].

#### Swimming ability and behaviour

Three of the five morphological traits related to fish swimming ability and behaviour showed monotonic trends in functional identity and increasing trends in functional specialization. The inclusion of specialized morphologies and swimming behaviours probably accounted for the shifts in *CWMs* in the increasingly natural habitats. With increasing naturalness, fish assemblages were comprised of a growing proportion of ecophases with sedentary lifestyles, living closer to the bottom or more adapted for maneuvering and burst swimming than for sustained swimming. Environmental filtering affected swimming traits in the dyked marshes, excluding ecophases with sedentary lifestyles and high maneuvering capacity. Contrastingly, the restored and natural intertidal habitats did not filter out fish based on their swimming behaviour. Overall, free-swimming strategists (i.e. endurance/cruising specialists) tended to be increasingly accompanied by swimming generalists and shelter-seeking strategists with increasing naturalness of intertidal habitats.

### Partialling out of salinity

As freshwater and marine fish species are subjected to different selective pressures and dispersal constraints [101] resulting in specific traits values and combinations [34, 102], partialling out salinity is an important step when comparing the functional structure of fish communities originating from freshwater, brackish and marine ecosystems. We found that disentangling the effects of salinity and naturalness revealed, or discarded, significant trends in the functional structure indices, depending on the trait under scrutiny. After controlling for the effect of salinity, environmental filtering was no longer detected in natural intertidal habitats for the trait related to the vegetal and detrital *vs*. animal components of the diet meaning that the depletion in ecophases feeding on plants and detritus resulted from the lower share of omnivorous and herbivorous species among the higher-salinity species’ pool compared to the lower-salinity species’ pool. This finding is consistent with previous studies showing the prevalence of macrocarnivorous and planktivorous diets among marine fish species and the prevalence of omnivorous, herbivorous and detritivorous diets among freshwater and brackish fish species [34]. Still, the increasing trend in the *CWM* of the trait related to the vegetal and detrital *vs*. animal components of the diet remained significant after partialling out salinity meaning that the increasing reliance on animal food was a pure effect of increasing naturalness. It is therefore crucial to take collinearity issues into account when investigating for changes in functional structure and assembly patterns along environmental gradients.

### Broader significance of the findings

Our findings illustrate that the functional structure of communities is a fertile conceptual framework to address restoration issues. Currently, studies routinely evaluate the species composition and relative abundance of fish communities, but including functional traits would add a very informative dimension to tidal wetland restoration studies. Monitoring the functional structure of fish communities helped identify the factors driving community change during tidal marsh restoration, especially through the shift of dominant trait values and the identity of traits affected by environmental filtering. In addition, the increased specialization of organisms and the broadening of ecological niches with restoration time were consistent with succession theory.

The approaches based on functional trait diversity have the potential to assist managers in their efforts to restore tidal wetland habitats. Those approaches allow to identify and manipulate the factors controlling community composition and, ultimately, to direct management towards a desired state of ecological functioning. The attractivity of benthic habitats for fish, both as feeding and living grounds, seemed to play a key role in tidal marsh restoration. In artificialized and restoring marshlands, wetland managers are thus challenged to improve benthic habitats, meaning for instance preventing excessive accumulation of sediments and organic matter. Considering a wide range of communities in future studies (e.g. fish, birds, plants and invertebrates) will give a more comprehensive view of tidal wetland response to restoration.

## Supporting information

S1 Table Description of the 13 fish sampling sites. (PDF)

S1 Fig Patterns of fish community assembly and monotonic trends in community-weighted means (*CWMs*) along the naturalness gradient of intertidal habitats. The X-axis represents the gradient of intertidal habitat naturalness (DYK, dyked marshes; TRM, tidally restored marshes; NINT, natural intertidal habitats) and the Y-axis represents the *CWM*. The names of the functional traits were in Table 2. Trends related to the *CWMs* were corrected for salinity using Spearman semipartial correlation. Ascending and descending straight lines represent, respectively, significant positive and negative correlation between the *CWMs* and intertidal naturalness. Horizontal straight lines indicate lack of significant correlation. Grey areas correspond to parts of the gradient where environmental filtering (i.e., lower-than-expected trait range) was detected under the null model 1 (trial swap). Striped areas indicate higher-than-expected trait range. Double arrows indicate either trait convergence (convergent arrows) or divergence (divergent arrows). Grey arrows indicate both lower significance (0.01 < *p* ≤ 0.05) and lower effect sizes (−0.5 < *medianES* < 0.5). Adapted from [14].
(TIFF)

S1 Appendix Functional characterization of the fish ecophases. (PDF)

S2 Appendix A null model to account for the species tolerance to salinity. (PDF)

S3 Appendix Computation of the effect size. (PDF)
