# Supplementary material for: Change in fish functional diversity and assembly rules in the course of tidal marsh restoration
Source: PLoS One. 2018 Dec 19;13(12):e0209025. doi: 10.1371/journal.pone.0209025 (PMC6300267; doi:10.1371/journal.pone.0209025)
Supplement: S1 Table — (PDF) [file pone.0209025.s001.pdf]

S1 Table. Description of the 13 fish sampling sites.

Sites group	Site code	Habitat type	Longitude	Latitude	Salinity			Temperature (°C)		
					Mean	Min.	Max.	Mean	Min.	Max.
Natural Intertidal Habitats (NINT)	IM1	Intertidal mudflat	-0.705475	45.120434	**2.65**	0.12	8.06	**17.26**	5.19	23.78
	IM2	Intertidal mudflat	-0.757260	45.265309	**6.86**	0.91	14.14	**17.61**	5.69	25.07
	IM3	Intertidal mudflat	-0.820745	45.360842	**11.86**	3.71	19.63	**16.92**	5.09	24.30
	IM4	Intertidal mudflat	-0.931138	45.439079	**18.46**	7.25	26.93	**17.16**	4.61	25.33
	IM5	Intertidal mudflat	-0.894669	45.528764	**18.71**	11.16	27.98	**16.06**	7.62	22.24
	ICH	Intertidal channel	-0.799479	45.470890	**13.16**	9.40	18.97	**17.06**	7.63	23.55
Tidally Restored Marshes (TRM)	TRM1	Tidally restored marsh (12-year-old)	-0.812690	45.483451	**13.77**	6.04	19.85	**18.21**	9.06	25.15
	TRM2a	Tidally restored marsh (1-year-old)	-0.691529	45.172037	**4.57**	0.42	7.08	**21.29**	14.46	24.69
	TRM2b	Tidally restored marsh (1-year-old)	-0.685478	45.159000	**3.86**	0.36	5.83	**21.37**	11.90	28.83
Dyked marshes (DYK)	FDM	(Freshwater) Dyked marsh	-0.743321	45.405583	**0.32**	0.18	0.37	**16.71**	8.83	22.47
	BDM1	(Brackish) Dyked marsh	-0.683413	45.137507	**3.47**	1.93	5.51	**21.26**	10.36	27.56
	BDM2	(Brackish) Dyked marsh	-0.688359	45.151276	**4.24**	1.35	5.61	**16.58**	10.06	20.36
	BDM3	(Brackish) Dyked marsh	-0.679588	45.140467	**3.81**	0.51	6.70	**20.62**	10.62	28.87
