# Supplementary figures and images for: Change in fish functional diversity and assembly rules in the course of tidal marsh restoration
Source: PLoS One. 2018 Dec 19;13(12):e0209025. doi: 10.1371/journal.pone.0209025 (PMC6300267; doi:10.1371/journal.pone.0209025)

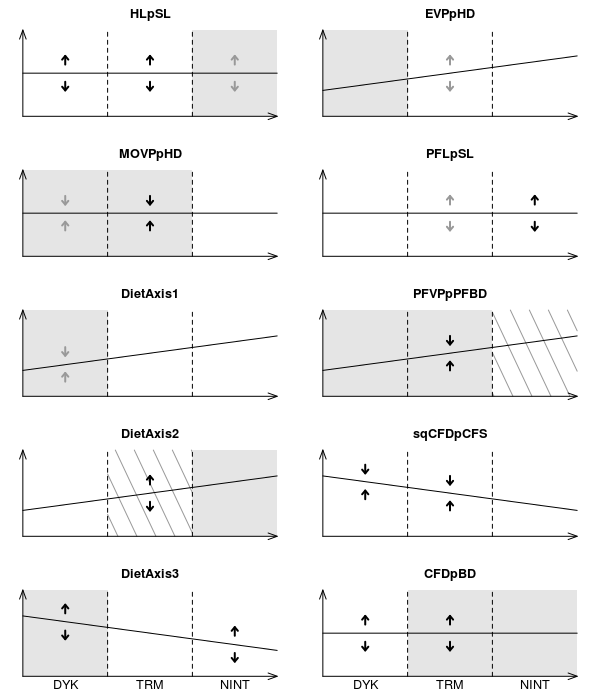

Supplement: S1 Fig — The X-axis represents the gradient of intertidal habitat naturalness (DYK, dyked marshes; TRM, tidally restored marshes; NINT, natural intertidal habitats) and the Y-axis represents the CWM. The names of the functional traits were in Table 2. Trends related to the CWMs were corrected for salinity using Spearman semipartial correlation. Ascending and descending straight lines represent, respectively, significant positive and negative correlation between the CWMs and intertidal naturalness. Horizontal straight lines indicate lack of significant correlation. Grey areas correspond to parts of the gradient where environmental filtering (i.e., lower-than-expected trait range) was detected under the null model 1 (trial swap). Striped areas indicate higher-than-expected trait range. Double arrows indicate either trait convergence (convergent arrows) or divergence (divergent arrows). Grey arrows indicate both lower significance (0.01 < p ≤ 0.05) and lower effect sizes (−0.5 < medianES < 0.5). Adapted from [14]. (TIFF) [file pone.0209025.s002.tiff]
