# Supplementary material for: Change in fish functional diversity and assembly rules in the course of tidal marsh restoration
Source: PLoS One. 2018 Dec 19;13(12):e0209025. doi: 10.1371/journal.pone.0209025 (PMC6300267; doi:10.1371/journal.pone.0209025)
Supplement: S1 Appendix — (PDF) [file pone.0209025.s003.pdf]

S1 Appendix. Functional characterization of the fish ecophases.

The functional characterization of fish ecophases was based on a multi-step procedure (Fig 1). For each species sampled, ontogenic changes in the functional niche were identified based on the diet shifts reported during growth (section 1). The food spectra of the fish ecophases were documented from published studies encompassing varied environments and summarized by a reduced number of synthetic, continuous variables (section 2). The diet-related functional traits were supplemented with ecomorphological traits related to food acquisition and swimming ability (section 3). Some functional traits were discarded *a posteriori* from the initial set to minimize redundancy in the functional characterization (section 4).

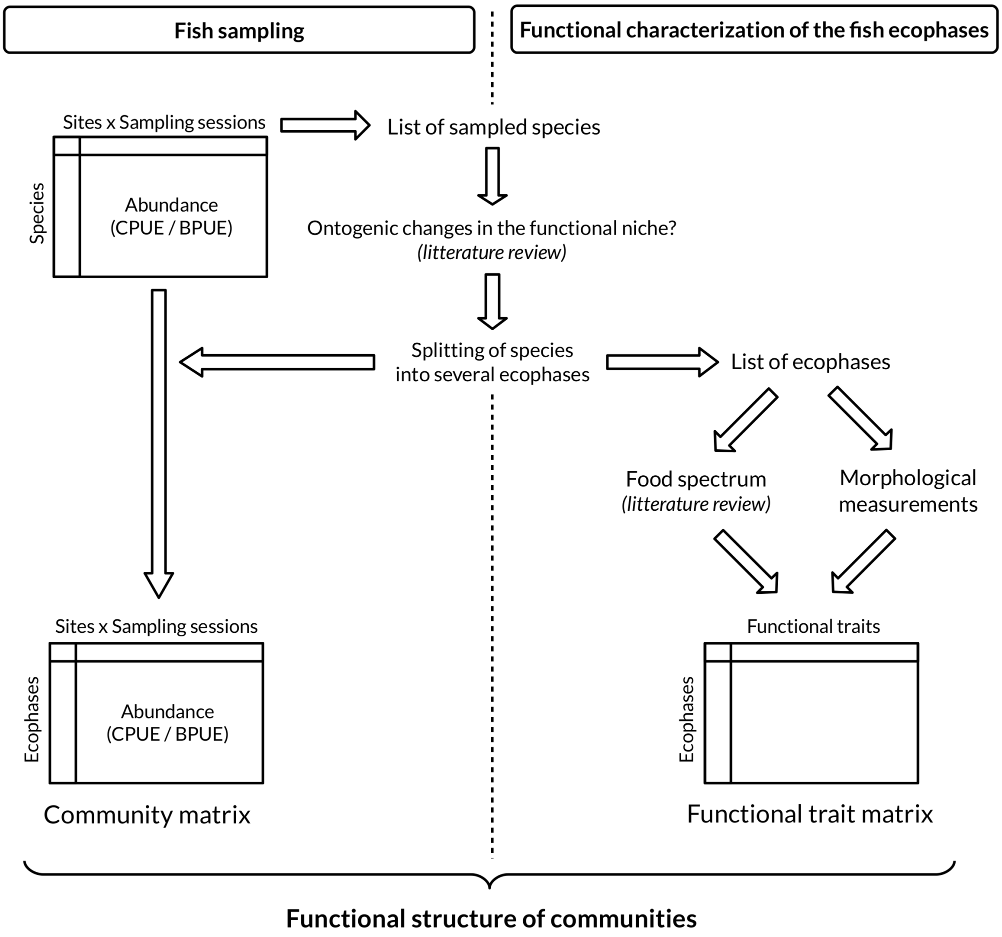

Figure 1 – Steps in the functional characterization of the fish ecophases.

1 Ontogenic changes in the functional niche

Fish species were subdivided into several ecophases to account for major ecological changes occurring during growth.

Diet and morphological shifts were shown to occur concurrently in the course of ontogeny for

several fish species (Hjelm et al., 2003 ; Russo et al., 2007 ; Zhao et al., 2014) and to be associated with broader ecological changes including swimming capacity and habitat selection (Russo et al., 2007). Compared to morphology, change in diet with size is rather well documented for numerous species. Therefore, we used diet shifts as proxys for more general ecological changes occurring during fish growth.

For each species, we only considered the size range sampled during our study. For example, changes in functional niche occurring in the wels catfish (*Silurus glanis*) larger than 300 mm were not reported because the maximum total length recorded in our data set was 163 mm.

The list and size limits of the sampled fish ecophases are reported in Table 1. The main references supporting the splitting of species according to diet shifts are provided in the same table.

2 Functional characterization of the food spectrum

The food spectrum of the ecophases was described from a review of the fish species' diets in varied environments. Most studies were based on stomach content analyses.

Our litterature review was not restricted to estuarine environments in agreement with the fact that functional traits are, by definition, independent from the environment (Violle et al., 2007). Therefore, the food spectrum of the ecophases was described based on the widest possible range of aquatic environments, accounting for the ecophases' plasticity in food resource exploitation (Funk et al., 2008 ; Comte et al., 2016).

2.1 The list of fish ecophases considered

The analysis of the food spectrum was carried out from 74 ecophases sampled in 2011-2012 as part of the 'Capalest' survey (Selleslagh et al., 2016). Those included the 62 ecophases recorded in the 13 dyked and intertidal habitats and an additional set of 12 ecophases that were solely sampled in the subtidal channel of the Gironde estuary. Subtidal sampling equipements included a beam trawl, a dragnet towed 0.2 m above the bottom and two rectangular frame nets fishing near the surface (Selleslagh et al., 2015).

2.2 Characterization of the food spectrum

Although a certain amount of subjectivity is inevitable when quantifying the importance of food items in the diet of a species from heterogenous methodologies, the following guidelines were adopted. The share of every food category in the diet of the fish ecophases was described according to

Table 1 – Identity and size class of the fish ecophases.

Species name	Ecophase	Ecophase code	Occurrence in data set	Length type	Min. length (mm)	Max. length (mm)	References
Abramis brama	Ecophase 1	AbrBraE1	+	FL	15	100	Giles et al., 1990; Kangur et al., 1999; Kakareko, 2001; Persson and Brönmark, 2002; Vašek et al., 2006; Specziár and Rezsu, 2009; Keith et al., 2011; Zapletal et al., 2012
Abramis brama	Ecophase 2	AbrBraE2	+	FL	100	260	
Alosa alosa	Ecophase 1	AloAloE1	(subtidal only)	FL	27	443	Taverny and Elie, 2001; Aprahamian et al., 2003
Alosa fallax	Ecophase 1	AloFalE1	+	FL	-	36	Aprahamian, 1989; Taverny and Elie, 2001; Aprahamian et al., 2003; Pasquaud, 2006
Alosa fallax	Ecophase 2	AloFalE2	+	FL	36	178	
Ameiurus melas	Ecophase 2	AmeMelE2	+	TL	50	250	Boët, 1981; Leunda et al., 2008; Keith et al., 2011
Ameiurus melas	Ecophase 3	AmeMelE3	+	TL	250	-	
Ammodytes tobianus	Ecophase 1	AmmTobE1	(subtidal only)	FL	53	189	Hostens and Mees, 1999; Kellnreitner et al., 2012
Anguilla anguilla	Ecophase 1	AngAngE1	+	TL	60	175	CTGREF, 1979; Lecomte-Finiger, 1983; Pasquaud, 2006
Anguilla anguilla	Ecophase 2	AngAngE2	+	TL	175	400	
Anguilla anguilla	Ecophase 3	AngAngE3	+	TL	400	-	
Aphia minuta	Ecophase 1	AphMinE1	(subtidal only)	TL	-	-	Baldó and Drake, 2002; La Mesa et al., 2005
Argyrosomus regius	Ecophase 1	ArgRegE1	+	TL	-	50	Cabral and Ohmert, 2001; Baldó and Drake, 2002; Pasquaud, 2006
Argyrosomus regius	Ecophase 2	ArgRegE2	+	TL	50	-	
Barbus barbus	Ecophase 2	BarBarE2	(subtidal only)	FL	118	-	Bischoff and Freyhof, 1999; Cherghou et al., 2002; Piria et al., 2005
Belone belone	Ecophase 2	BelBelE2	(subtidal only)	FL	48	-	Dorman, 1988; Dorman, 1991; Hostens and Mees, 1999; Sever et al., 2009
Blicca bjoerkna	Ecophase 1	BliBjoE1	+	FL	-	69	Specziár et al., 1997; Martyniak et al., 1999; Specziár et Rezsu, 2009; Keith et al., 2011
Blicca bjoerkna	Ecophase 2	BliBjoE2	+	FL	69	-	
Carassius gibelio	Ecophase 2	CarGibE2	+	FL	94	468	Specziár and Rezsu, 2009
Chelon labrosus	Ecophase 1	CheLabE1	+	FL	-	45	Cambrony, 1983; Gisbert et al., 1996; Cardona, 2001; Vandendriessche et al., 2007
Ciliata mustela	Ecophase 1	CilMusE1	+	TL	-	45	Costa, 1988; Hostens and Mees, 1999; Vandendriessche et al., 2007; Dolbeth et al., 2008
Ciliata mustela	Ecophase 2	CilMusE2	(subtidal only)	TL	45	-	
Conger conger	Ecophase 2	ConConE2	+	TL	200	-	Costa, 1988; Morato et al., 1999; Anastasopoulou et al., 2003; O'Sullivan et al., 2004; Xavier et al., 2010; Abi-Ayad et al., 2011; Matić-Skoko et al., 2012
Cyprinus carpio	Ecophase 2	CypCarE2	+	FL	22	132	Vilizzi and Walker, 1998; García-Berthou, 2001; Baldó and Drake, 2002; Khan, 2003; Nunn et al., 2007; Specziár and Rezsu, 2009
Cyprinus carpio	Ecophase 3	CypCarE3	+	FL	132	-	
Dicentrarchus labrax	Ecophase 1	DicLabE1	+	FL	-	40	Audousset, 1978; CTGREF, 1979; Barnabé, 1980; Ferrari and Chieregato, 1981; Laffaille et al., 2001; Hampel et al., 2005; Pasquaud, 2006; Dolbeth et al., 2008 ; Martinho et al., 2008; Fonseca et al., 2011; Green et al., 2012
Dicentrarchus labrax	Ecophase 2	DicLabE2	+	FL	40	115	
Dicentrarchus labrax	Ecophase 3	DicLabE3	+	FL	115	-	
Dicentrarchus punctatus	Ecophase 1	DicPunE1	+	FL	-	37	Baldó and Drake, 2002; Pasquaud, 2006; Pasquaud et al., 2008; Gushchin, 2013
Dicentrarchus punctatus	Ecophase 2	DicPunE2	+	FL	37	111	
Dicentrarchus punctatus	Ecophase 3	DicPunE3	+	FL	111	-	
Engraulis encrasicolus	Ecophase 1	EngEncE1	+	FL	-	-	Tudela and Palomera, 1997; Baldó and Drake, 2002; Pasquaud, 2006; Borme et al., 2009; Morote et al., 2010
Gambusia holbrooki	Ecophase 1	GamHolE1	+	TL	-	-	Mansfield and Mcardle, 1998; Cabral et al., 1999; García-Berthou, 1999; Blanco et al., 2004; Specziár, 2004; Pyke, 2005; Gkenas et al., 2012; Erguden, 2013
Gasterosteus aculeatus	Ecophase 1	GasAcuE1	+	TL	-	20	Hynes, 1950; Moore and Moore, 1976; Bergersen 1996; Sánchez-Gonzáles et al., 2001; Peltonen et al., 2004; McIntyre et al., 2006; Nunn et al., 2007; Dukowska et al., 2009; Niksirat et al., 2010; Spilseth and Simenstad, 2011; Kellnreitner et al., 2012; Nunn et al., 2012
Gasterosteus aculeatus	Ecophase 2	GasAcuE2	+	TL	20	75	
Gymnocephalus cernuus	Ecophase 2	GymCerE2	+	FL	35	140	Bergman, 1991; Bergman and Greenberg, 1994; Werner et al., 1996; Hölker and Thiel, 1998; Ogle et al., 2004; Rezsu and Specziár, 2006; Lorenzoni et al., 2007; Schleuter and Eckmann, 2008; Tarvainen et al., 2008; Specziár and Rezsu, 2009
Hippocampus hippocampus	Ecophase 1	HipHipE1	(subtidal only)	TL	69	104	Kitsos et al., 2008; Foster and Vincent, 2004
Lepomis gibbosus	Ecophase 1	LepGibE1	+	FL	-	35	Godinho et al., 1997; Wolfram-Wais et al., 1999; García-Berthou and Moreno-Amich, 2000; Declerck et al., 2002; Rezsu and Specziár, 2006; Specziár and Rezsu, 2009; Maazouzi et al., 2011
Lepomis gibbosus	Ecophase 2	LepGibE2	+	FL	35	118	
Lepomis gibbosus	Ecophase 3	LepGibE3	+	FL	118	-	
Liza ramada	Ecophase 1	LizRamE1	+	FL	-	42.5	CTGREF, 1979; Ferrari and Chieregato, 1981; Cambrony, 1983; Almeida et al., 1993; Gisbert et al., 1996; Cardona, 2001; Laffaille et al., 2002; Pasquaud, 2006
Liza ramada	Ecophase 2	LizRamE2	+	FL	42.5	-	
Platichthys flesus	Ecophase 1	PlaFleE1	+	TL	-	45	CTGREF, 1979; Pihl, 1985; Costa, 1988; Aarnio et al., 1996; Andersen et al., 2005; Hampel et al., 2005; Pasquaud, 2006; Nissling et al., 2007; Martinho et al., 2008; Pasquaud et al., 2010; Mendes et al., 2014
Platichthys flesus	Ecophase 2	PlaFleE2	+	TL	45	100	
Platichthys flesus	Ecophase 3	PlaFleE3	+	TL	100	-	
Pomatoschistus microps	Ecophase 1	PomMicE1	+	TL	-	40	Magnhagen and Wiederholm, 1982; Pihl, 1985; Doornbos and Twisk, 1987; Baldó and Drake, 2002; Hampel and Cattrijsse, 2004; Salgado et al., 2004; Pasquaud, 2006; Dolbeth et al., 2008; Kellnreitner et al., 2012
Pomatoschistus microps	Ecophase 2	PomMicE2	+	TL	40	-	
Pomatoschistus minutus	Ecophase 1	PomMinE1	+	TL	-	42.5	Pihl, 1985; Doornbos and Twisk, 1987; Costa, 1988; Hamerlynck and Cattrijsse, 1994; Hostens and Mees, 1999; Salgado et al., 2004; Pasquaud, 2006; Dolbeth et al., 2008
Pomatoschistus minutus	Ecophase 2	PomMinE2	+	TL	42.5	-	
Pseudorasbora parva	Ecophase 1	PseParE1	+	FL	-	28	Wolfram-Wais et al., 1999; Xie et al., 2000; Declerck et al., 2002; Park and Park, 2005; Xie et al., 2005; Gozlan et al., 2010; Shoniya et al., 2011; Bo et al., 2012
Pseudorasbora parva	Ecophase 2	PseParE2	+	FL	28	40	
Pseudorasbora parva	Ecophase 3	PseParE3	+	FL	40	-	
Raja clavata	Ecophase 1	RajClaE1	(subtidal only)	TL	-	-	Holden and Tucker, 1974; Pasquaud, 2006
Rutilus rutilus	Ecophase 2	RutRutE2	+	FL	40	120	Bohl, 1979; Persson, 1983; Jamet, 1994; Declerck et al., 2002; Hjelm et al., 2003; Lappalainen et al., 2004; Nunn et al., 2007; Horppila and Nurminen, 2009; Specziár and Reszu, 2009; Nurminen et al., 2010; Zapletal et al., 2014
Rutilus rutilus	Ecophase 3	RutRutE3	+	FL	120	-	
Salmo salar	Ecophase 1	SalSalE1	(subtidal only)	FL	91	182	Gamås and Hvidsten, 1985; Haugland et al., 2006; Jutila and Jokikokko, 2008; Keith et al., 2011; Shustov and Belyakova, 2012; Thorstad et al., 2012
Sander lucioperca	Ecophase 1	SanLucE1	+	FL	-	29	Popova and Sytina, 1977; Campbell, 1992; Hansson et al., 1997; Balik, 1999; Sutela and Hyvärinen, 2002; Peterka et al., 2003; Specziár, 2005; Persson and Brönmark, 2008; Specziár and Rezsu, 2009; Ginter et al., 2011; Keith et al., 2011; Argillier et al., 2012; Pérez-Bote and Roso, 2012; Yağci et al., 2014
Scardinius erythrophthalmus	Ecophase 1	ScaEryE1	+	FL	-	24	Prejs, 1984 *in* Kapuscinski et al., 2012; García-Berthou and Moreno-Amich, 2000; Horppila and Nurminen, 2009; Specziár and Rezsu, 2009; Keith et al., 2011; Kapuscinski et al., 2012; Nunn et al., 2012
Scardinius erythrophthalmus	Ecophase 2	ScaEryE2	+	FL	24	115	
Scardinius erythrophthalmus	Ecophase 3	ScaEryE3	+	FL	115	-	
Silurus glanis	Ecophase 3	SilGlaE3	+	TL	40	300	Carol et al., 2009; Copp et al., 2009
Solea senegalensis	Ecophase 2	SolSenE2	+	TL	50	-	Garcia-Franquesa et al., 1996; Cabral, 2000
Solea solea	Ecophase 1	SolSolE1	+	TL	-	50	Braber and de Groot, 1973; CTGREF, 1979; Costa, 1988; Hostens and Mees, 1999; Cabral, 2000; Amara et al., 2001; Pasquaud, 2006; Dolbeth et al., 2008; Martinho et al., 2008; Pasquaud et al., 2010
Solea solea	Ecophase 2	SolSolE2	+	TL	50	-	
Sparus aurata	Ecophase 1	SpaAurE1	+	FL	-	24	Ferrari and Chieregato, 1981; Tancioni et al., 2003; Russo et al., 2007
Sparus aurata	Ecophase 2	SpaAurE2	+	FL	24	64	
Sparus aurata	Ecophase 3	SpaAurE3	+	FL	64	-	
Sprattus sprattus	Ecophase 1	SprSprE1	+	FL	-	-	Thiel et al., 1996; Tičina et al., 2000; Peltonen et al., 2004; Pasquaud, 2006; Kellnreitner et al., 2012
Squalius cephalus	Ecophase 1	SquCepE1	+	FL	-	150	Hellawell, 1971; Mann, 1976; Piria et al., 2005; Balestrieri et al., 2006; Keith et al., 2011; Ünver and Erk'akan, 2011
Syngnathus acus	Ecophase 1	SynAcuE1	(subtidal only)	TL	-	150	Kendrick and Hyndes, 2005; Zupo and Stübing, 2010
Syngnathus rostellatus	Ecophase 1	SynRosE1	(subtidal only)	TL	-	80	Vandendriessche et al., 2007; Girardin and Castelnaud, 2013; Froese and Pauly, 2014
Syngnathus rostellatus	Ecophase 2	SynRosE2	+	TL	80	-	
Tinca tinca	Ecophase 2	TinTinE2	+	FL	140	-	Kennedy and Fitzmaurice, 1970; Giles et al., 1990; Michel and Oberdorff, 1995; Petridis, 1990; Benzer et al., 2007
Umbrina spp	Ecophase 2	UmbSppE2	(subtidal only)	FL	67	164	Zahorcsak et al., 2000; Froese and Pauly, 2014

'$+$' indicates that the ecophase was collected among the 13 sampling sites (i.e. dyked, restored or natural intertidal habitats) in 2011-2012 ; 'subtidal only' indicates that the ecophase was only collected in the subtidal parts of the Gironde estuary during concurrent sampling sessions. Abbreviations for length types : FL, Fork length ; TL, Total length.

three modalities : $P1$: primary importance ; $P2$: secondary importance ; Ab : accessory importance or absence from the diet. The food categories were treated as ordinal, semi-quantitative variables so that $Ab < P2 < P1$. $P1$ was attributed to food items of first rank importance in terms of biomass or volume. $P2$ was attributed to food items that represented 10% or more of the volume or biomass of total ingested food without reaching first rank importance. Ab was attributed to the remainder of the food taxa. For every food category, we retained the maximum contribution to the diet ($P1$, $P2$ or Ab) reported across studies ; however, the results considered as abnormal or aberrant by their authors were discarded.

As resolution or accuracy in taxonomic description differed substantially among studies and fish species, food taxa were pooled *a posteriori* into a reduced number of common food categories. When two or more food taxa were pooled, the contribution level of the most important taxon (i.e. Ab, $P2$ or $P1$) was affected to the aggregated category. A disadvantage of this aggregation rule is that it prevented the contribution categories to step up from lower to higher levels ; however, it was considered superior to any other automatic pooling procedure.

In total, eight aggregated food categories were considered : (1) microinvertebrates ; (2) eggs and macrofaunal larvae (teleost fish and macroinvertebrates) ; (3) annelids ; (4) arthropods (insects, collembolans, arachnids, eucarids, peracarids) ; (5) shelled molluscs ; (6) teleost fish (and cephalopods) ; (7) microphytes ; (8) macrophytes and detritus.

The food spectrum of the 74 fish ecophases is described in Table 2. A dimensionality reduction procedure was carried out to extract a reduced number of approximately continuous, ecologically interpretable variables from the food spectrum matrix.

2.3 Computation of the dissimilarity matrix

A Gower similarity matrix (S_{Gower}) was computed from the food spectrum matrix using the metric, asymmetric extension of Gower similarity to semi-quantitative variables (Podani, 1999). The formula corresponds to equation (3) in Podani (1999). We used the assymetric version of Gower similarity so that a food category simultaneously absent from the diet of two compared ecophases did not increase the similarity between those two ecophases.

The Gower dissimilarity (D_{Gower}) was computed as $\sqrt{1 - S_{Gower}}$ because $(1 - S_{Gower})$ yielded negative eigenvalues in the subsequent principal coordinates analysis. With the square-root transformation, the eigenvalues of D_{Gower} were all positive. This is the first transformation recommended by Legendre and Legendre (1998) when $(1 - S_{Gower})$ produces negative eigenvalues.

Table 2 – Food spectrum of the fish ecophases. Abbreviations : $P1$, Food item of primary importance ; $P2$, Food item of secondary importance ; $-$, Absent or negligible food item. The names of fish ecophases were in Table 1.

Ecophase code	Microinvertebrates	Eggs and macrofaunal larvae	Annelids	Arthropods	Shelled molluscs	Teleost fish and cephalopods	Microphytes	Macrophytes and detritus
AbrBraE1	P1	-	-	-	-	-	-	-
AbrBraE2	P1	-	-	P1	P2	-	-	P2
AloAloE1	P1	P1	-	P1	-	P1	-	-
AloFalE1	P1	-	-	-	-	-	-	P1
AloFalE2	P1	-	-	P1	-	P1	-	P1
AmeMelE2	-	-	P1	P1	-	P2	-	P1
AmeMelE3	-	-	-	P1	-	P1	-	P1
AmmTobE1	P1	P1	-	P2	-	-	-	-
AngAngE1	-	-	P1	P1	-	-	-	-
AngAngE2	-	-	P1	P1	-	-	-	-
AngAngE3	-	-	-	P1	-	-	-	-
AphMinE1	P1	P1	-	P1	-	-	-	-
ArgRegE1	-	-	-	P1	-	-	-	-
ArgRegE2	-	-	-	P1	-	P2	-	-
BarBarE2	-	-	-	P1	P1	-	P1	P1
BelBelE2	P2	P2	P2	P1	P2	P1	-	-
BliBjoE1	P1	-	-	-	-	-	-	-
BliBjoE2	P2	-	-	P1	P1	-	-	P1
CarGibE2	P1	-	-	-	-	-	P2	P1
CheLabE1	P1	P1	P1	P1	-	-	-	-
CilMusE1	P1	P2	-	P2	-	-	-	-
CilMusE2	-	-	-	P1	-	P2	-	-
ConConE2	-	-	-	P2	-	P1	-	-
CypCarE2	P1	-	P2	P1	P2	-	-	-
CypCarE3	P2	-	P1	P1	P1	-	-	P1
DicLabE1	P1	P1	-	P2	-	-	-	-
DicLabE2	P2	-	P1	P1	-	P2	-	-
DicLabE3	-	-	-	P1	-	P1	-	-
DicPunE1	P1	-	-	P1	-	-	-	-
DicPunE2	-	P1	P2	P1	-	P2	-	-
DicPunE3	-	-	-	P1	-	P1	-	-
EngEncE1	P1	P1	-	P1	-	-	-	-
GamHolE1	P1	-	-	P1	-	-	P2	P2
GasAcuE1	P1	-	-	-	-	-	-	-
GasAcuE2	P1	-	P2	P1	-	-	-	-
GymCerE2	P2	-	-	P1	P2	P2	-	-
HipHipE1	-	-	-	P1	-	-	-	P2
LepGibE1	P1	-	-	P2	-	-	-	-
LepGibE2	P2	-	-	P1	-	-	-	-
LepGibE3	-	-	-	P1	P1	P1	-	-
LizRamE1	P1	P1	P1	P1	-	-	P2	-
LizRamE2	P1	-	-	-	-	-	P1	P1
PlaFleE1	P1	-	P1	P1	-	-	-	-
PlaFleE2	-	-	P1	P1	-	-	-	-
PlaFleE3	-	-	P1	P1	P1	P2	-	-
PomMicE1	P1	P2	-	-	-	-	-	-
PomMicE2	P2	-	P1	P1	P1	-	-	-
PomMinE1	P1	-	P2	P2	-	-	-	-
PomMinE2	-	-	P1	P1	P1	-	-	-
PseParE1	P1	-	-	-	-	-	-	-
PseParE2	P1	-	-	P1	-	-	-	-
PseParE3	P2	-	-	P1	-	-	P2	P2
RajClaE1	-	-	P2	P1	-	P2	-	-
RutRutE2	P1	-	-	P1	P1	-	P1	P1
RutRutE3	-	-	-	P1	P1	-	P1	P1
SalSalE1	-	P1	-	P1	-	P1	-	-
SanLucE1	P1	-	-	-	-	-	-	-
ScaEryE1	P1	-	-	P1	-	-	-	-
ScaEryE2	-	-	-	P1	P2	-	P1	P1
ScaEryE3	-	-	-	P2	P2	P2	P1	P1
SilGlaE3	-	-	P1	P1	-	P1	-	P2
SolSenE2	-	-	P1	P1	P2	-	-	-
SolSolE1	P1	-	-	P2	-	-	-	-
SolSolE2	-	-	P1	P1	P2	-	-	-
SpaAurE1	P1	P1	P1	P2	-	-	-	-
SpaAurE2	-	-	P1	P1	-	-	-	-
SpaAurE3	-	-	P1	P1	P1	P2	-	-
SprSprE1	P1	P1	-	-	-	-	-	-
SquCepE1	P2	-	-	P1	-	-	P1	-
SynAcuE1	P1	-	P2	P1	-	-	-	-
SynRosE1	P1	-	-	-	-	-	-	-
SynRosE2	P1	P1	-	-	-	-	-	-
TinTinE2	P2	-	-	P1	P1	-	-	P2
UmbSppE2	-	-	-	P1	-	-	-	-

2.4 Principal coordinates analysis

The principal coordinates analysis (PCoA) was carried out on R software using the function $pcoa$ of the 'ape' package.

The relative eigenvalues of the PCoA are plotted on Fig 2. The first axis of the PCoA accounted

for 25.8% of the cumulative sum of eigenvalues. The sum of the relative eigenvalues of the first three axes amounted to 48.0%. Three axes were retained to summarize the food spectrum of the ecophases.

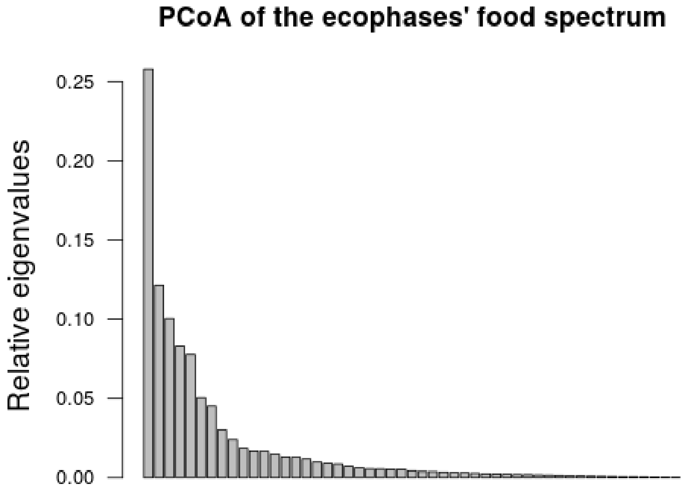

Figure 2 – Relative eigenvalues of the PCoA based on the food spectrum of the fish ecophases.

The ecophases and the variables describing the food spectrum were plotted according to the first three axes of the PCoA ordination (Figs 3 and 4). The contribution of each original variable (i.e. food category) to a given PCoA axis was measured by the magnitude of the Spearman correlation coefficient between the (ordinal) variable and the ecophases' coordinates along the PCoA axis.

The correlation strength between the ecophases' coordinates on the PCoA axes and each of the eight food categories was computed *a posteriori* using Spearman correlation coefficient (Fig 5).

2.5 Ecological interpretation of the first axes of the PCoA

Two groups of food categories were clearly separated along axis 1 (Fig 5) : (1), microinvertebrates ($\rho = 0.88$) and, to a lesser extent, eggs and macrofaunal larvae ($\rho = 0.36$; positive values of the axis) ; (2), arthropods ($\rho = -0.75$) and, to a lesser extent, annelids ($\rho = -0.48$), shelled molluscs and fish ($\rho = -0.40$; negative values of the axis). We interpreted axis 1 as a gradient of animal prey size, opposing small-sized prey (mainly, microinvertebrates) to medium- and large-sized prey.

The second axis mainly segregated two groups of food categories : (1), macrophytes and detritus ($\rho = -0.74$) and microphytes ($\rho = -0.52$; negative values of the axis) ; (2), annelids ($\rho = 0.59$) and eggs and macrofaunal larvae ($\rho = 0.44$; positive values of the axis). At its most basic, the second axis of the PCoA corresponded to a detrital and vegetal vs. animal food components of the diet. The most extreme values on the animal side of the axis are occupied by soft-bodied prey.

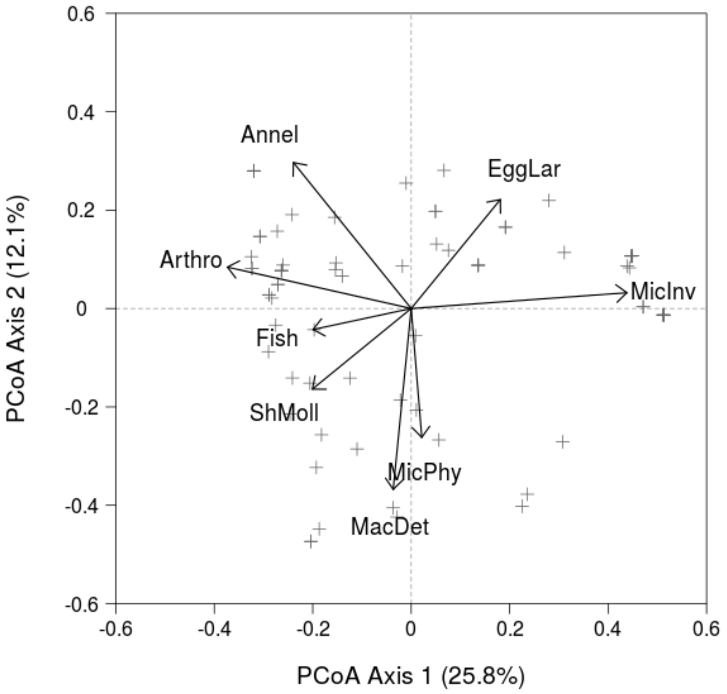

Figure 3 – Ordination of the fish ecophases according to their food spectrum along the axes 1 and 2 of the PCoA. The percentage values are the relative eigenvalues of the PCoA axes. The grey crosses represent individual ecophases. The coordinates of the tip of the arrows are proportional to the variable positive or negative correlation (Spearman's rho) with the ecophases scores on the PCoA axes. The absolute length of the arrows is meaningless. Abbreviations for food categories : $MicInv$, Microinvertebrates ; $EggLar$, Eggs and macrofaunal larvae ; $Annel$, Annelids ; $Arthro$, Arthropods ; $ShMoll$, Shelled molluscs ; $Fish$, Teleost fish and cephalopods ; $MicPhy$, Microphytes ; $MacDet$, Macrophytes and detritus.

The third axis separated fish ($\rho = 0.61$) from annelids ($\rho = -0.57$) and shelled molluscs ($\rho = -0.41$). Thus, the third axis opposed large-sized mobile prey (fish) to medium-sized, low-mobility benthic prey. Fish ecophases with high positive values along axis 3 are potential predators.

3 Functional traits based on morphological measurements

3.1 General considerations

Nine ecomorphological (functional) traits were selected to describe fish feeding and swimming behaviour (Table 4). Those functional traits were based on 12 morphological measurements (Fig 6 and Table 3). The ecological performances associated with the functional traits are summarized in Table 4.

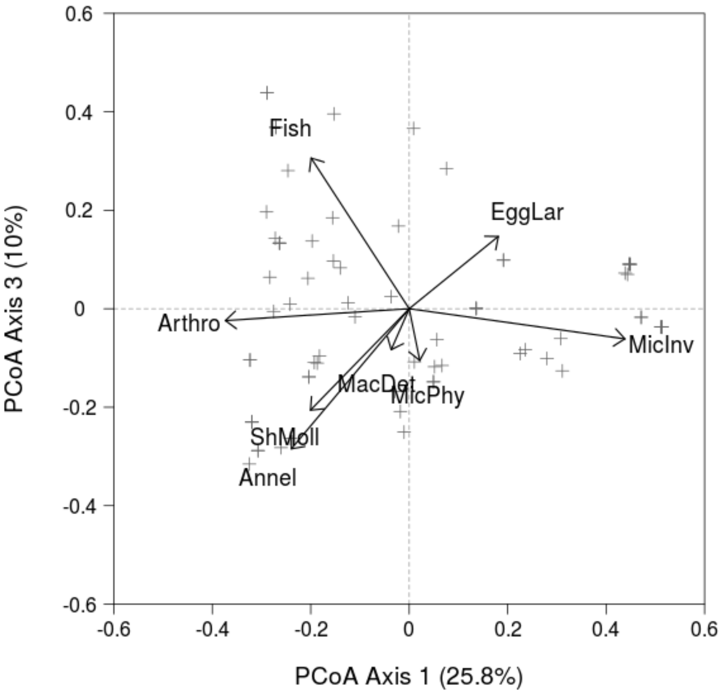

Figure 4 – Ordination of the fish ecophases according to their food spectrum along the axes 1 and 3 of the PCoA. The percentage values are the relative eigenvalues of the PCoA axes. The grey crosses represent individual ecophases. The coordinates of the tip of the arrows are proportional to the variable positive or negative correlation (Spearman's rho) with the ecophases scores on the PCoA axes. The absolute length of the arrows is meaningless. Abbreviations for food categories : $MicInv$, Microinvertebrates ; $EggLar$, Eggs and macrofaunal larvae ; $Annel$, Annelids ; $Arthro$, Arthropods ; $ShMoll$, Shelled molluscs ; $Fish$, Teleost fish and cephalopods ; $MicPhy$, Microphytes ; $MacDet$, Macrophytes and detritus.

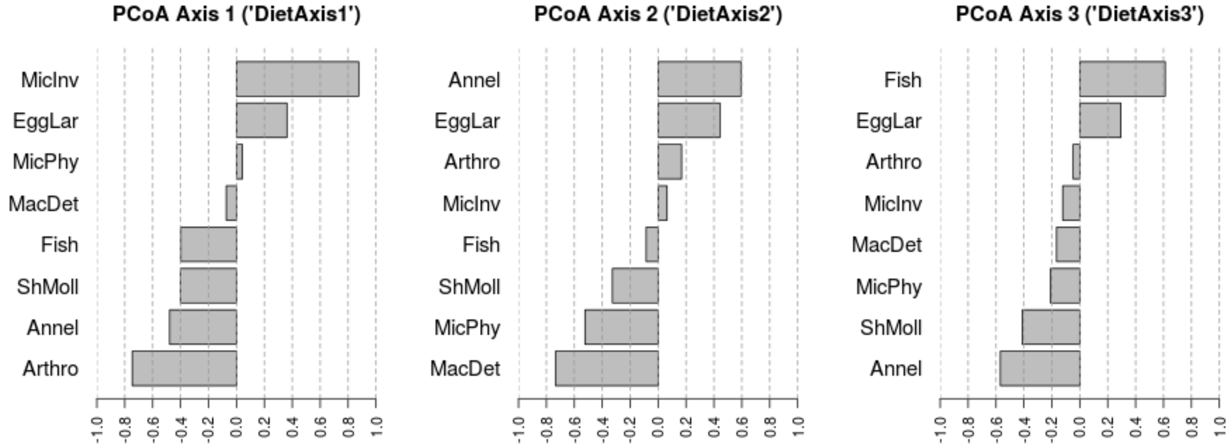

Figure 5 – Correlation between the first axes of the PCoA and each of the eight food categories. Abbreviations for food categories : $MicInv$, Microinvertebrates ; $EggLar$, Eggs and macrofaunal larvae ; $Annel$, Annelids ; $Arthro$, Arthropods ; $ShMoll$, Shelled molluscs ; $Fish$, Teleost fish and cephalopods ; $MicPhy$, Microphytes ; $MacDet$, Macrophytes and detritus.

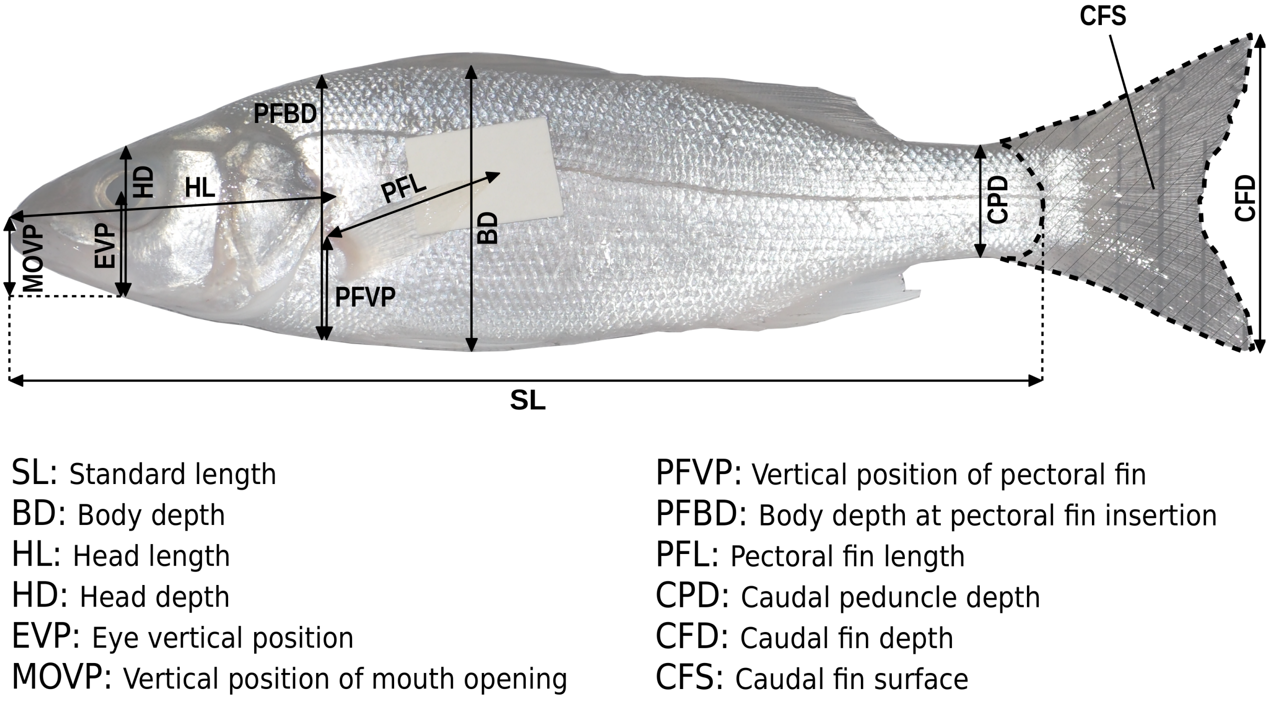

Figure 6 – Morphological measurements on fish ecophases

Table 3 – Definition of morphological measurements.

Morphological measurement	Abbr.	Definition	References
Standard length	SL	Horizontal distance from the anteriormost part of the snout to the end of the terminal vertebra.	Nakamura (1985)
Body depth	BD	Maximum body depth.	Gatz (1979), Dumay (2003), Villéger (2008)
Head length	HL	Oblique distance from the anteriormost part of the head (jaws closed) to the posteriormost point of the opercular bone, excluding spines and the gill membrane.	Holčik et al. (1989)
Head depth	HD	Head depth along the vertical axis crossing eye position. For flatflish, vertical axis crossing the center of the pupil of the lower eye.	Winemiller (1991), Villéger (2008)
Vertical position of the eye	EVP	Vertical distance between the center of pupil and the ventral side of the head.	Winemiller (1991), Villéger (2008)
Vertical position of mouth opening	MOVP	Vertical distance between the (lower part of the) anterior tip of the upper jaw and the ventral side of the head along the vertical axis crossing eye position.	Villéger (2008)
Vertical position of pectoral fin	PFVP	Vertical distance from the ventral side to the insertion of the pectoral fin.	Adapted from Schleuter et al. (2012)
Body depth at pectoral fin insertion	PFBD	Body depth at the insertion of the pectoral fin.	Schleuter et al. (2012)
Pectoral fin length	PFL	Oblique distance from the base of the pectoral fin to the extreme tip of the fin at its longest point.	Gatz (1979), Villéger (2008)
Caudal peduncle depth	CPD	Least vertical distance along a straight line between the dorsal and the ventral surfaces of the caudal peduncle.	Holčik et al. (1989), Winemiller (1991)
Caudal fin depth	CFD	Vertical dimension of the smallest rectangle in which the splayed (but not stretched) caudal fin can be entirely contained.	Adapted from Gatz (1979)
Caudal fin surface	CFS	Surface of the caudal fin including scale cover.	Gatz (1979)

Distances are straight line distances. Abbr., Abbreviation.

Table 4 – Functional traits selected *a priori*.

Niche axis	Functional trait	Abbreviation	Computation	Ecological performance	References
Diet and food acquisition	Relative head length	*HLpSL*	$\frac{HL}{SL}$	Succion capacity (+)<br>Filtration capacity (+)<br>Maximum size of ingested animal prey (+)<br>Share of plants and detritus in the diet (−)	Gatz (1979) ; Piet (1998) ; Sibbing and Nagelkerke (2001)
	Vertical position of mouth opening	*MOVPpHD*	$\frac{MOVP}{HD}$	Vertical position of food in the water column (+)	Villéger (2008) ; Schleuter et al. (2012)
	Axis 1 of food spectrum	*DietAxis1*	*PCoA, axis 1*	Micro-sized animal prey (microinvertebrates) (+)<br>Medium- and larger-sized animal prey (−)	This appendix (section 2)
	Axis 2 of food spectrum	*DietAxis2*	*PCoA, axis 2*	Vegetal and detrital food (−)<br>Soft-bodied animal prey (+)	This appendix (section 2)
	Axis 3 of food spectrum	*DietAxis3*	*PCoA, axis 3*	Larger-sized mobile prey (+)<br>Low-mobility, medium-sized benthic prey (−)	This appendix (section 2)
Swimming capacity and passive behaviour	Vertical position of the eye	*EVPpHD*	$\frac{EVP}{HD}$	Vertical position of the fish in the water column (−)<br>Amount of swimming (mobile *vs.* sedentary behaviour) (−)	Gatz (1979)
	Relative body depth	*BDpSL*	$\frac{BD}{SL}$	Acceleration capacity (+),<br>Maneuvering capacity (+),<br>Energetic cost of sustained swimming (+)<br>Current velocity in the habitat (−)<br>Vulnerability to predation (−)	Nikolski, 1933 ; Gatz, 1979 ; Webb, 1984 ; Vøllstad et al., 2004 ; Nilsson et al., 1995
	Relative length of the pectoral fin	*PFLpSL*	$\frac{PFL}{SL}$	Current velocity in the habitat (−)<br>Maneuvering capacity (+)	Gatz (1979)
	Vertical insertion of the pectoral fin	*PFVPpPFBD*	$\frac{PFVP}{PFBD}$	Turning capacity (+)	Dumay et al. (2004) *in* Villéger et al. (2010) ; Schleuter et al. (2012)
	Throttling of the caudal peduncle	*CFDpCPD*	$\frac{CFD}{CPD}$	Acceleration capacity (−)<br>Energetic cost of sustained swimming (−)	Blake (2004) *in* Villéger (2008) ; Webb (1984, 1988) *in* Sibbing and Nagelkerke (2001)
	Relative depth of the caudal fin	*CFDpBD*	$\frac{CFD}{BD}$	Energetic cost of sustained swimming (−)	Gatz (1979)
	Aspect ratio of the caudal fin	*sqCFDpCFS*	$\frac{CFD^2}{CFS}$	Energetic cost of sustained swimming (−)<br>Acceleration capacity (−)<br>Maneuvering capacity (−)	Keast and Webb (1966) ; Gatz (1979) ; Mahon (1984) ; Villéger (2008)

Morphological measurements are detailed in Fig 6 and Table 3.

3.2 Conventions for peculiar morphologies

The computation of some ecomorphological traits was adjusted to account for peculiar morphologies following Villéger (2008) and Villéger et al. (2010).

Lateralized swimming and settling behaviour in Pleuronectiform species (*Solea solea*, *Solea senegalensis* and *Platichthys flesus*) required adjustments in the computation of the vertical position

of the mouth opening ($MOVPpHD$), the vertical position of the eye ($EVPpHD$), the relative body depth ($BDpSL$) and the vertical insertion of the pectoral fin ($PFVPpPFBD$). Body depth (BD) was replaced by body width (i.e. the maximum vertical distance between the eyed and the blind side) in the computation of $BDpSL$. As eyes are positioned on the uppermost side the body, $EVPpHD$ was set to 1. $MOVPpHD$ was set to 0. Given the low manoeuvering and turning capacity of flatfish, we solely considered the pectoral fin of the blind side when computing $PFVPpPFBD$ (i.e., $PFVPpPFBD$ was set to 0).

The caudal fin in Anguilliform species (*Anguilla anguilla*, *Conger conger*) is not distinct from dorsal and anal fins (Villéger, 2008). The relative depth of the caudal fin ($CFDpBD$) was set to 0 emphasizing its extreme shortening in Anguilliform species. $CFDpCPD$ was set to 1 in the absence of caudal peduncle. The aspect ratio of the caudal fin ($sqCFDpCFS$) was ascribed the intermediary value (among the pool of sampled ecophases) of 2.30 because Anguilliform species are neither free-swimming fish nor acceleration specialists.

3.3 Documenting the functional trait matrix

The ecomorphological traits were measured on a set of 283 individuals belonging to the 62 ecophases (34 species) sampled in the 13 dyked and intertidal habitats. The median number of measured individuals per ecophase was 4 ($min = 1$; $max = 17$). Eighty-four individuals were measured from pictures, of which 47 originated from Iglésias (2013) and 30 from Lanoiselée (2004). Twenty-two early juveniles of gilt-head sea bream *Sparus aurata* were collected from a hatchery. The remainder of the fish (177) were collected *a posteriori* in the Gironde estuary or in adjoining marine or continental hydrosystems. The morphological characteristics were measured in the laboratory on fresh or defrosted individuals.

The ecomorphological traits were aggregated per ecophase using weighted medians. Individual measurements were weighted to account for the integrity of the fish body parts and the quality of the source pictures. The filled functional trait matrix is supplied in Table 5.

4 Minimizing redundancy in the functional characterization

Redundancy in the functional characterization of the fish ecophases was assessed based on the Spearman correlation coefficients between the pairwise combinations of traits (Fig 7). Two traits ($BDpSL$ and $CFDpCPD$) were discarded so that the correlation coefficient between any pair of traits did not exceed 0.60.

Table 5 – Functional trait values.

Ecophase code	n	HLpSL	MOVPpHD	DietAxis1	DietAxis2	DietAxis3	EVPpHD	BDpSL	PFLpSL	PFVPpPFBD	CFDpCPD	CFDpBD	sqCFDpCFS
AbrBraE1	2	0.251	0.430	0.512	-0.013	-0.037	0.512	0.312	0.205	0.222	4.003	1.296	3.808
AbrBraE2	3	0.234	0.452	0.010	-0.206	-0.108	0.559	0.389	0.217	0.252	3.707	1.131	3.485
AloFalE1	3	0.307	0.558	0.308	-0.271	-0.061	0.573	0.267	0.191	0.151	4.013	1.598	4.590
AloFalE2	2	0.271	0.642	-0.022	-0.186	0.168	0.630	0.266	0.171	0.159	2.764	1.037	2.498
AmeMelE2	6	0.272	0.567	-0.275	-0.034	-0.005	0.769	0.255	0.177	0.442	2.694	1.306	2.038
AmeMelE3	1	0.338	0.438	-0.246	-0.214	0.281	0.780	0.301	0.182	0.592	2.676	1.117	2.046
AngAngE1	3	0.120	0.548	-0.320	0.280	-0.230	0.783	0.064	0.030	0.447	1.000	0.000	2.300
AngAngE2	4	0.117	0.563	-0.320	0.280	-0.230	0.779	0.071	0.044	0.547	1.000	0.000	2.300
AngAngE3	4	0.111	0.580	-0.264	0.077	0.133	0.753	0.069	0.047	0.561	1.000	0.000	2.300
ArgRegE1	6	0.323	0.530	-0.264	0.077	0.133	0.735	0.311	0.216	0.413	2.415	0.798	1.057
ArgRegE2	5	0.296	0.419	-0.271	0.049	0.368	0.701	0.315	0.201	0.375	2.243	0.734	1.151
BliBjoE1	9	0.260	0.515	0.512	-0.013	-0.037	0.527	0.300	0.187	0.229	3.675	1.309	3.189
BliBjoE2	5	0.248	0.415	-0.193	-0.323	-0.109	0.522	0.364	0.209	0.223	4.056	1.263	3.699
CarGibE2	2	0.243	0.540	0.236	-0.378	-0.083	0.569	0.419	0.188	0.197	2.920	1.097	2.735
CheLabE1	1	0.286	0.595	0.067	0.281	-0.115	0.549	0.203	0.170	0.731	2.948	1.452	2.080
CilMusE1	5	0.221	0.288	0.438	0.086	0.072	0.561	0.168	0.152	0.695	2.561	0.888	1.228
ConConE2	2	0.150	0.510	0.009	-0.055	0.366	0.774	0.068	0.054	0.556	1.000	0.000	2.300
CypCarE2	2	0.343	0.404	-0.018	0.086	-0.209	0.598	0.358	0.212	0.172	2.954	1.090	2.640
CypCarE3	5	0.293	0.463	-0.241	-0.141	-0.263	0.710	0.340	0.192	0.195	3.147	1.251	2.850
DicLabE1	6	0.325	0.563	0.449	0.107	0.090	0.603	0.226	0.137	0.378	2.378	1.153	1.671
DicLabE2	4	0.326	0.539	-0.242	0.191	0.009	0.637	0.252	0.170	0.353	2.678	1.111	2.126
DicLabE3	5	0.308	0.522	-0.289	0.028	0.438	0.691	0.273	0.188	0.407	2.883	1.098	2.471
DicPunE1	3	0.338	0.556	0.136	0.088	0.001	0.599	0.250	0.181	0.336	2.613	1.261	2.363
DicPunE2	7	0.321	0.584	-0.156	0.185	0.185	0.657	0.280	0.167	0.356	2.434	1.025	1.928
DicPunE3	2	0.313	0.685	-0.289	0.028	0.438	0.725	0.269	0.160	0.357	2.981	1.316	2.477
EngEncE1	3	0.255	0.446	0.191	0.165	0.099	0.623	0.175	0.105	0.222	3.276	1.500	3.540
GamHolE1	7	0.252	0.676	0.056	-0.267	-0.063	0.569	0.237	0.228	0.488	1.662	0.992	1.083
GasAcuE1	3	0.293	0.514	0.512	-0.013	-0.037	0.581	0.228	0.146	0.484	3.754	0.860	1.721
GasAcuE2	8	0.293	0.590	0.049	0.198	-0.148	0.644	0.250	0.195	0.530	5.239	0.963	2.209
GymCerE2	12	0.324	0.324	-0.197	-0.043	0.138	0.752	0.272	0.221	0.428	3.377	1.132	2.373
LepGibE1	1	0.351	0.462	0.471	0.004	-0.017	0.648	0.386	0.262	0.371	2.050	0.691	1.734
LepGibE2	9	0.367	0.520	-0.140	0.066	0.083	0.650	0.412	0.298	0.346	2.735	0.854	2.234
LepGibE3	1	0.346	0.672	-0.290	-0.088	0.197	0.735	0.476	0.282	0.350	2.472	0.760	1.983
LizRamE1	8	0.277	0.660	0.051	0.131	-0.118	0.546	0.247	0.177	0.642	2.266	1.034	1.623
LizRamE2	10	0.267	0.617	0.225	-0.402	-0.091	0.488	0.216	0.182	0.591	2.543	1.275	1.895
PlaFleE1	7	0.310	0.000	-0.010	0.255	-0.251	1.000	0.105	0.110	0.000	2.848	0.683	1.669
PlaFleE2	3	0.309	0.000	-0.320	0.280	-0.230	1.000	0.104	0.125	0.000	2.698	0.639	1.543
PlaFleE3	5	0.276	0.000	-0.323	0.082	-0.104	1.000	0.109	0.141	0.000	3.181	0.703	1.797
PomMicE1	13	0.280	0.638	0.443	0.083	0.070	0.867	0.192	0.250	0.771	2.308	1.066	1.260
PomMicE2	1	0.264	0.661	-0.260	0.088	-0.282	0.851	0.166	0.210	0.726	2.305	1.067	1.077
PomMinE1	1	0.266	0.550	0.311	0.114	-0.126	0.865	0.161	0.188	0.724	2.587	1.325	1.680
PomMinE2	6	0.263	0.653	-0.325	0.105	-0.315	0.860	0.170	0.225	0.726	2.627	1.280	1.468
PseParE1	3	0.250	0.742	0.512	-0.013	-0.037	0.598	0.238	0.139	0.193	3.496	1.708	3.568
PseParE2	3	0.248	0.648	0.136	0.088	0.001	0.592	0.247	0.158	0.203	3.132	1.664	3.450
PseParE3	5	0.221	0.646	-0.110	-0.285	-0.016	0.548	0.244	0.180	0.231	3.526	1.627	3.137
RutRutE2	6	0.241	0.485	-0.029	-0.424	-0.168	0.541	0.281	0.187	0.237	4.147	1.484	3.611
RutRutE3	2	0.217	0.418	-0.204	-0.473	-0.138	0.537	0.302	0.188	0.221	3.762	1.216	2.983
SanLucE1	2	0.321	0.569	0.512	-0.013	-0.037	0.628	0.223	0.132	0.418	2.469	1.172	1.551
ScaEryE1	1	0.317	0.615	0.136	0.088	0.001	0.594	0.234	0.128	0.214	2.285	0.909	1.869
ScaEryE2	4	0.247	0.669	-0.187	-0.448	-0.110	0.517	0.330	0.218	0.208	3.534	1.207	3.462
ScaEryE3	4	0.227	0.577	-0.037	-0.405	0.025	0.543	0.354	0.209	0.222	3.543	1.078	3.381
SilGlaE3	1	0.181	0.584	-0.283	0.021	0.064	0.759	0.198	0.152	0.573	2.522	0.623	1.333
SolSenE2	4	0.202	0.000	-0.307	0.147	-0.288	1.000	0.070	0.090	0.000	1.817	0.570	1.559
SolSolE1	1	0.229	0.000	0.471	0.004	-0.017	1.000	0.073	0.069	0.000	1.304	0.334	0.939
SolSolE2	13	0.214	0.000	-0.307	0.147	-0.288	1.000	0.081	0.067	0.000	1.621	0.446	1.302
SpaAurE1	6	0.294	0.358	0.280	0.220	-0.101	0.554	0.281	0.211	0.408	2.863	1.115	1.750
SpaAurE2	17	0.335	0.310	-0.320	0.280	-0.230	0.592	0.327	0.233	0.406	3.224	1.072	2.150
SpaAurE3	2	0.305	0.387	-0.323	0.082	-0.104	0.700	0.404	0.336	0.352	3.900	0.864	2.774
SprSprE1	4	0.261	0.678	0.446	0.107	0.090	0.611	0.240	0.144	0.204	2.826	1.193	2.946
SquCepE1	1	0.246	0.623	-0.124	-0.141	0.012	0.695	0.213	0.179	0.289	2.953	1.386	2.497
SynRosE2	7	0.140	0.676	0.446	0.107	0.090	0.685	0.040	0.026	0.681	8.798	1.707	2.029
TinTinE2	2	0.267	0.546	-0.182	-0.257	-0.096	0.681	0.316	0.201	0.187	2.157	1.115	2.102

The names of the fish ecophases were in Table 1. The names of functional traits were in Table 4.
n : number of measured individuals (morphological measurements).

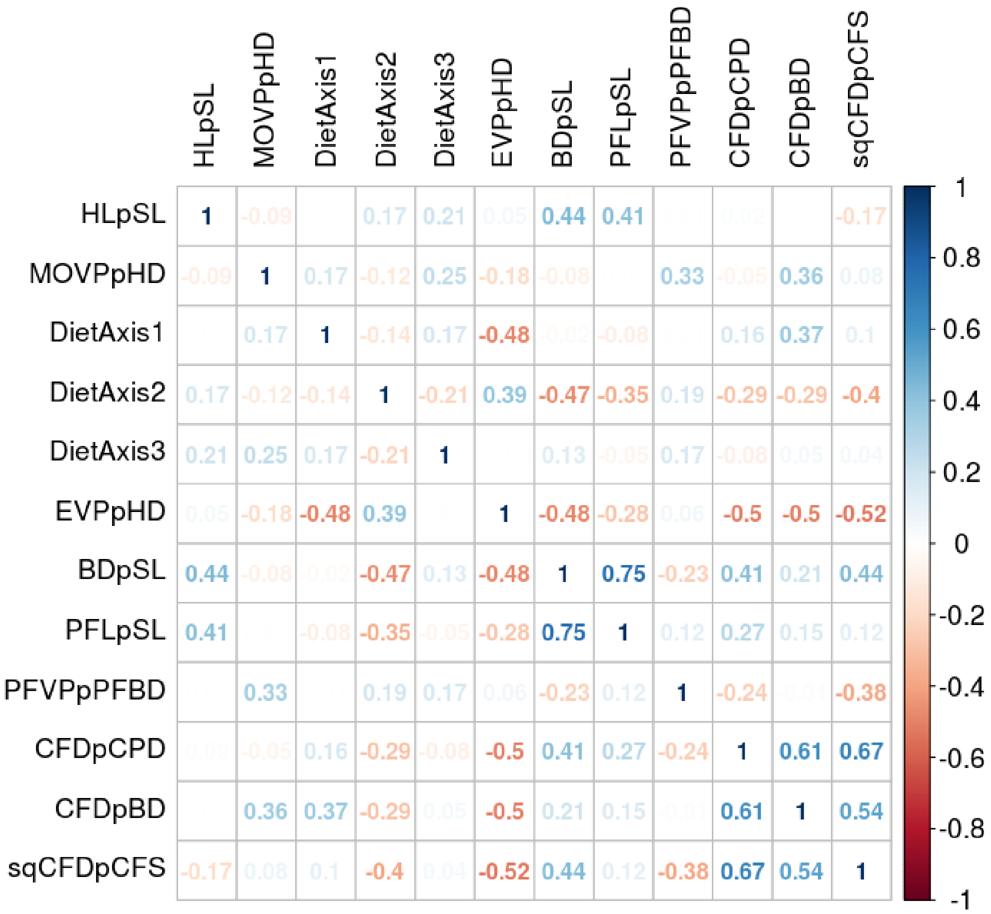

Figure 7 – Correlation between functional traits. Values are Spearman correlation coefficients. The names of functional traits were in Table 4.

References

Aarnio, K., Bonsdorff, E., Rosenback, N., 1996. Food and feeding habits of juvenile flounder Platichthys flesus (L.), abd turbot Scophthalmus maximus L. in the åland archipelago, northern Baltic Sea. Journal of Sea Research 36, 311–320. https ://doi.org/10.1016/S1385-1101(96)90798-4

Abi-Ayad, S.-M.E.-A., Kerkouf, S.A., Mehidi, S.A., Saddikioui, L., Ben SahlaTalet, A., 2011. Données préliminaires sur la composition du régime alimentaire du congre (Conger conger, Linnaeus, 1758) de la côte occidentale algérienne. European Journal of Scientific Research 64, 314–324.

Almeida, P.R., Moreira, F., Costa, J.L., Assis, C.A., Costa, M.J., 1993. The feeding strategies of Liza ramada (Risso, 1826) in fresh and brackish water in the River Tagus, Portugal. Journal of Fish Biology 42, 95–107. https ://doi.org/10.1111/j.1095-8649.1993.tb00308.x

Amara, R., Laffargue, P., Dewarumez, J.M., Maryniak, C., Lagardére, F., Luzac, C., 2001. Feeding ecology and growth of 0-group flatfish (sole, dab and plaice) on a nursery ground (Southern Bight of the North Sea). Journal of Fish Biology 58, 788–803. https ://doi.org/10.1111/j.1095-8649.2001.tb00531.x

Anastasopoulou, A., Mytilineou, C., Lefkaditou, E., Kavadas, S., Bekas, P., Smith, C.J., Papadopoulou, K.N., Christides, G., 2013. The diet and feeding ecology of Conger conger (L. 1758) in the deep waters of the Eastern Ionian Sea. Mediterranean Marine Science 14, 365–368. https ://doi.org/10.12681/mms.479

Andersen, B.S., Carl, J.D., Grønkjær, P., Støttrup, J.G., 2005. Feeding ecology and growth of age 0 year Platichthys flesus (L.) in a vegetated and a bare sand habitat in a nutrient rich fjord. Journal of Fish Biology 66, 531–552. https ://doi.org/10.1111/j.0022-1112.2005.00620.x

Aprahamian, M.W., 1989. The diet of juvenile and adult twaite shad Alosa fallax fallax (lacépède) from the rivers Severn and Wye (Britain). Hydrobiologia 179, 173–182. https ://doi.org/10.1007/BF00007604

Aprahamian, M.W., Aprahamian, C.D., Baglinière, J.L., Sabatié, R., Alexandrino, P., 2003. Alosa alosa and Alosa fallax spp. : literature review and bibliography. Environment Agency, Warrington, UK.

Argillier, C., Barral, M., Irz, P., 2012. Growth and diet of the pikeperch Sander lucioperca (L.) in two French reservoirs. Archives of Polish Fisheries 20, 191–200. https ://doi.org/10.2478/v10086-012-0024-0

Audousset, D., 1978. Le bar (Dicentrarchus labrax) : zoologie et élevage (Thèse pour le doctorat vétérinaire). Faculté de Médecine de Créteil.

Baldó, F., Drake, P., 2002. A multivariate approach to the feeding habits of small fishes in the Guadalquivir Estuary (SW Spain).

Balestrieri, A., Prigioni, C., Remonti, L., Sgrosso, S., Priore, G., 2006. Feeding ecology of Leuciscus cephalus and Rutilus rubilio in southern Italy. Italian Journal of Zoology 73, 129–135.

Balik, İ., 1999. The Feeding Features of The Pike-perch (Stizostedion lucioperca) Population in Lake Beyşehir. Turk J Zool 23, 189–194.

Barnabé, G., 1980. Exposé synoptique des données biologiques sur le loup ou bar, Dicentrarchus labrax (Linné, 1758). Synopsis FAO sur les pêches 70.

Benzer, S.Ş., Gül, A., Yilmaz, M., 2007. The feeding biology of Tinca tinca L., 1758 living in Hirfanlı Dam Lake. Fen Bilimleri Dergisi 28.

Bergersen, R., 1996. Sticklebacks from Greenland. Journal of Fish Biology 48, 799–801. https ://doi.org/10.1111/j.1095-8649.1996.tb01474.x

Bergman, E., 1991. Changes in Abundance of Two Percids, Perca fluviatilis and Gymnocephalus cernuus, along a Productivity Gradient : Relations to Feeding Strategies and Competitive Abilities. Can. J. Fish. Aquat. Sci. 48, 536–545.

https ://doi.org/10.1139/f91-068

Bergman, E., Greenberg, L.A., 1994. Competition between a Planktivore, a Benthivore, and a Species with Ontogenetic Diet Shifts. Ecology 75, 1233–1245. https ://doi.org/10.2307/1937449

Bischoff, A., Freyhof, J., 1998. Seasonal shifts in day-time resource use of 0+ barbel, Barbus barbus, in : Copp, G.H., Kováč, V., Hensel, K. (Eds.), When Do Fishes Become Juveniles ?, Developments in Environmental Biology of Fishes. Springer Netherlands, pp. 199–212. https ://doi.org/10.1007/978-94-017-3678-7_14

Blake, R.W., 2004. Fish functional design and swimming performance. Journal of Fish Biology 65, 1193–1222. https ://doi.org/10.1111/j.0022-1112.2004.00568.x

Blanco, S., Romo, S., Villena, M.-J., 2004. Experimental Study on the Diet of Mosquitofish (Gambusia holbrooki) under Different Ecological Conditions in a Shallow Lake. International Review of Hydrobiology 89, 250–262. https ://doi.org/10.1002/iroh.200310684

Bo, T., Cammarata, M., Candiotto, A., Fenoglio, S., 2012. Trophic preferences of three allochthonous fishes in Bormida River (Alessandria, NW Italy). Hidrobiológica 22, 195–200.

Boët, P., 1981. Eléments d'écologie du poisson-chat, Ictalurus melas (Rafinesque, 1820), du lac de Créteil : structure et dynamique de la population exploitation des ressources alimentaires et production.

Bohl, E., 1979. Diel pattern of pelagic distribution and feeding in planktivorous fish. Oecologia 44, 368–375. https ://doi.org/10.1007/BF00545241

Borme, D., Tirelli, V., Brandt, S.B., Umani, S.F., Arneri, E., 2009. Diet of Engraulis encrasicolus in the northern Adriatic Sea (Mediterranean) : ontogenetic changes and feeding selectivity. Marine Ecology Progress Series 392, 193–209.

Braber, L., de Groot, S.J., 1973. The food of five flatfish species (Pleuronectiformes) in the southern north sea. Netherlands Journal of Sea Research 6, 163–172. https ://doi.org/10.1016/0077-7579(73)90011-2

Cabral, H.N., 2000. Comparative feeding ecology of sympatric Solea solea and S. senegalensis, within the nursery areas of the Tagus estuary, Portugal. Journal of Fish Biology 57, 1550–1562.

Cabral, H.N., Ohmert, B., 2001. Diet of juvenile meagre, Argyrosomus regius, within the Tagus estuary. Cahiers de biologie marine 42, 289–293.

Cabral, J.A., Mieiro, C.L., Marques, J.C., 1998. Environmental and biological factors influence the relationship between a predator fish, Gambusia holbrooki, and its main prey in rice fields of the Lower Mondego River Valley (Portugal). Hydrobiologia 382, 41–51. https ://doi.org/10.1023/A :1003480920168

Cambrony, M., 1983. Recrutement et biologie des stades juveniles de Mugilidae (Poissons-Teleosteens) dans trois milieux lagunaires du Roussillon et du Narbonnais (Salses-Leucate, Lapalme, Bourdigou). Université de Perpignan, Perpignan, France.

Campbell, R.N.B., 1992. Food of an introduced population of pikeperch, Stizostedion lucioperca L., in Lake Egirdir, Turkey. Aquaculture Research 23, 71–85. https ://doi.org/10.1111/j.1365-2109.1992.tb00597.x

Cardona, L., 2001. Non-competitive coexistence between Mediterranean grey mullet : evidence from seasonal changes in food availability, niche breadth and trophic overlap. Journal of Fish Biology 59, 729–744. https ://doi.org/10.1111/j.1095-8649.2001.tb02376.x

Carol, J., Benejam, L., Benito, J., García-Berthou, E., 2009. Growth and diet of European catfish (Silurus glanis) in early and late invasion stages. Fundamental and Applied Limnology / Archiv f ? ?r Hydrobiologie 174, 317–328. https ://doi.org/10.1127/1863-9135/2009/0174-0317

Cherghou, S., Khodari, M., Yaâkoubi, F., Benabid, M., Badri, A., 2002. Contribution à l'étude du régime alimentairedu barbeau (Barbus barbus callensis Valenciennes, 1842) d'un cours d'eau du Moyen-Atlas (Maroc) : Oued Boufekrane.

Revue des sciences de l'eau/Journal of Water Science 15, 153–163.

Comte, L., Cucherousset, J., Boulêtreau, S., Olden, J.D., 2016. Resource partitioning and functional diversity of worldwide freshwater fish communities. Ecosphere 7, n/a-n/a. https ://doi.org/10.1002/ecs2.1356

Copp, G.H., Robert Britton, J., Cucherousset, J., García-Berthou, E., Kirk, R., Peeler, E., Stakénas, S., 2009. Voracious invader or benign feline ? A review of the environmental biology of European catfish Silurus glanis in its native and introduced ranges*. Fish and Fisheries 10, 252–282. https ://doi.org/10.1111/j.1467-2979.2008.00321.x

Costa, M.J., 1988. Écologie alimentaire des poissons de l'estuaire du Tage. Cybium 12, 301–320.

CTGREF, 1979. Étude halieutique de l'estuaire de la Gironde. CTGREF. Division Aménagements littoraux et aquaculture, Bordeaux.

Declerck, S., Louette, G., De Bie, T., De Meester, L., 2002. Patterns of diet overlap between populations of non-indigenous and native fishes in shallow ponds. Journal of Fish Biology 61, 1182–1197. https ://doi.org/10.1111/j.1095-8649.2002.tb02464.x

Dolbeth, M., Martinho, F., Leitão, R., Cabral, H., Pardal, M.A., 2008. Feeding patterns of the dominant benthic and demersal fish community in a temperate estuary. Journal of Fish Biology 72, 2500–2517. https ://doi.org/10.1111/j.1095-8649.2008.01856.x

Doornbos, G., Twisk, F., 1987. Density, growth and annual food consumption of gobiid fish in the saline Lake Grevelingen, The Netherlands. Netherlands Journal of Sea Research 21, 45–74. https ://doi.org/10.1016/0077-7579(87)90022-6

Dorman, J.A., 1991. Investigations into the biology of the garfish, Belone belone (L.), in Swedish waters. Journal of Fish Biology 39, 59–69. https ://doi.org/10.1111/j.1095-8649.1991.tb04341.x

Dorman, J.A., 1988. Diet of the garfish, Belone belone (L.), from Courtmacsherry Bay, Ireland. Journal of Fish Biology 33, 339–346. https ://doi.org/10.1111/j.1095-8649.1988.tb05476.x

Dukowska, M., Grzybkowska, M., Marszał, L., Zięba, G., 2009. The food preferences of three-spined stickleback, Gasterosteus aculeatus L., downstream from a dam reservoir. Oceanological and Hydrobiological Studies 38, 39–50.

Dumay, O., 2003. Étude de la diversité des traits fonctionnels des différentes communautés ichtyologiques en milieu lagunaire.

Dumay, O., Tari, P.S., Tomasini, J.A., Mouillot, D., 2004. Functional groups of lagoon fish species in Languedoc Roussillon, southern France. Journal of Fish Biology 64, 970–983. https ://doi.org/10.1111/j.1095-8649.2004.00365.x

Erguden, S.A., 2013. Age, growth, sex ratio and diet of eastern mosquitofish Gambusia holbrooki Girard, 1859 in Seyhan Dam Lake (Adana/Turkey). Iranian Journal of Fisheries Sciences 12, 204–218.

Ferrari, I., Chieregato, A.R., 1981. Feeding habits of juvenile stages of Sparus auratus L., Dicentrarchus labrax L. and Mugilidae in a brackish embayment of the Po River delta. Aquaculture 25, 243–257.

Fonseca, L., Colclough, S., Hughes, R.G., 2011. Variations in the feeding of 0-group bass Dicentrarchus labrax (L.) in managed realignment areas and saltmarshes in SE England. Hydrobiologia 672, 15–31. https ://doi.org/10.1007/s10750-011-0753-x

Foster, S.J., Vincent, A.C.J., 2004. Life history and ecology of seahorses : implications for conservation and management. Journal of Fish Biology 65, 1–61. https ://doi.org/10.1111/j.0022-1112.2004.00429.x

Froese, R., Pauly, D., 2014a. Syngnathus rostellatus (Nilsson, 1855) [WWW Document]. FishBase. URL http ://www.fishbase.org (accessed 3.14.17).

Froese, R., Pauly, D., 2014b. Umbrina canariensis (Valenciennes, 1843) [WWW Document]. FishBase. URL http ://www.fishbase.org (accessed 3.14.17).

Froese, R., Pauly, D., 2014c. Umbrina cirrosa (Linnaeus, 1758) [WWW Document]. FishBase. URL

http ://www.fishbase.org (accessed 3.14.17).

Funk, J.L., Cleland, E.E., Suding, K.N., Zavaleta, E.S., 2008. Restoration through reassembly : plant traits and invasion resistance. Trends in Ecology & Evolution 23, 695–703. https ://doi.org/10.1016/j.tree.2008.07.013

García-Berthou, E., 2001. Size- and depth-dependent variation in habitat and diet of the common carp (Cyprinus carpio). Aquat. sci. 63, 466–476. https ://doi.org/10.1007/s00027-001-8045-6

García-Berthou, E., 1999. Food of introduced mosquitofish : ontogenetic diet shift and prey selection. Journal of Fish Biology 55, 135–147. https ://doi.org/10.1111/j.1095-8649.1999.tb00663.x

García-Berthou, E., Moreno-Amich, R., 2000. Food of introduced pumpkinseed sunfish : ontogenetic diet shift and seasonal variation. Journal of Fish Biology 57, 29–40. https ://doi.org/10.1111/j.1095-8649.2000.tb00773.x

Garcia-Franquesa, E., Molinero, A., Valero, J., Flos, R., 1996. Influence of sex, age and season on the feeding habits of the flatfish Solea senegalensis. Environ Biol Fish 47, 289–298. https ://doi.org/10.1007/BF00000501

Garnås, E., Hvidsten, N.A., 1986. The food of Atlantic salmon Salmo salar L. and brown trout Salmo trutta L. smolts during migration in the Orkla River, Norway. Fauna Norvegica, Series A 6, 24–28.

Gatz, A.J., 1979. Community organization in fishes as indicated by morphological features. Ecology 60, 711–718.

Giles, N., Street, M., Wright, R.M., 1990. Diet composition and prey preference of tench, Tinca tinca (L.), common bream, Abramis brama (L.), perch, Perca fluviatilis L. and roach, Rutilus rutilus (L.), in two contrasting gravel pit lakes : potential trophic overlap with wildfowl. Journal of fish biology 37, 945–957.

Ginter, K., Kangur, K., Kangur, A., Kangur, P., Haldna, M., 2011. Diet patterns and ontogenetic diet shift of pikeperch, Sander lucioperca (L.) fry in lakes Peipsi and Võrtsjärv (Estonia). Hydrobiologia 660, 79–91. https ://doi.org/10.1007/s10750-010-0393-6

Girardin, M., Castelnaud, G., 2013. Surveillance halieutique de l'estuaire de la Gironde : suivi des captures 2012, étude de la faune circulante 2012. Irstea.

Gisbert, E., Cardona, L., Castelló, F., 1996. Resource partitioning among planktivorous fish larvae and fry in a Mediterranean coastal lagoon. Estuarine, Coastal and Shelf Science 43, 723–735.

Gkenas, C., Oikonomou, A., Economou, A., Kiosse, F., Leonardos, I., others, 2012. Life history pattern and feeding habits of the invasive mosquitofish, Gambusia holbrooki, in Lake Pamvotis (NW Greece). Journal of Biological Research-Thessaloniki 17, 121–36.

Godinho, F., Ferreira, M.T., Cortes, R.V., 1997. The environmental basis of diet variation in pumpkinseed sunfish, Lepomis gibbosus, and largemouth bass, Micropterus salmoides, along an Iberian river basin. Environmental Biology of Fishes 50, 105–115. https ://doi.org/10.1023/A :1007302718072

Gozlan, R.E., Andreou, D., Asaeda, T., Beyer, K., Bouhadad, R., Burnard, D., Caiola, N., Cakic, P., Djikanovic, V., Esmaeili, H.R., others, 2010. Pan-continental invasion of Pseudorasbora parva : towards a better understanding of freshwater fish invasions. Fish and Fisheries 11, 315–340.

Green, B.C., Smith, D.J., Grey, J., Underwood, G.J.C., 2012. High site fidelity and low site connectivity in temperate salt marsh fish populations : A stable isotope approach. Oecologia 168, 245–255.

Gushchin, A.V., 2013. Feeding of fish young from littoral of the gulf Arguin (Mauritania). J. Ichthyol. 53, 731–738. https ://doi.org/10.1134/S0032945213050068

Hamerlynck, O., Cattrijsse, A., 1994. The food of Pomatoschistus minutus (Pisces, Gobiidae) in Belgian coastal waters, and a comparison with the food of its potential competitor P. lozanoi. Journal of Fish Biology 44, 753–771. https ://doi.org/10.1111/j.1095-8649.1994.tb01253.x

Hampel, H., Cattrijsse, A., 2004. Temporal variation in feeding rhythms in a tidal marsh population of the common

goby Pomatoschistus microps (Kroyer, 1838). Aquat. Sci. 66, 315–326. https ://doi.org/10.1007/s00027-004-0682-0

Hampel, H., Cattrijsse, A., Elliott, M., 2005. Feeding habits of young predatory fishes in marsh creeks situated along the salinity gradient of the Schelde estuary, Belgium and The Netherlands. Helgoland Marine Research 59, 151–162. https ://doi.org/10.1007/s10152-004-0214-2

Hansson, S., Arrhenius, F., Nellbring, S., 1997. Diet and growth of pikeperch (Stizostedion lucioperca L.) in a Baltic Sea area. Fisheries Research 31, 163–167. https ://doi.org/10.1016/S0165-7836(97)00022-2

Haugland, M., Holst, J.C., Holm, M., Hansen, L.P., 2006. Feeding of Atlantic salmon (Salmo salar L.) post-smolts in the Northeast Atlantic. ICES Journal of Marine Science : Journal du Conseil 63, 1488–1500.

Hellawell, J.M., 1971. The autecology of the chub, Squalius cephalus (L.), of the River Lugg and the Afon Llynfi. Freshwater Biology 1, 29–60. https ://doi.org/10.1111/j.1365-2427.1971.tb01545.x

Hjelm, J., van de Weerd, G.H., Sibbing, F.A., 2003. Functional link between foraging performance, functional morphology, and diet shift in roach (Rutilus rutilus). Can. J. Fish. Aquat. Sci. 60, 700–709. https ://doi.org/10.1139/f03-058

Holčík, J., 1989. The freshwater fishes of Europe, VOL 1/II.

Holden, M.J., Tucker, R.N., 1974. The food of Raja clavata Linnaeus 1758, Raja montagui Fowler 1910, Raja naevus Müller and Henle 1841 and Raja brachyura Lafont 1873 in British waters. Journal du Conseil 35, 189–193.

Hölker, F., Thiel, R., 1998. Biology of Ruffe (Gymnocephalus cernuus (L.)) - A Review of Selected Aspects from European Literature. Journal of Great Lakes Research 24, 186–204. https ://doi.org/10.1016/S0380-1330(98)70812-3

Horppila, J., Nurminen, L., 2009. Food niche segregation between two herbivorous cyprinid species in a turbid lake. Journal of Fish Biology 75, 1230–1243. https ://doi.org/10.1111/j.1095-8649.2009.02359.x

Hostens, K., Mees, J., 1999. The mysid-feeding guild of demersal fishes in the brackish zone of the Westerschelde estuary. Journal of Fish Biology 55, 704–719.

Hynes, H.B.N., 1950. The food of fresh-water sticklebacks (Gasterosteus aculeatus and Pygosteus pungitius), with a review of methods used in studies of the food of fishes. The Journal of Animal Ecology 36–58.

Iglésias, S., 2013. Actinopterygians from the North-eastern Atlantic and the Mediterranean (A natural classification based on collection specimens). Volume I (plates). Provisional version 09. SP Iglésias.

Jamet, J.-L., 1994. Feeding activity of adult roach (Rutilus rutilus (L.)), perch (Perca fluviatilis L.) and ruffe (Gymnocephalus cernuus (L.)) in eutrophic Lake Aydat (France). Aquatic Science 56, 376–387. https ://doi.org/10.1007/BF00877183

Jutila, E., Jokikokko, E., 2008. Seasonal differences in smolt traits and post-smolt survival of wild Atlantic salmon, Salmo salar, migrating from a northern boreal river. Fisheries Management and Ecology 15, 1–9. https ://doi.org/10.1111/j.1365-2400.2007.00562.x

Kakareko, T., 2001. The diet, growth and condition of common bream, Abramis brama [L.] in Wloclawek Reservoir. Acta Ichthyologica et Piscatoria 31.

Kangur, K., Kangur, A., Kangur, P., 1999. A comparative study on the feeding of eel, Anguilla anguilla (L.), bream, Abramis brama (L.) and ruffe, Gymnocephalus cernuus (L.) in Lake Võrtsjärv, Estonia, in : Walz, N., Nixdorf, B. (Eds.), Shallow Lakes '98, Developments in Hydrobiology. Springer Netherlands, pp. 65–72. https ://doi.org/10.1007/978-94-017-2986-4_7

Kapuscinski, K.L., Farrell, J.M., Wilkinson, M.A., 2012. Feeding patterns and population structure of an invasive cyprinid, the rudd Scardinius erythrophthalmus (Cypriniformes, Cyprinidae), in Buffalo Harbor (Lake Erie) and the upper Niagara River. Hydrobiologia 693, 169–181. https ://doi.org/10.1007/s10750-012-1106-0

Keast, A., Webb, D., 1966. Mouth and body form relative to feeding ecology in the fish fauna of a small lake, Lake

Opinicon, Ontario. Journal of the Fisheries Board of Canada 23, 1845–1874.

Keith, P., Persat, H., Feunteun, E., Allardi, J., 2011. Les poissons d'eau douce de France, Biotope, Mèze, Inventaires et biodiversité. Muséum national d'histoire naturelle, Paris.

Kellnreitner, F., Pockberger, M., Asmus, H., 2012. Seasonal variation of assemblage and feeding guild structure of fish species in a boreal tidal basin. Estuarine, Coastal and Shelf Science 108, 97–108.

Kendrick, A.J., Hyndes, G.A., 2005. Variations in the dietary compositions of morphologically diverse syngnathid fishes. Environ Biol Fish 72, 415–427. https ://doi.org/10.1007/s10641-004-2597-y

Kennedy, M., Fitzmaurice, P., 1970. The Biology of the Tench Tinca tinca (L.) in Irish Waters. Proceedings of the Royal Irish Academy. Section B : Biological, Geological, and Chemical Science 69, 31–82.

Khan, T.A., 2003. Dietary studies on exotic carp (Cyprinus carpio L.) from two lakes of western Victoria, Australia. Aquat. Sci. 65, 272–286. https ://doi.org/10.1007/s00027-003-0658-5

Kitsos, M.-S., Tzomos, T., Anagnostopoulou, L., Koukouras, A., 2008. Diet composition of the seahorses, Hippocampus guttulatus Cuvier, 1829 and Hippocampus hippocampus (L., 1758) (Teleostei, Syngnathidae) in the Aegean Sea. Journal of Fish Biology 72, 1259–1267. https ://doi.org/10.1111/j.1095-8649.2007.01789.x

La Mesa, M., Arneri, E., Caputo, V., Iglesias, M., 2005. The Transparent Goby, Aphia MinutaReview of Biology and Fisheries of a Paedomorphic European Fish. Reviews in Fish Biology and Fisheries 15, 89–109.

Laffaille, P., Feunteun, E., Lefebvre, C., Radureau, A., Sagan, G., Lefeuvre, J.-C., 2002. Can thin-lipped mullet directly exploit the primary and detritic production of European macrotidal salt marshes ? Estuarine, Coastal and Shelf Science 54, 729–736.

Laffaille, P., Lefeuvre, J.-C., Schricke, M.-T., Feunteun, E., 2001. Feeding ecology of o-group sea bass, Dicentrarchus labrax, in salt marshes of Mont Saint Michel Bay (France). Estuaries 24, 116–125. https ://doi.org/10.2307/1352818

Lanoiselée, C., 2004. Diversité fonctionnelle des communautés de poissons de plans d'eau (Rapport de stage de Master 2). Université Paul Sabatier, Toulouse.

Lappalainen, A., Westerbom, M., Vesala, S., 2004. Blue mussels (Mytilus edulis) in the diet of roach (Rutilus rutilus) in outer archipelago areas of the western Gulf of Finland, Baltic Sea, in : Biology of the Baltic Sea. Springer, Dordrecht, pp. 87–92. https ://doi.org/10.1007/978-94-017-0920-0_8

Lecomte-Finiger, R., 1983. Régime alimentaire des civelles et anguillettes (Anguilla anguilla) dans trois étangs saumâtres du Roussillon. Bulletin d'Écologie 14, 297–306.

Legendre, P., Legendre, L., 1998. Numerical ecology : second English edition. Developments in environmental modelling 20.

Leunda, P.M., Oscoz, J., Elvira, B., Agorreta, A., Perea, S., Miranda, R., 2008. Feeding habits of the exotic black bullhead Ameiurus melas (Rafinesque) in the Iberian Peninsula : first evidence of direct predation on native fish species. Journal of Fish Biology 73, 96–114.

Lorenzoni, M., Carosi, A., Pedicillo, G., Trusso, A., 2007. A comparative study on the feeding competition of the European perch Perca fluviatilis L. and the ruffe Gymnocephalus cernuus (L.) in Lake Piediluco (Umbria, Italy). Bulletin Francais de la Peche et de la Pisciculture 35–57.

Maazouzi, C., Médoc, V., Pihan, J.-C., Masson, G., 2011. Size-related dietary changes observed in young-of-the-year pumpkinseed (Lepomis gibbosus) : stomach contents and fatty acid analyses. Aquat Ecol 45, 23–33. https ://doi.org/10.1007/s10452-010-9320-1

Magnhagen, C., Wiederholm, A.-M., 1982. Food selectivity versus prey availability : a study using the marine fish Pomatoschistus microps. Oecologia 55, 311–315. https ://doi.org/10.1007/BF00376917

Mahon, R., 1984. Divergent structure in fish taxocenes of north temperate streams. Canadian Journal of Fisheries and Aquatic Sciences 41, 330–350.

Mann, R.H.K., 1976. Observations on the age, growth, reproduction and food of the chub Squalius cephalus (L.) in the River Stour, Dorset. Journal of Fish Biology 8, 265–288. https ://doi.org/10.1111/j.1095-8649.1976.tb03950.x

Mansfield, S., Mcardle, B.H., 1998. Dietary composition of Gambusia affinis (Family Poeciliidae) populations in the northern Waikato region of New Zealand. New Zealand Journal of Marine and Freshwater Research 32, 375–383. https ://doi.org/10.1080/00288330.1998.9516832

Martinho, F., Leitão, R., Neto, J.M., Cabral, H., Lagardère, F., Pardal, M.A., 2008. Estuarine colonization, population structure and nursery functioning for 0-group sea bass (Dicentrarchus labrax), flounder (Platichthys flesus) and sole (Solea solea) in a mesotidal temperate estuary. Journal of Applied Ichthyology 24, 229–237. https ://doi.org/10.1111/j.1439-0426.2007.01049.x

Martyniak, A., Kucharczyk, D., Hliwa, P., Boron, S., Szymanska, U., Czerkies, P., 1999. Diet of white bream, Blicca bjoerkna (L.), from Lake Wirbel. Polish Archives of Hydrobiology.

Matić-Skoko, S., Ferri, J., Tutman, P., Skaramuca, D., DJikić, D., Lisičić, D., Franić, Z., Skaramuca, B., 2012. The age, growth and feeding habits of the European conger eel, Conger conger (L.) in the Adriatic Sea. Marine Biology Research 8, 1012–1018.

McIntyre, J.K., Beauchamp, D.A., Mazur, M.M., Overman, N.C., 2006. Ontogenetic Trophic Interactions and Benthopelagic Coupling in Lake Washington : Evidence from Stable Isotopes and Diet Analysis. Transactions of the American Fisheries Society 135, 1312–1328. https ://doi.org/10.1577/T05-099.1

Mendes, C., Ramos, S., Bordalo, A.A., 2014. Feeding ecology of juvenile flounder Platichthys flesus in an estuarine nursery habitat : Influence of prey–predator interactions. Journal of Experimental Marine Biology and Ecology 461, 458–468. https ://doi.org/10.1016/j.jembe.2014.09.016

Michel, P., Oberdorff, T., 1995. Feeding habits of fourteen European freshwater fish species. Cybium 19, 5–46.

Moore, J.W., Moore, I.A., 1976. The basis of food selection in some estuarine fishes. Eels, Anguilla anguilla (L.), whiting, Merlangius merlangus (L.), sprat, Sprattus sprattus (L.) and stickleback, Gasterosteus aculeatus L. Journal of Fish Biology 9, 375–390. https ://doi.org/10.1111/j.1095-8649.1976.tb04686.x

Morato, T., Solà, E., Grós, M.P., Menezes, G.M., 1999. Diets of forkbeard (Phycis phycis) and conger eel (Conger conger) off the Azores during spring of 1996 and 1997. Dietas da abrótea (Phycis phycis) e do congro (Conger conger) dos Açores durante a primavera de 1996 e 1997.

Morote, E., Olivar, M.P., Villate, F., Uriarte, I., 2010. A comparison of anchovy (Engraulis encrasicolus) and sardine (Sardina pilchardus) larvae feeding in the Northwest Mediterranean : influence of prey availability and ontogeny. ICES J Mar Sci 67, 897–908. https ://doi.org/10.1093/icesjms/fsp302

Nakamura, I., 1985. FAO species catalogue. v. 5 : Billfishes of the world. An annotated and illustrated catalogue of marlins, sailfishes, spearfishes and swordfishes known to date. FAO Fisheries Synopsis (FAO).

Nikolski, G.V., 1933. On the influence of the rate of flow on the fish fauna of the rivers of central Asia. The Journal of Animal Ecology 266–281.

Niksirat, H., Hatef, A., Abdoli, A., 2010. Life cycle and feeding habits of the threespined stickleback Gasterosteus aculeatus (Linnaeus, 1758) : an alien species in the southeast Caspian Sea. International Aquatic Research 2, 97–104.

Nilsson, P.A., Brönmark, C., Pettersson, L.B., 1995. Benefits of a predtor-induced morphology in crucian carp. Oecologia 104, 291–296. https ://doi.org/10.1007/BF00328363

Nissling, A., Jacobsson, M., Hallberg, N., 2007. Feeding ecology of juvenile turbot Scophthalmus maxi-

mus and flounder Pleuronectes flesus at Gotland, Central Baltic Sea. Journal of Fish Biology 70, 1877–1897. https ://doi.org/10.1111/j.1095-8649.2007.01463.x

Nunn, A.D., Harvey, J.P., Cowx, I.G., 2007. The food and feeding relationships of larval and 0+ year juvenile fishes in lowland rivers and connected waterbodies. I. Ontogenetic shifts and interspecific diet similarity. Journal of Fish Biology 70, 726–742. https ://doi.org/10.1111/j.1095-8649.2007.01334.x

Nunn, A.D., Tewson, L.H., Cowx, I.G., 2012. The foraging ecology of larval and juvenile fishes. Rev Fish Biol Fisheries 22, 377–408. https ://doi.org/10.1007/s11160-011-9240-8

Nurminen, L., Pekcan-Hekim, Z., Horppila, J., 2010. Feeding efficiency of planktivorous perch Perca fluviatilis and roach Rutilus rutilus in varying turbidity : an individual-based approach. Journal of Fish Biology 76, 1848–1855. https ://doi.org/10.1111/j.1095-8649.2010.02600.x

Ogle, D.H., Ray, B.A., Brown, W.P., 2004. Diet of Larval Ruffe (Gymnocephalus cernuus) in the St. Louis River Harbor, Lake Superior. Journal of Great Lakes Research 30, 287–292. https ://doi.org/10.1016/S0380-1330(04)70346-9

O'Sullivan, S., Moriarty, C., Davenport, J., 2004. Analysis of the stomach contents of the European conger eel Conger conger in Irish waters. Journal of the Marine Biological Association of the UK 84, 823–826.

Park, B.K., Park, S.S., 2005. Effects of stream hydraulic conditions on foraging strategies of false dace, Pseudorasbora parva, in the lentic ecosystem. Journal of environmental biology/Academy of Environmental Biology, India 26, 635–643.

Pasquaud, S., 2006. Les relations trophiques : éléments de structuration des peuplements ichtyologiques en milieu estuarien. Application à l'estuaire de la Gironde. Université Bordeaux I, Ecole Doctorale Siences du Vivant, Géosciences, Sciences de l'Environnement.

Pasquaud, S., Elie, P., Jeantet, C., Billy, I., Martinez, P., Girardin, M., 2008. A preliminary investigation of the fish food web in the Gironde estuary, France, using dietary and stable isotope analyses. Estuarine, Coastal and Shelf Science 78, 267–279. https ://doi.org/10.1016/j.ecss.2007.12.014

Pasquaud, S., Pillet, M., David, V., Sautour, B., Elie, P., 2010. Determination of fish trophic levels in an estuarine system. Estuarine, Coastal and Shelf Science 86, 237–246. https ://doi.org/10.1016/j.ecss.2009.11.019

Peltonen, H., Vinni, M., Lappalainen, A., Pönni, J., 2004. Spatial feeding patterns of herring (Clupea harengus L.), sprat (Sprattus sprattus L.), and the three-spined stickleback (Gasterosteus aculeatus L.) in the Gulf of Finland, Baltic Sea. ICES J Mar Sci 61, 966–971. https ://doi.org/10.1016/j.icesjms.2004.06.008

Pérez-Bote, J.L., Roso, R., 2012. Diet of the introduced pikeperch Sander lucioperca (L.) (Osteichthyes, Percidae) in a recent colonised reservoir in south-western Iberian Peninsula. Italian Journal of Zoology 79, 617–626. https ://doi.org/10.1080/11250003.2012.687777

Persson, A., Brönmark, C., 2008. Pikeperch Sander lucioperca trapped between niches : foraging performance and prey selection in a piscivore on a planktivore diet. Journal of Fish Biology 73, 793–808. https ://doi.org/10.1111/j.1095-8649.2008.01956.x

Persson, A., Brönmark, C., 2002. Foraging capacities and effects of competitive release on ontogenetic diet shift in bream, Abramis brama. Oikos 97, 271–281.

Persson, L., 1983. Food consumption and the significance of detritus and algae to intraspecific competition in roach Rutilus rutilus in a shallow eutrophic lake. Oikos 118–125.

Peterka, J., Matína, J., Lipka, J., 2003. The diet and growth of larval and juvenile pikeperch (Stizostedion lucioperca (L.)) : A comparative study of fishponds and a reservoir. Aquaculture International 11, 337–348. https ://doi.org/10.1023/A :1025791208123

Petridis, D., 1990. The influence of grass carp on habitat structure and its subsequent effect on the diet of tench. Journal of Fish Biology 36, 533–544. https ://doi.org/10.1111/j.1095-8649.1990.tb03555.x

Piet, G.J., 1998. Ecomorphology of a size-structured tropical freshwater fish community. Environmental Biology of Fishes 51, 67–86. https ://doi.org/10.1023/A :1007338532482

Pihl, L., 1985. Food selection and consumption of mobile epibenthic fauna in shallow marine areas. Marine Ecology Progress Series 22, 169–179.

Piria, M., Treer, T., Aničić, I., Safner, R., Odak, T., 2005. The natural diet of five cyprinid fish species. Agriculturae Conspectus Scientificus (ACS) 70, 21–28.

Podani, J., 1999. Extending Gower's general coefficient of similarity to ordinal characters. Taxon 331–340.

Popova, O.A., Sytina, L.A., 1977. Food and Feeding Relations of Eurasian Perch (Perca fluviatilis) and Pikeperch (Stizostedion lucioperca) in Various Waters of the USSR. J. Fish. Res. Bd. Can. 34, 1559–1570. https ://doi.org/10.1139/f77-219

Prejs, A., 1984. Herbivory by temperate freshwater fishes and its consequences. Environmental Biology of Fishes 10, 281–296. https ://doi.org/10.1007/BF00001481

Pyke, G.H., 2005. A Review of the Biology of Gambusia affinis and G. holbrooki. Rev Fish Biol Fisheries 15, 339–365. https ://doi.org/10.1007/s11160-006-6394-x

Rezsu, E., Specziár, A., 2006. Ontogenetic diet profiles and size-dependent diet partitioning of ruffe Gymnocephalus cernuus, perch Perca fluviatilis and pumpkinseed Lepomis gibbosus in Lake Balaton. Ecology of Freshwater Fish 15, 339–349. https ://doi.org/10.1111/j.1600-0633.2006.00172.x

Russo, T., Costa, C., Cataudella, S., 2007. Correspondence between shape and feeding habit changes throughout ontogeny of gilthead sea bream Sparus aurata L., 1758. Journal of Fish Biology 71, 629–656. https ://doi.org/10.1111/j.1095-8649.2007.01528.x

Salgado, J.P., Cabral, H.N., Costa, M.J., 2004. Feeding ecology of the gobies Pomatoschistus minutes (Pallas, 1770) and Pomatoschistus microps (Krøyer, 1838) in the upper Tagus estuary, Portugal. Scientia Marina 68, 425–434. https ://doi.org/10.3989/scimar.2004.68n3425

Sánchez-Gonzáles, S., Ruiz-Campos, G., Contreras-Balderas, S., 2001. Feeding ecology and habitat of the threespine stickleback, Gasterosteus aculeatus microcephalus, in a remnant population of northwestern Baja California, México. Ecology of Freshwater Fish 10, 191–197. https ://doi.org/10.1034/j.1600-0633.2001.100401.x

Schleuter, D., Daufresne, M., Veslot, J., Mason, N.W.H., Lanoiselée, C., Brosse, S., Beauchard, O., Argillier, C., 2012. Geographic isolation and climate govern the functional diversity of native fish communities in European drainage basins. Global Ecology and Biogeography 21, 1083–1095. https ://doi.org/10.1111/j.1466-8238.2012.00763.x

Schleuter, D., Eckmann, R., 2008. Generalist versus specialist : the performances of perch and ruffe in a lake of low productivity. Ecology of Freshwater Fish 17, 86–99. https ://doi.org/10.1111/j.1600-0633.2007.00262.x

Selleslagh, J., Blanchet, H., Bachelet, G., Lobry, J., 2015. Feeding Habitats, Connectivity and Origin of Organic Matter Supporting Fish Populations in an Estuary with a Reduced Intertidal Area Assessed by Stable Isotope Analysis. Estuaries and Coasts 38, 1431–1447. https ://doi.org/10.1007/s12237-014-9911-5

Selleslagh, J., Echard, A., Pecheyran, C., Baudrimont, M., Lobry, J., Daverat, F., 2016. Can analysis of Platichthys flesus otoliths provide relevant data on historical metal pollution in estuaries ? Experimental and in situ approaches. Sci. Total Environ. 557–558, 20–30. https ://doi.org/10.1016/j.scitotenv.2016.03.014

Sever, T.M., Bayhan, B., Bilge, G., Taşkavak, E., 2009. Diet composition of Belone belone (Linnaeus, 1761) (Pisces : Belonidae) in the Aegean Sea. Journal of Applied Ichthyology 25, 702–706. https ://doi.org/10.1111/j.1439-

0426.2009.01368.x

Shoniya, L., Dzhaposhvili, B., Kokosadze, T., others, 2011. The invasive species Pseudorazbora parva (Teleostei, Cyprinidae) in the ecosystem of Lake Bazalety. Zoologichesky Zhunal 90, 1277–1280.

Shustov, Y.A., Belyakova, E.A., 2012. Comparative study on the feeding of parrs and smolts of the atlantic salmon (Salmo salar L.) in the subarctic Varzuga River basin. Russ J Ecol 43, 462–465. https ://doi.org/10.1134/S106741361205013X

Sibbing, F.A., Nagelkerke, L.A.J., 2001. Resource partitioning by Lake Tana barbs predicted from fish morphometrics and prey characteristics. Reviews in Fish Biology and Fisheries 10, 393–437. https ://doi.org/10.1023/A :1012270422092

Specziár, A., 2005. First Year Ontogenetic Diet Patterns in Two Coexisting Sander Species, S. lucioperca and S. volgensis in Lake Balaton. Hydrobiologia 549, 115–130. https ://doi.org/10.1007/s10750-005-5766-x

Specziár, A., 2004. Life history pattern and feeding ecology of the introduced eastern mosquitofish, Gambusia holbrooki, in a thermal spa under temperate climate, of Lake Hévíz, Hungary. Hydrobiologia 522, 249–260. https ://doi.org/10.1023/B :HYDR.0000029978.46013.d1

Specziár, A., Rezsu, E.T., 2009. Feeding guilds and food resource partitioning in a lake fish assemblage : an ontogenetic approach. Journal of Fish Biology 75, 247–267.

Specziár, A., Tölg, L., Bíró, P., 1997. Feeding strategy and growth of cyprinids in the littoral zone of Lake Balaton. Journal of Fish Biology 51, 1109–1124. https ://doi.org/10.1111/j.1095-8649.1997.tb01130.x

Spilseth, S.A., Simenstad, C.A., 2011. Seasonal, Diel, and Landscape Effects on Resource Partitioning between Juvenile Chinook Salmon (Oncorhynchus tshawytscha) and Threespine Stickleback (Gasterosteus aculeatus) in the Columbia River Estuary. Estuaries and Coasts 34, 159–171. https ://doi.org/10.1007/s12237-010-9349-3

Sutela, T., Hyvärinen, P., 2002. Diet and growth of stocked and wild 0+ pikeperch, Stizostedion lucioperca (L.). Fisheries Management and Ecology 9, 57–63. https ://doi.org/10.1046/j.1365-2400.2002.00251.x

Tancioni, L., Mariani, S., Maccaroni, A., Mariani, A., Massa, F., Scardi, M., Cataudella, S., 2003. Locality-specific variation in the feeding of Sparus aurata L. : evidence from two Mediterranean lagoon systems. Estuarine, Coastal and Shelf Science 57, 469–474. https ://doi.org/10.1016/S0272-7714(02)00376-1

Tarvainen, M., Vuorio, K., Sarvala, J., 2008. The diet of ruffe Gymnocephalus cernuus (L.) in northern lakes : new insights from stable isotope analyses. Journal of Fish Biology 72, 1720–1735. https ://doi.org/10.1111/j.1095-8649.2008.01847.x

Taverny, C., Elie, P., 2001. Régime alimentaire de la grande alose Alosa alosa (Linné, 1766) et de l'alose feinte Alosa fallax (Lacépède, 1803) dans le golfe de Gascogne. Bull. Fr. Pêche Piscic. 837–852. https ://doi.org/10.1051/kmae :2001022

Thiel, R., Mehner, T., Köpcke, B., Kafemann, R., 1996. Diet Niche Relationships among Early Life Stages of Fish in German Estuaries. Mar. Freshwater Res. 47, 123–136. https ://doi.org/10.1071/mf9960123

Thorstad, E.B., Whoriskey, F., Uglem, I., Moore, A., Rikardsen, A.H., Finstad, B., 2012. A critical life stage of the Atlantic salmon Salmo salar : behaviour and survival during the smolt and initial post-smolt migration. Journal of Fish Biology 81, 500–542. https ://doi.org/10.1111/j.1095-8649.2012.03370.x

Tičina, V., Vidjak, O., Kačič, I., 2000. Feeding of adult sprat, Sprattus sprattus, during spawning season in the Adriatic Sea. Italian journal of zoology 67, 307–311.

Tudela, S., Palomera, I., 1997. Trophic ecology of the European anchovy Engraulis encrasicolus in the Catalan Sea (northwest Mediterranean). Mar Ecol Prog Ser 160, 121–134. https ://doi.org/10.3354/meps160121

Ünver, B., Erk'akan, F., 2011. Diet composition of chub, Squalius cephalus (Teleostei : Cyprinidae), in Lake Tödürge,

Sivas, Turkey. Journal of Applied Ichthyology 27, 1350–1355. https ://doi.org/10.1111/j.1439-0426.2011.01766.x

Vandendriessche, S., Messiaen, M., O'Flynn, S., Vincx, M., Degraer, S., 2007. Hiding and feeding in floating seaweed : Floating seaweed clumps as possible refuges or feeding grounds for fishes. Estuarine, Coastal and Shelf Science 71, 691–703. https ://doi.org/10.1016/j.ecss.2006.09.017

Vašek, M., Kubečka, J., Matěna, J., Seďa, J., 2006. Distribution and Diet of 0+ Fish within a Canyon-Shaped European Reservoir in Late Summer. International Review of Hydrobiology 91, 178–194. https ://doi.org/10.1002/iroh.200510835

Vilizzi, L., Walker, K.F., 1998. The onset of the juvenile period in carp, Cyprinus carpio : a literature survey, in : Copp, G.H., Kováč, V., Hensel, K. (Eds.), When Do Fishes Become Juveniles ?, Developments in Environmental Biology of Fishes. Springer Netherlands, pp. 93–102. https ://doi.org/10.1007/978-94-017-3678-7_7

Villéger, S., 2008. Dynamique de la diversité fonctionnelle des communautés de poissons (lagune de Terminos, Mexique) (Thèse de 3ème cycle). Montpellier SupAgro, Montpellier.

Villéger, S., Miranda, J.R., Hernández, D.F., Mouillot, D., 2010. Contrasting changes in taxonomic vs. functional diversity of tropical fish communities after habitat degradation. Ecological Applications 20, 1512–1522. https ://doi.org/10.1890/09-1310.1

Violle, C., Navas, M.-L., Vile, D., Kazakou, E., Fortunel, C., Hummel, I., Garnier, E., 2007. Let the concept of trait be functional ! Oikos 116, 882–892. https ://doi.org/10.1111/j.0030-1299.2007.15559.x

Vøllestad, L.A., Varreng, K., Poleo, A.B.S., 2004. Body depth variation in crucian carp Carassius carassius : an experimental individual-based study. Ecology of Freshwater Fish 13, 197–202.

Webb, P.W., 1988. Simple Physical Principles and Vertebrate Aquatic Locomotion. Integrative and Comparative Biology 28, 709–725. https ://doi.org/10.1093/icb/28.2.709

Webb, P.W., 1984. Body form, locomotion and foraging in aquatic vertebrates. American Zoologist 24, 107–120.

Werner, M.G., Mehner, T., Schultz, H., 1996. Which Factors Influence the Diet Composition of Age-0 Ruffe (Gymnocephalus cernuus (L.)) in the Bautzen Reservoir (Saxony, Germany) ? Limnologica 26, 145–152.

Winemiller, K.O., 1991. Ecomorphological diversification in lowland freshwater fish assemblages from five biotic regions. Ecological Monographs 61, 343–365.

Wolfram-Wais, A., Wolfram, G., Auer, B., Mikschi, E., Hain, A., 1999. Feeding habits of two introduced fish species (Lepomis gibbosus, Pseudorasbora parva) in Neusiedler See (Austria), with special reference to chironomid larvae (Diptera : Chironomidae), in : Walz, N., Nixdorf, B. (Eds.), Shallow Lakes '98, Developments in Hydrobiology. Springer Netherlands, pp. 123–129. https ://doi.org/10.1007/978-94-017-2986-4_12

Xavier, J.C., Cherel, Y., Assis, C.A., Sendão, J., Borges, T.C., 2010. Feeding ecology of conger eels (Conger conger) in north-east Atlantic waters. Journal of the Marine Biological Association of the United Kingdom 90, 493–501.

Xie, S., Cui, Y., Zhang, T., Li, Z., 2000. Seasonal patterns in feeding ecology of three small fishes in the Biandantang Lake, China. Journal of Fish Biology 57, 867–880.

Xie, S., Li, Z., Cui, Y., Murphy, B.R., 2005. Distribution, Feeding and Body Condition of Four Small Fish Species in the Near-shore and Central Areas of Liangzi Lake, China. Environ Biol Fish 74, 379–387. https ://doi.org/10.1007/s10641-005-2925-x

Yağci, M.A., Alp, A., Yağci, A., Uysal, R., 2014. Diet and prey selection of pikeperch (Sander lucioperca Linnaeus, 1758) population in Lake Eğirdir (Turkey). Archives of Biological Sciences 66, 1515–1527.

Zahorcsak, P., Silvano, R.A.M., Sazima, I., 2000. Feeding biology of a guild of benthivorous fishes in a sandy shore on south-eastern Brazilian coast. Revista Brasileira de Biologia 60, 511–518.

Zapletal, T., Mares, J., Jurajda, P., Vseticková, L., 2014. The food of roach, Rutilus rutilus (Actinopterygii : Cypriniformes : Cyprinidae), in a biomanipulated water supply reservoir. Acta Ichthyologica et Piscatoria 44, 15.

Zapletal, T., Mareš, J., Jurajda, P., Všetičková, L., others, 2012. The food of common bream (Abramis brama L.) in a biomanipulated water supply reservoir. Acta Universitatis Agriculturae et Silviculturae Mendelianae Brunensis 60, 45.

Zhao, T., Villéger, S., Lek, S., Cucherousset, J., 2014. High intraspecific variability in the functional niche of a predator is associated with ontogenetic shift and individual specialization. Ecology and Evolution 4, 4649. https ://doi.org/10.1002/ece3.1260

Zupo, V., Stübing, D., 2010. Diet of fish populations in posidonia oceanica meadows off the Island of Ischia (Gulf of Naples, Italy). Natural Science 2, 1274.
