# Supplementary material for: Change in fish functional diversity and assembly rules in the course of tidal marsh restoration
Source: PLoS One. 2018 Dec 19;13(12):e0209025. doi: 10.1371/journal.pone.0209025 (PMC6300267; doi:10.1371/journal.pone.0209025)
Supplement: S2 Appendix — (PDF) [file pone.0209025.s004.pdf]

S2 Appendix. A null model to account for the species tolerance to salinity.

The well-known trial swap null model (null model 1, NM1) reshuffles species occurrences from a pool of species irrespective of the abiotic factors that prevent random reassembly, which may lead to overly diverse communities. A second null model (the null model 2, NM2) was developped to simulate more realistic communities based on an abiotic factor (i.e. salinity) that strongly shapes fish occurrence and abundance within estuaries. Salinity tolerance was assessed at the species level from an external fish database.

1 Defining the salinity tolerance of fish species

1.1 A database on fish in French lagoons and estuaries

Beam trawl surveys have been carried out since 2005 in the transitional waters of metropolitan France to assess their ecological status as required under the European Water Framework Directive 2000/60/EC (Delpech et al., 2010 ; Teichert et al., 2017). The transitional waters include mainly estuaries of the Atlantic and the Channel coasts. The largest transitional hydrosystems (the Gironde, Loire and Seine estuaries) are subdivided into several ’waterbodies’ based on average salinity values. Salinity was measured at the end of every beam haul.

1.2 Characterization of the salinity tolerance

The salinity tolerance was not assessed at the ecophase level but only at the species level mainly because the smaller ecophases were not sampled efficiently with the fishing protocol. A species tolerance to salinity was characterized from the species frequency of occurrence among four predefined salinity classes : freshwater ($sal. < 0.5$), oligohaline ($0.5 \leq sal. < 5$), mesohaline ($5 \leq sal. < 18$), poly- and euhaline ($sal. \geq 18$). The assessment of salinity tolerance was based on a multi-step procedure (Fig 1).

The pattern of salinity tolerance of the 34 fish species sampled in the 13 dyked and intertidal sites is supplied in Table 1. The tolerance of *Dicentrarchus punctatus* to salinity was corrected *a posteriori* to match the pattern of *Dicentrarchux labrax* : the salinity class ’<0.5’ was removed from the tolerated classes of *D. punctatus* although the FO in this class was higher (12.8%) than 10% of the maximum FO.

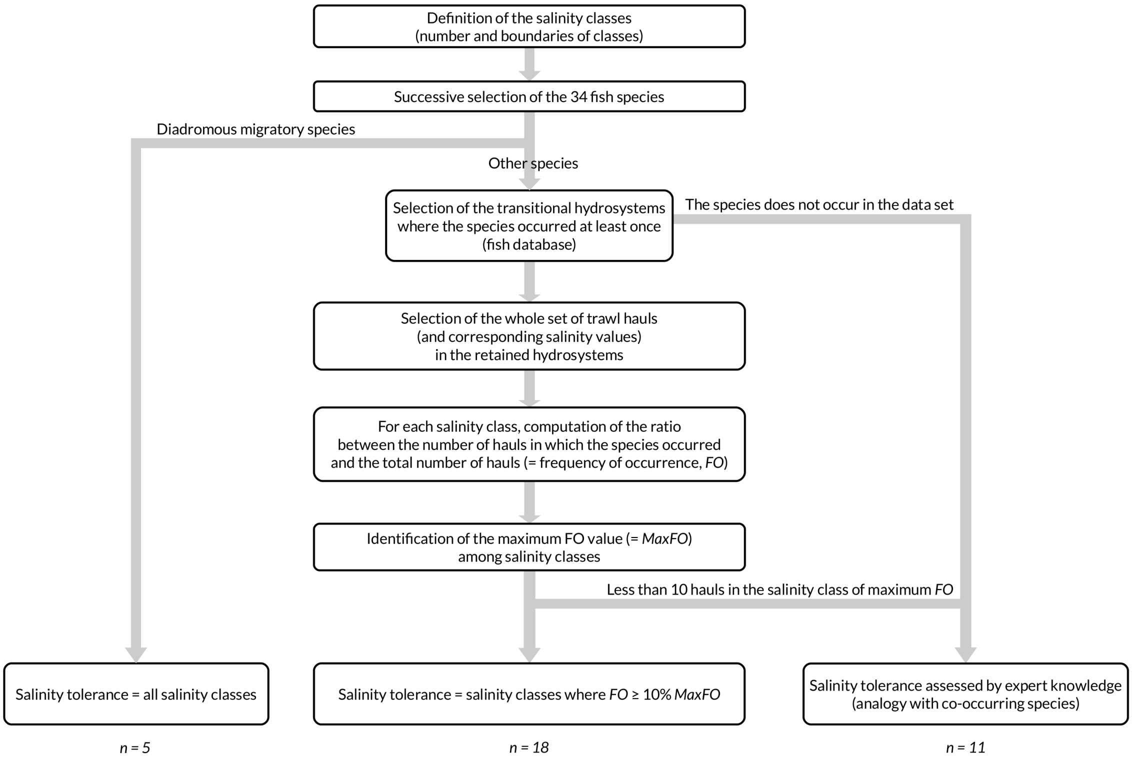

Figure 1 – Assessment of the salinity tolerance of fish species. n is the number of fish species evaluated in each of the three pathways.

2 Community simulation

Communities were simulated following the steps illustrated on Fig 2.

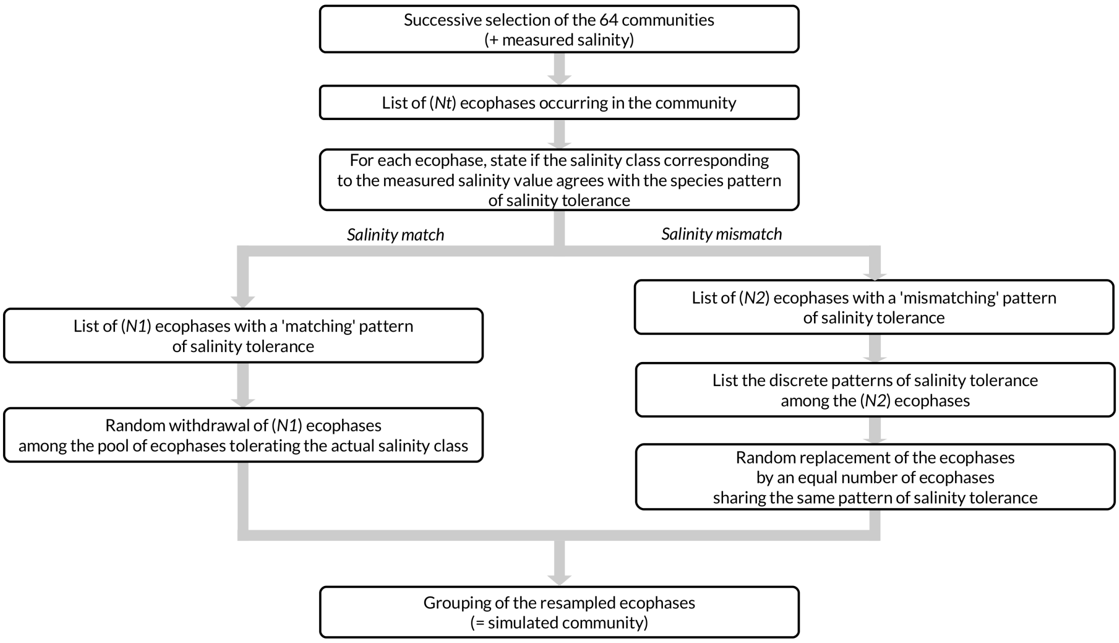

Figure 2 – Simulation of communities under the null model 2, accounting for the species tolerance to salinity.

Table 1 – The tolerance of fish species to salinity.

Species	Tolerated salinity classes				Method
	Freshwater	Oligohaline	Mesohaline	Poly- and euhaline	
Abramis brama	+	+	−	−	FO in salinity classes
Alosa fallax	+	+	+	+	Diadromous
Ameiurus melas	+	+	+	−	Expertise
Anguilla anguilla	+	+	+	+	Diadromous
Argyrosomus regius	−	−	+	+	FO in salinity classes
Blicca bjoerkna	+	+	+	−	FO in salinity classes
Carassius gibelio	+	+	−	−	Expertise
Chelon labrosus	−	+	+	+	Expertise
Ciliata mustela	−	+	+	+	FO in salinity classes
Conger conger	−	−	+	+	FO in salinity classes
Cyprinus carpio	+	+	+	−	Expertise
Dicentrarchus labrax	−	+	+	+	FO in salinity classes
Dicentrarchus punctatus	−	+	+	+	FO in salinity classes
Engraulis encrasicolus	−	+	+	+	FO in salinity classes
Gambusia holbrooki	+	+	+	+	Expertise
Gasterosteus aculeatus	+	+	+	+	Diadromous
Gymnocephalus cernuus	+	+	+	−	Expertise
Lepomis gibbosus	+	+	+	−	Expertise
Liza ramada	+	+	+	+	Diadromous
Platichthys flesus	+	+	+	+	Diadromous
Pomatoschistus microps	+	+	+	+	FO in salinity classes
Pomatoschistus minutus	+	+	+	+	FO in salinity classes
Pseudorasbora parva	+	+	+	−	Expertise
Rutilus rutilus	+	+	−	−	FO in salinity classes
Sander lucioperca	+	+	+	−	FO in salinity classes
Scardinius erythrophthalmus	+	−	−	−	Expertise
Silurus glanis	+	−	−	−	Expertise
Solea senegalensis	−	+	+	+	FO in salinity classes
Solea solea	−	+	+	+	FO in salinity classes
Sparus aurata	−	+	+	+	FO in salinity classes
Sprattus sprattus	−	+	+	+	FO in salinity classes
Squalius cephalus	+	+	−	−	FO in salinity classes
Syngnathus rostellatus	−	−	+	+	FO in salinity classes
Tinca tinca	+	+	−	−	Expertise

The pattern of salinity tolerance was described based on minimum frequency of occurrence (FO) in four predefined salinity classes.

References

Delpech, C., Courrat, A., Pasquaud, S., Lobry, J., Le Pape, O., Nicolas, D., Boët, P., Girardin, M., Lepage, M., 2010. Development of a fish-based index to assess the ecological quality of transitional waters : The case of French estuaries. Marine Pollution Bulletin 60, 908–918. https ://doi.org/10.1016/j.marpolbul.2010.01.001

Teichert, N., Pasquaud, S., Borja, A., Chust, G., Uriarte, A., Lepage, M., 2017. Living under stressful conditions : Fish life history strategies across environmental gradients in estuaries. Estuarine, Coastal and Shelf Science 188, 18–26. https ://doi.org/10.1016/j.ecss.2017.02.006
