# Supplementary material for: Change in fish functional diversity and assembly rules in the course of tidal marsh restoration
Source: PLoS One. 2018 Dec 19;13(12):e0209025. doi: 10.1371/journal.pone.0209025 (PMC6300267; doi:10.1371/journal.pone.0209025)
Supplement: S3 Appendix — (PDF) [file pone.0209025.s005.pdf]

S3 Appendix. Computation of the effect size.

'Effect size' is a way to measure the magnitude of the difference between two groups. Here, the purpose was to compare the functionnal diversity between an observed community and a set of simulated communities.

A popular method to express an effect size is the 'standardized effect size' (SES ; Gotelli and McCabe, 2002). Let FD be an index of functional diversity and n_{sim} the number of communities simulated under a null model. The standardized effect size (SES) is measured as follows :

$$SES = \frac{FD_{obs} - \overline{FD_{sim}}}{sd(FD_{sim})}$$

where $\overline{FD_{sim}}$ and $sd(FD_{sim})$ are the mean and the standard deviation of the simulated FD values, respectively.

Assigning a significance value to the SES relies on a normal distribution of the simulated values after centering and scaling. However, this condition is not always fulfilled (e.g., Bernard-Verdier et al., 2012).

Following Bernard-Verdier et al. (2012), we computed the effect size (ES) as :

$$ES = 2 \times (P - 0.5)$$

where :

$$P = \frac{\sum_{i=1}^{n_{sim}} \mathbf{1}_{FD_i < FD_{obs}} + \frac{\sum_{i=1}^{n_{sim}} \mathbf{1}_{FD_i = FD_{obs}}}{2}}{n_{sim} + 1}.$$

and $\mathbf{1}$ is the indicator function.

With this formula, ES varies between -1 and 1. Values close to 0 indicate non-significant differences between the observed and the simulated values.

References

Bernard-Verdier, M., Navas, M.-L., Vellend, M., Violle, C., Fayolle, A., Garnier, E., 2012. Community assembly along a soil depth gradient : contrasting patterns of plant trait convergence and divergence in a Mediterranean rangeland. J Ecol 100, 1422–1433. https ://doi.org/10.1111/1365-2745.12003

Gotelli, N.J., McCabe, D.J., 2002. Species Co-Occurrence : A Meta-Analysis of J. M. Diamond's Assembly Rules Model. Ecology 83, 2091–2096. https ://doi.org/10.1890/0012-9658(2002)083[2091 :SCOAMA]2.0.CO ;2
